# Supplementary material for: Inhaled ciclesonide in adults hospitalised with COVID-19: a randomised controlled open-label trial (HALT COVID-19)
Source: BMJ Open. 2023 Feb 22;13(2):e064374. doi: 10.1136/bmjopen-2022-064374 (PMC9950582; doi:10.1136/bmjopen-2022-064374)
Supplement: Supplementary data [file bmjopen-2022-064374supp001.pdf]

ONLINE APPENDIX

Brodin D, Tornhammar P, Ueda P, Krifors A, Westerlund E, Athlin S, Wojt S, Elvstam O, Neumann A, Elshani A, Giesecke J, Edvardsson J, Bunpuckdee S, Unge C, Larsson M, Johansson B, Ljungberg J, Lindell J, Hansson J, Blennow O, Andersson DP. Inhaled Ciclesonide in Adults Hospitalized with Covid-19: a Randomized Controlled Open-label Trial (HALT Covid-19).

Protocol changes and rationale

The trial was designed in the beginning of the covid-19 pandemic when data from randomized clinical trials of Covid-19 treatment were scarce. After trial initiation, treatments for patients with Covid-19 and hospitalization rates of such patients changed rapidly. Therefore, we made changes to the protocol and the trial was stopped early.

5 weeks after the start of patient inclusion in our study, in July 2020, the Recovery Collaborative group presented preliminary data[1] showing protective effects of dexamethasone treatment in patients hospitalized for covid-19; a subgroup analysis of this study indicated that the effect was driven by patients receiving invasive mechanical ventilation or oxygen therapy. These data, in combination with local experience from treating patients with Covid-19,[2] led to most patients receiving oxygen therapy with ≥ 4 L oxygen/min at the study hospitals being treated with systemic corticosteroids. As use of systemic corticosteroids was an exclusion criterion, the change in practice made a large proportion of the Covid-19 patients ineligible for participation.

Initially the trial was conducted at 4 hospitals. To increase the inclusion rate, 9 additional hospitals were included as study sites, although only 5 of them ended up recruiting patients to the study. We also removed the previous upper age limit of 85 years for inclusion and allowed for inclusion of patients based on a positive antigen test for SARS-CoV-2. Moreover, because some patients may start receiving oxygen therapy before hospital admission (e.g., at nursing homes before being transported

to the hospital) or a period after hospital admission (e.g., if the patient's condition deteriorated) and we aimed to include patients shortly after initiation of such therapy, we changed the inclusion criteria from hospitalization within 48 hours prior to enrollment to initiation of oxygen therapy no longer than 48 hours prior to enrollment.

All changes were approved by the Data Monitoring Committee, Ethical Review Authority and the Swedish Medical Products Agency and implemented from December 2020.

In June 2021, when 99 patients had been included in the study, a large and increasing proportion of the adult Swedish population had received vaccination for Covid-19. The number of patients hospitalized with Covid-19 had dropped substantially and there were none to only a few Covid-19 patients admitted to the study hospitals per week. We determined that it was unlikely that we would reach the intended sample size and asked the Data Monitoring Committee to convene for a meeting. Following the recommendation of the Data Monitoring Committee, the study was terminated early due to expected futility to meet total enrolment.

Inclusion and exclusion criteria[a]

Participants were eligible for inclusion if, at the time of study inclusion, they (1) were aged ≥18 years, (2) had a polymerase chain reaction confirmed SARS-CoV-2 infection or a positive antigen test for SARS-CoV-2, (3) were hospitalized at any of the study hospitals and (4) were receiving oxygen therapy with not more than 48 hours having passed since initiation of this treatment.

Patients were not eligible for inclusion if they (1) had a history of hypersensitivity to ciclesonide or other substances included in the treatment, (2) received ongoing treatment with inhaled or oral corticosteroids, ketokonazol, itrakonazol, ritonavir or nelfinavir, (3) received >8 L oxygen/min or >50 % oxygen with nasal high-flow therapy, (4) were receiving or under consideration for palliative care or had an expected survival of less than 72 h, (5) were expected to be admitted to an intensive care unit within 48 h, (6) had active or inactive pulmonary tuberculosis, severe liver failure (Child-Pugh C), pulmonary arterial hypertension or fibrosis, cognitive or physical impairment, (7) had insufficient language skills to understand information given about the study, (8) had been included in a clinical trial within 30 days, or (9) were women and pregnant, breastfeeding or did not agree to take highly effective contraceptive measures while receiving treatment plus an additional 7 days.

[a] The presentation of these inclusion and exclusion criteria have been modified for readability as compared with the version presented in the study protocol.

Appendix table 1 Number of participants included in the final study population by study center.

Study center	*n participants*
Danderyd Hospital	26
Capio S:t Göran Hospital	24
Karolinska University Hospital	21
Västmanland County Hospital	13
Örebro University Hospital[a]	6
Växsjö Central Hospital[a]	3
Halland County Hospital[a]	2
Östersund Hospital[a]	2
Visby Hospital[a]	1

[a] In the analyses adjusted for study center, these hospitals were categorized into one group.

Appendix table 2 Additionally adjusted model.

**Variable**	**Hazard ratio (95% CI) for termination of oxygen therapy**
Ciclesonide (vs standard care)	0.68 (0.43 to 1.09)
Age (per year increase)	0.97 (0.95 to 0.99)
Female (vs male)	0.81 (0.46 to 1.40)
Days since symptom onset (per day increase)	0.99 (0.93 to 1.06)
C-reactive protein (per mg/L increase)	1.00 (1.00 to 1.00)
White cell count (per 10^9/L increase)	1.07 (0.95 to 1.19)
Diabetes	0.84 (0.44 to 1.58)
Hypertension	1.42 (0.79 to 2.56)
Hyperlipidemia	0.86 (0.45 to 1.64)

The model was also adjusted for study center.

References

1. Group RC, Horby P, Lim WS, et al. Dexamethasone in Hospitalized Patients with Covid-19. *N Engl J Med* 2021;384(8):693-704. doi: 10.1056/NEJMoa2021436 [published Online First: 2020/07/18]
2. Kan B, Ahl M, Blennow O, et al. *Lakartidningen* 2020;117 [published Online First: 2020/10/07]
