# Supplementary material for: Inhaled ciclesonide in adults hospitalised with COVID-19: a randomised controlled open-label trial (HALT COVID-19)
Source: BMJ Open. 2023 Feb 22;13(2):e064374. doi: 10.1136/bmjopen-2022-064374 (PMC9950582; doi:10.1136/bmjopen-2022-064374)
Supplement: Author's manuscript [file bmjopen-2022-064374.draft_revisions.pdf]

BMJ Open

BMJ Open is committed to open peer review. As part of this commitment we make the peer review history of every article we publish publicly available.

When an article is published we post the peer reviewers' comments and the authors' responses online. We also post the versions of the paper that were used during peer review. These are the versions that the peer review comments apply to.

The versions of the paper that follow are the versions that were submitted during the peer review process. They are not the versions of record or the final published versions. They should not be cited or distributed as the published version of this manuscript.

BMJ Open is an open access journal and the full, final, typeset and author-corrected version of record of the manuscript is available on our site with no access controls, subscription charges or pay-per-view fees (http://bmjopen.bmj.com).

If you have any questions on BMJ Open's open peer review process please email info.bmjopen@bmj.com

BMJ Open

Inhaled Ciclesonide in Adults Hospitalized with Covid-19: a Randomized Controlled Open-label Trial (HALT Covid-19)

Journal:	*BMJ Open*
Manuscript ID	bmjopen-2022-064374
Article Type:	Original research
Date Submitted by the Author:	05-May-2022

Complete List of Authors:	Brodin, Daniel; Capio Sankt Görans Sjukhus<br>Tornhammar, Per; Karolinska Institute, Functional Area of Emergency Medicine<br>Ueda, Peter; Karolinska Institutet<br>Krifors, Anders; Karolinska Institutet, Department of Physiology and Pharmacology; Västmanlands sjukhus Västerås<br>Westerlund, Eli; Danderyd University Hospital, Department of Clinical Sciences<br>Athlin, Simon; Örebro University<br>Wojt, Sandra; Danderyd University Hospital, Department of Internal Medicine<br>Elvstam, Olof; Central Hospital Växjö, Department of Infectious Diseases<br>Neumann, Anca; Capio Sankt Görans Sjukhus<br>Elshani, Arsim; Karlskoga Hospital<br>Giesecke, Julia; Karolinska Universitetssjukhuset<br>Edvardsson-Källkvist, Jens; Karolinska University Hospital<br>Bunpuckdee, Sayam; Karolinska Institutet<br>Unge, Christian; Danderyd University Hospital, Department of Internal Medicine<br>Larsson, Martin; Karolinska Institutet Department of Clinical Science and Education Sodersjukhuset<br>Johansson, Björn; Halland County<br>Ljungberg, Johan; Halland County<br>Lindell, Jonas; Visby Hospital, Department of Infectious Diseases<br>Hansson, Johan; Östersund Hospital, Department of Infectious Diseases<br>Blennow, Ola; Karolinska University Hospital, Department of Infectious Diseases<br>Andersson, Daniel Peter; Karolinska University Hospital, Department of Medicine Huddinge H7, Karolinska Institutet; Karolinska Institute, Medicine (H7)

Keywords:	COVID-19, Clinical trials < THERAPEUTICS, Respiratory infections < THORACIC MEDICINE

SCHOLARONE™
Manuscripts

I, the Submitting Author has the right to grant and does grant on behalf of all authors of the Work (as defined in the below author licence), an exclusive licence and/or a non-exclusive licence for contributions from authors who are: i) UK Crown employees; ii) where BMJ has agreed a CC-BY licence shall apply, and/or iii) in accordance with the terms applicable for US Federal Government officers or employees acting as part of their official duties; on a worldwide, perpetual, irrevocable, royalty-free basis to BMJ Publishing Group Ltd ("BMJ") its licensees and where the relevant Journal is co-owned by BMJ to the co-owners of the Journal, to publish the Work in this journal and any other BMJ products and to exploit all rights, as set out in our licence.

The Submitting Author accepts and understands that any supply made under these terms is made by BMJ to the Submitting Author unless you are acting as an employee on behalf of your employer or a postgraduate student of an affiliated institution which is paying any applicable article publishing charge ("APC") for Open Access articles. Where the Submitting Author wishes to make the Work available on an Open Access basis (and intends to pay the relevant APC), the terms of reuse of such Open Access shall be governed by a Creative Commons licence – details of these licences and which Creative Commons licence will apply to this Work are set out in our licence referred to above.

Other than as permitted in any relevant BMJ Author's Self Archiving Policies, I confirm this Work has not been accepted for publication elsewhere, is not being considered for publication elsewhere and does not duplicate material already published. I confirm all authors consent to publication of this Work and authorise the granting of this licence.

For peer review only

Inhaled Ciclesonide in Adults Hospitalized with Covid-19: a Randomized Controlled Open-label Trial (HALT Covid-19)

Daniel Brodin[1]*, MD; Per Tornhammar[2,3]*, MD; Peter Ueda[4*¤], MD, PhD; Anders Krifors[5,6] MD; Eli Westerlund[7], MD, PhD; Simon Athlin[8], MD, PhD; Sandra Wojt[9], MD; Olof Elvstam[10], MD, PhD; Anca Neumann[1], MD; Arsim Elshani[11], MD; Julia Giesecke[3] MD; Jens Edvardsson-Källkvist[3], MD; Sayam Bunpuckdee[12], MD student; Christian Unge[9], MD, PhD; Martin Larsson[13,14], MD, PhD; Björn Johansson[15], MD; Johan Ljungberg[15], MD; Jonas Lindell[16], MD; Johan Hansson[17], MD, PhD; Ola Blennow[1,3,18#¤], MD, PhD, Daniel P Andersson[3,13#] MD, PhD

*Equal contributions

#Equal contributions

¤Corresponding author

Correspondence

Ola Blennow MD, PhD Address: Department of Infectious Diseases, Karolinska University Hospital, Huddinge, 14141 Stockholm Sweden

Email: ola.blennow@regionstockholm.se

Phone: +46739813617

Affiliations:

[1] Department of Internal Medicine, Capio St Göran´s Hospital, Stockholm, Sweden

[2] Functional Area of Emergency Medicine, Karolinska University Hospital Huddinge, Stockholm, Sweden

For peer review only - http://bmjopen.bmj.com/site/about/guidelines.xhtml

[3] Department of Medicine (H7), Karolinska Institutet, Karolinska University Hospital, Huddinge, 141 86 Stockholm, Sweden

[4] Clinical Epidemiology Division, Department of Medicine Solna, Karolinska Institutet, Stockholm, Sweden

[5] Department of Physiology and Pharmacology, Karolinska Institutet, Stockholm, Sweden.

[6] Centre for Clinical Research Västmanland, Uppsala University, Västmanland County Hospital, Västerås, Sweden.

[7] Department of Clinical Sciences, Karolinska Institutet, Danderyd Hospital, Stockholm, Sweden

[8] School of Medical Sciences, Faculty of Medicine, Örebro University, Örebro, Sweden

[9] Department of Internal Medicine, Danderyd Hospital, Stockholm, Sweden

[10] Department of Infectious Diseases, Växjö Central Hospital, Växjö, Sweden

[11] Department in Geriatric Medicine at Karlskoga Hospital, Karlskoga, Sweden

[12] At the time of the study: Department of Medicine (H7), Karolinska Institutet, Stockholm, Sweden

[13] Department of Endocrinology, Karolinska University Hospital, Stockholm, Sweden

[14] Department of Molecular Medicine and Surgery (K1), Karolinska Institutet, Stockholm, Sweden

[15] Department of Infectious Diseases Halland County Hospital Halmstad, Halmstad, Sweden

[16] Department of Infectious Diseases, Visby Hospital, Visby, Sweden

[17] Department of Infectious Diseases, Östersund Hospital, Östersund, Sweden

For peer review only

For peer review only - http://bmjopen.bmj.com/site/about/guidelines.xhtml

[18] Department of Infectious Diseases, Karolinska University Hospital, Stockholm, Sweden

Word count: 3002

Abstract word count: 244

For peer review only

For peer review only - http://bmjopen.bmj.com/site/about/guidelines.xhtml

Abstract

Objective: To assess the efficacy of ciclesonide in reducing the duration of oxygen therapy (an indicator of time to clinical improvement) among adults hospitalized with Covid-19.

Design: Multicenter, randomized, controlled, open-label trial.

Setting: 9 hospitals (3 academic hospitals and 6 non-academic hospitals) in Sweden between June 1, 2020, and May 17, 2021.

Participants: Adults hospitalized with Covid-19 and receiving oxygen therapy.

Intervention: Ciclesonide 320 µg twice daily for 14 days versus standard care.

Main outcome measures: Primary outcome was duration of oxygen therapy, an indicator of time to clinical improvement. Key secondary outcome was a composite of invasive mechanical ventilation/death.

Results: Data from 98 participants were analyzed (48 receiving ciclesonide and 50 receiving standard care; median (IQR) age, 59.5 (49-67) years; 67 (68%) male). Median (IQR) duration of oxygen therapy was 5.5 (3-9) days in the ciclesonide group and 4 (2-7) days in the standard care group (hazard ratio (HR) for termination of oxygen therapy 0.73 (95% CI 0.47-1.11), with the upper 95% CI being compatible with a 10% relative reduction in oxygen therapy duration, corresponding to a <1-day absolute reduction). Three participants in each group died/received invasive mechanical ventilation (HR 0.90 (95% CI 0.15-5.32)). The trial was discontinued early due to slow enrollment.

For peer review only - http://bmjopen.bmj.com/site/about/guidelines.xhtml

Conclusions: In hospitalized Covid-19 patients receiving oxygen therapy, this trial ruled out, with 0.95 confidence, a treatment effect of ciclesonide corresponding to more than a one-day reduction in duration of oxygen therapy. Ciclesonide is unlikely to improve this outcome meaningfully.

Strengths and limitations of this study

- This was a multicenter, randomized, controlled, open-label trial comparing treatment with the inhaled corticosteroid ciclesonide 320 µg twice daily for 14 days versus standard care.
- While inhaled corticosteroids have been assessed among non-hospitalized patients with Covid-19, data from studies on hospitalized Covid-19 patients with more severe disease are scarce.
- The trial was terminated early due to slow recruitment. Healthcare providers and participants were not blinded to treatment assignment.

For peer review only

For peer review only - http://bmjopen.bmj.com/site/about/guidelines.xhtml

Introduction

Patients with Covid-19 can develop acute respiratory failure that may require invasive mechanical ventilation, associated with high mortality. The unregulated inflammation in the lungs, poor oxygenation and pulmonary infiltrates characterizing severe Covid-19 have been considered as a type of acute respiratory distress syndrome (ARDS).[1, 2]

Prior to the Covid-19 pandemic, studies have indicated that inhaled corticosteroids may reduce the risk of ARDS. In a randomized controlled trial including 61 patients at risk of ARDS, none of the patients assigned to aerosolized budesonide/formoterol vs 7 assigned to placebo developed ARDS,[3] and 6 (20%) and 16 (53%) of the patients, respectively, received mechanical ventilation. In another trial including 60 patients with acute lung injury or ARDS, nebulized budesonide improved oxygenation and peak and plateau airway pressures, and reduced inflammatory markers.[4] Moreover, potentially protective and preventive effects of inhaled corticosteroids for ARDS is supported by animals models of lung injury,[5-8] and *in vitro* studies.[9]

Therefore, it could be hypothesized that inhaled corticosteroids may be beneficial for patients with severe Covid-19. The hypothesis is further supported by reports that inhaled corticosteroids reduce the epithelial expression of genes linked to SARS-CoV-2 entry into host cells.[10, 11] Among the inhaled corticosteroids, ciclesonide has been identified as a particularly promising treatment as it can suppress replication of SARS-CoV-2 *in vitro*.[12, 13]

While previous randomized controlled trials have assessed the effects of inhaled budesonide[14, 15] or ciclesonide[16, 17] in non-hospitalized Covid-19 patients, no study has been performed in hospitalized patients with more severe Covid-19.

For peer review only - http://bmjopen.bmj.com/site/about/guidelines.xhtml

This open-label randomized controlled trial investigated the effects of inhaled ciclesonide, compared to standard care, in adult patients hospitalized with Covid-19 and requiring oxygen therapy.

Methods

Study design

The HALT Covid-19 (inHALation of ciclesonide for Treatment of Covid-19) trial was a multicenter, open-label randomized controlled trial to assess the efficacy and safety of inhaled ciclesonide for the treatment of hospitalized patients with Covid-19 receiving oxygen therapy. The trial was conducted at 9 hospitals (3 academic hospitals and 6 non-academic hospitals) in Sweden between June 1, 2020, and May 17, 2021.

All participants provided written informed consent. The study was approved by the Swedish Ethical Review Authority (Ethics committee number 2020-02183) and the Swedish Medical Products Agency (Eudra-CT number 2020-001928-34) and registered at clinicaltrials.gov (NCT04381364).

Protocol changes and rationale

The trial was designed in the beginning of the Covid-19 pandemic. After trial initiation, treatments for, and hospitalization rates of, patients with Covid-19 changed rapidly. Therefore, we made protocol changes (described in detail in the Online Appendix) and the trial was stopped early.

For peer review only - http://bmjopen.bmj.com/site/about/guidelines.xhtml

For peer review only

In brief, we increased the number of study centers, removed the upper age limit (≤85 years) for patient inclusion, changed the inclusion criteria from ≤48 hours since hospital admission to ≤48 hours from initiation of oxygen therapy and allowed for patients to be included on the basis of a positive antigen test for SARS-Cov-2. All changes were approved by the Data Monitoring Committee, Ethical Review Authority and the Swedish Medical Products Agency and implemented from December 2020.

In June 2021, 99 patients had been included in the study, a large and increasing proportion of the adult Swedish population had received Covid-19 vaccination and hospitalizations for Covid-19 had dropped substantially. We determined that it was unlikely that the intended sample size would be reached and asked the Data Monitoring Committee to convene for a meeting. Following the recommendation of the Data Monitoring Committee, the study was terminated for futility to meet the targeted enrolment.

Participants

Based on observations from Covid-19 patients treated at the study centers, we expected that 85% of the standard care group would survive and terminate oxygen therapy within 30 days (median 8 days). We considered a 25% (2 days) reduction in the duration of oxygen therapy to be a clinically meaningful effect. We estimated that such an effect could be detected with α of 0.05, and 80% power if 446 participants (223 in each group) were enrolled.

For peer review only - http://bmjopen.bmj.com/site/about/guidelines.xhtml

Participants were eligible for inclusion if, they (1) were aged ≥18 years, (2) had a polymerase chain reaction confirmed SARS-CoV-2 infection or a positive SARS-CoV-2 antigen test from the upper respiratory tract, (3) were hospitalized at any of the study hospitals and (4) were receiving oxygen therapy, initiated within 48 hours before inclusion. Key exclusion criteria were ongoing treatment with inhaled or oral corticosteroids, oxygen therapy with >8 L oxygen/min or >50 % oxygen on nasal high-flow cannula, and ongoing or expected intensive care or palliative care (Online Appendix).

Randomization

Patients were randomized 1:1 in blocks of 8, stratified by sex and hospital to receive ciclesonide or standard care. The randomization sequence was prepared by a statistician not involved in the trial. Treatment allocation was provided through a web-based interface. The participants and the physicians treating them were unblinded to the treatment assignment.

Intervention

The treatment was 320 µg of inhaled ciclesonide (80 µg per actuation, for a total of 4 actuations, or 160 µg per actuation, for a total of 2 actuations) twice daily (total daily dose 640 µg) for 14 days. Ciclesonide was administered using a spacer (L'espace, Nordic Infucare, Stockholm Sweden). Participants randomized to ciclesonide received written instructions, including pictures, and practical instructions on how to use the inhalator and spacer; the first dose was taken under supervision. Ciclesonide was then prescribed in the participant's electronic medical record and each given dose during the hospitalization was recorded. Participants discharged before day 14

For peer review only - http://bmjopen.bmj.com/site/about/guidelines.xhtml

were instructed to continue the treatment at home for a total treatment duration of 14 days. Participants randomized to standard care did not receive any intervention related to the study. Physicians treating the participants were not given any restrictions concerning treatments during the study period. Participants who had been discharged were contacted by telephone after day 30 for a follow-up interview.

Outcomes

The primary outcome was duration of oxygen therapy (time to termination of oxygen therapy in days) up to 30 days from randomization. Oxygen therapy was defined as terminated on the day after which the patient did not receive oxygen therapy during at least 48 hours, while being alive. This outcome corresponded to clinical improvement for patients receiving oxygen therapy according to the World Health Organization clinical progression scale.[18]

The key secondary outcome was a composite of invasive mechanical ventilation and death up to 30 days after randomization. Other secondary outcomes were each component of the key secondary outcome, admission to an intensive care unit, discharge from the hospital and dyspnea in daily living at 30-35 days after randomization as evaluated by the mMRC (Modified Medical Research Council) dyspnea scale. The scale ranges from 0 to 4 with a higher score indicating more severe dyspnea.[19, 20]

Data on serious adverse events[21] were collected by review of electronic medical records. Information about non-serious adverse events associated with ciclesonide use (dryness of mouth, nausea and oral candidiasis) was reported using a paper-

For peer review only

based reporting form which was filled in by the treating physician. Information about non-serious adverse events occurring after hospital discharge was collected during the follow-up interview.

Data collection

Patient characteristics at baseline (comorbidities, comedications, clinical parameters) and study outcomes were obtained from electronic medical records. Investigators contacted participants after day 30 after randomization to ask them about non-serious adverse events and dyspnea in daily living (study outcome) at day 30-35 after randomization.

Statistical analysis

According to the pre-specified analysis plan in the study protocol, the analyses were performed by an investigator who had not been involved in the enrolment of participants and was blinded to treatment assignment. An intention-to-treat population was used. In the analysis of the duration of oxygen therapy, participants were followed from randomization to termination of oxygen therapy, death, or 30 days after randomization. Kaplan Meier cumulative incidence curves were generated to illustrate the cumulative incidence of termination of oxygen therapy in the ciclesonide and standard care groups. A Cox proportional hazard regression model, adjusted for study hospital (Appendix Table 1), age (continuous variable) and sex was used to estimate hazard ratios (HR) with 95% CI for time-to-event outcomes. Proportions and the absolute risk difference with 95% CI were presented for binary outcomes. Subgroup analyses were performed for the primary outcome by sex, age (<70 years

For peer review only - http://bmjopen.bmj.com/site/about/guidelines.xhtml

and ≥70 years) and duration of Covid-19 symptoms (<10 days and ≥10 days). In a per-protocol analysis of the primary outcome, participants assigned to ciclesonide were censored at the time of discontinuing treatment. The median mMRC score was compared using the Kruskal-Wallis test. A logistic regression model adjusted for study hospital, age and sex was used to compare the likelihood of reporting a mMRC score of 0 (dyspnea only with strenuous exercise).

95% CIs of ratios not including 1 and 95% CIs for absolute risk differences not including 0 were considered statistically significant. Secondary outcome analyses and subgroup analyses were considered hypothesis-generating and no adjustment for multiple testing was made. Analyses were performed using Stata version 16.1 (StataCorp).

Patient and Public involvement

No patients were involved in setting the research question, nor in the design, conduct, or interpretation of the study.

Results

Of the 99 participants who underwent randomization 48 were assigned to receive ciclesonide and 51 to standard care (Figure 1). One participant in the standard care group withdrew consent and was excluded from the analysis. Ninety-eight patients (48 in the ciclesonide group and 50 in the standard care group) were included in the

For peer review only - http://bmjopen.bmj.com/site/about/guidelines.xhtml

final analysis. All participants assigned to ciclesonide received the treatment at least once. None of the participants were lost to follow-up. The median age of participants was 59.5 (IQR 49, 67) years, 68% were men and the median duration of symptoms was 9 (IQR 8, 11) days. There were no relevant between-group differences in demographic characteristics, laboratory test results or comorbidities at enrollment (Table 1).

For peer review only

Table 1 Demographic and clinical characteristics of participants at study enrolment.

	**Total** (n=98)	**Ciclesonide** (n=48)	**Standard care** (n=50)
Age, median (IQR)	59.5 (49, 67)	61 (49, 67)	59 (49, 67)
Men, n (%)	67 (68)	34 (71)	33 (66)
Days since symptom onset, median (IQR)	9 (8, 11)	9 (7.5, 11.5)	10 (8, 11)
Body mass index in kg/m^2, median (IQR)	29.7 (25.6, 34.0)	28.7 (25.4, 34.0)	30.6 (26.8, 34.3)
Oxygen flow of oxygen therapy in L/min, median (IQR)	2 (1, 3)	2 (1, 3)	2 (1, 2)
Respiratory rate per minute, median (IQR)	20 (18, 24)	20 (19, 25)	20 (18, 23)
C-reactive protein in mg/L, median (IQR)	100 (56, 142)	103 (62, 164)	91.5 (45.5, 124.5)
White cell count in x 10^9/L, median IQR	5.7 (4.5, 7.0)	5.3 (4.3, 6.9)	6.1 (4.9, 7.0)
eGFR in mL/min/1.73m^2, median (IQR)	83 (70.5, 90)	81.5 (70, 90)	87 (73, 90)
**Coexisting conditions, n (%)**			
Diabetes mellitus	18 (18)	8 (17)	10 (20)
Hypertension[a]	45 (46)	22 (46)	23 (46)
Hyperlipidemia[b]	27 (28)	12 (25)	15 (30)
Chronic obstructive lung disease	3 (3)	1 (2)	2 (4)
Asthma	8 (8)	6 (13)	2 (4)
Current smoker	12 (12)	6 (13)	6 (12)
Ischemic heart disease	8 (8)	2 (4)	6 (12)
Heart failure	3 (3)	2 (4)	1 (2)
Atrial fibrillation	5 (5)	3 (6)	2 (4)

For peer review only - http://bmjopen.bmj.com/site/about/guidelines.xhtml

Cancer	10 (10)	5 (10)	5 (10)
Chronic kidney disease	9 (9)	5 (10)	4 (8)

[a] Diagnosis of hypertension or use of antihypertensive drugs

[b] Diagnosis of hyperlipidemia or use of lipid lowering therapy

Missing values were: n=1 for days since symptom onset, n=20 for body mass index, n=1 for oxygen flow of oxygen therapy, n=1 for body temperature, n=1 for heart rate, n=3 for respiratory rate, n=3 for C-reactive protein, n=7 for white cell count and n=22 for eGFR.

eGFR: estimated glomerular filtration rate

For peer review only

The results of primary and secondary outcome analyses are presented in Table 2. Kaplan-Meier estimates of the median duration of oxygen therapy were 5.5 (IQR 3, 9) days in the ciclesonide group and 4 (2, 7) days in the standard care group. (Figure 2). The HR for termination of oxygen therapy used to compare ciclesonide vs standard care showed that ciclesonide treatment was not statistically significantly associated with the duration of oxygen therapy (0.73 (95% CI 0.47 to 1.11)). The upper limit of the 95% CI was compatible with a maximum relative reduction[22] in duration of oxygen therapy of 10% (1-1/1.11) with ciclesonide, corresponding to a <1 day absolute reduction. In the per-protocol analysis, the HR for termination of oxygen therapy was 0.79 (95% CI 0.51 to 1.23) (Table 2).

Table 2 Outcomes.

	**Ciclesonide**	**Standard care**	**Difference[a]**
**Primary outcome**			
Duration of oxygen therapy, median (IQR) days	5.5 (3, 9)	4 (2, 7)	0.73 (0.47 to 1.11)
**Key secondary outcome**			
Death or invasive mechanical ventilation, n (%)	3 (6)	3 (6)	0 (-9 to 10)

For peer review only - http://bmjopen.bmj.com/site/about/guidelines.xhtml

Time to death or invasive mechanical ventilation, median (IQR) days	2 (2, 10)	4 (2, 7)	0.90 (0.15 to 5.32)
**Secondary outcomes**			
Death, n (%)	2 (4)	1 (2)	-
Invasive mechanical ventilation, n (%)	1 (2)	3 (6)	-
Admission to an intensive care unit, n (%)	4 (8)	4 (8)	-
mMRC dyspnea scale score at day 30-35, median (IQR)[b]	3 (2, 4)	3 (2, 4)	0.97
mMRC dyspnea scale score 0 at day 30-35, n (%)[b]	4 (9)	7 (15)	0.48 (0.11 to 2.04)
**Subgroup analyses**[c]			
Sex: Men			
Duration of oxygen therapy, median (IQR) days	5.5 (3, 9)	5 (2, 7)	0.61 (0.36 to 1.05)
Sex: Women			
Duration of oxygen therapy, median (IQR) days	5.5 (2, 7)	4 (2, 8)	0.91 (0.41 to 2.01)
Age group: <70 years			
Duration of oxygen therapy, median (IQR) days	5 (3, 7)	4 (2, 7)	0.77 (0.48 to 1.23)
Age group: ≥70 years			
Duration of oxygen therapy, median (IQR) days	9 (5, 10)	6 (5, 8)	0.37 (0.08 to 1.78)
Days since symptom onset: <10 days			
Duration of oxygen therapy, median (IQR) days	7 (3, 10)	4 (2, 5)	0.54 (0.28 to 1.03)
Days since symptom onset: ≥10 days			
Duration of oxygen therapy, median (IQR) days	5 (3, 6)	5 (3, 8)	0.97 (0.48 to 1.94)
**Per protocol analysis**[d]			
Duration of oxygen therapy, median (IQR) days	5 (3, 9)	4 (2, 7)	0.79 (0.51 to 1.23)

[a.] Differences are expressed as hazard ratios (95% CI) estimated using a Cox proportional hazards model for time to event outcomes and as absolute risk difference (95% CI) in percent for outcomes of absolute risk. The comparison of the mMRC dyspnea score was done using the Kruskal-Wallis test and the difference is expressed as a p-value. The comparison of the likelihood of reporting a mMRC score of 0 was done using a logistic regression model and the difference is expressed as an odds ratio (95% CI). Statistical testing for differences in proportions and time-to-event analyses were not performed for the secondary outcome events, including death, invasive mechanical ventilation, and admission to an intensive care unit due to few events.

For peer review only

For peer review only - http://bmjopen.bmj.com/site/about/guidelines.xhtml

[b.] Not including 1 participant in the standard care group and 2 participants in the ciclesonide group who died within 30 days of randomization and 1 participant in the standard care group and 1 participant in the ciclesonide group with missing data on this outcome.

[c.] The subgroup analyses included n=33 in the standard care group and n=34 in the ciclesonide group for men, n=17 in the standard care group and n=14 in the ciclesonide group for women, n=41 in the standard care group and n=37 in the ciclesonide group for those aged <70 years, n=9 in the standard care group and n=11 in the ciclesonide group for those aged ≥70 years, n=24 in the standard care group and n=27 in the ciclesonide group for those with <10 days since symptom onset , and n=25 in the standard care group and n=21 in the ciclesonide group for those with ≥10 days since symptom onset. 1 participant had missing data on days since symptom onset and was not included in the subgroup analysis.

[d.] In the per-protocol analysis for duration of oxygen therapy, patients assigned to ciclesonide were censored at the time of discontinuing treatment.

In total, 3 (6%) participants assigned to ciclesonide and 3 (6%) participants assigned to standard care experienced the key secondary outcome of mechanical invasive ventilation or death (absolute difference 0% (95% CI -10 to 9%; HR 0.90 (95% CI 0.15 to 5.32)). Median mMRC dyspnea score at 30-35 days after randomization was 3 (IQR 2, 4) in both groups (p-value for difference 0.97) (Table 2).

There were no statistically significant differences between those assigned to ciclesonide vs standard care in the primary outcome in any of the subgroup analyses by sex, age (<70 years and ≥70 years) and days since symptom onset (<10 days and ≥10 days) (Table 2).

There were no apparent differences between the groups in treatments that participants received after randomization (Table 3); 26 (54%) of the participants

For peer review only - http://bmjopen.bmj.com/site/about/guidelines.xhtml

assigned to ciclesonide and 22 (44%) of the participants in the standard care group received treatment with systemic corticosteroids after randomization.

Few serious adverse clinical events occurred during the study. The most frequently reported adverse event was dry mouth (7 (15%) participants in the ciclesonide group and 11 (22%) participants in the standard care group). Two participants assigned to ciclesonide and 0 in the placebo group reported that they experienced oral candidiasis (Table 3).

Table 3 Participants' treatments and adverse clinical events through day 30 after randomization.

	**Ciclesonide** (n=48)	**Standard care** (n=50)
**Received treatment, n (%)**		
Systemic corticosteroids	26 (54)	22 (44)
Remdesivir	4 (8)	5 (10)
Low-molecular-weight heparin	45 (94)	45 (90)
Oral anticoagulants	32 (67)	30 (60)
Vasopressors	4 (8)	3 (6)
Non-invasive mechanical ventilation	8 (17)	7 (14)
**Serious clinical events, n (%)**		
Renal failure	2 (4)	3 (6)
Cardiac arrest	1 (2)	0 (0)
New onset atrial fibrillation	0 (0)	1 (2)
Pulmonary embolism	4 (8)	2 (4)
Other thromboembolic events	0 (0)	1 (2)

For peer review only - http://bmjopen.bmj.com/site/about/guidelines.xhtml

For peer review only

Sepsis	3 (6)	2 (4)
Other serious event	1 (2)	0 (0)
**Non-serious adverse events, n (%)**		
Nausea	6 (13)	8 (16)
Dry mouth	7 (15)	11 (22)
Oral candidiasis	2 (4)	0 (0)
Other non-serious adverse event	3 (6)	1 (2)

Some pre-specified analyses were not performed due to small sample size or low number of events. These included statistical testing of differences in proportions and time-to-event analyses for non-key secondary outcomes, including death, invasive mechanical ventilation, and admission to an intensive care unit; the secondary outcome analyses of discharge from hospital; subgroup analyses for the secondary outcomes, and the primary outcome analysis after exclusion of participants who received invasive mechanical ventilation or died.

Discussion

In this randomized open-label, controlled trial, including 98 hospitalized Covid-19 patients with ongoing oxygen therapy, treatment with inhaled ciclesonide did not result in a statistically significant reduction in the duration of oxygen therapy, used as a measure of time to clinical improvement. The trial ruled out, with 0.95 confidence, treatments effects of ciclesonide corresponding to more than a one-day reduction in duration of oxygen therapy.

For peer review only

For peer review only - http://bmjopen.bmj.com/site/about/guidelines.xhtml

While previous randomized controlled trials have assessed effects of inhaled corticosteroids, including budesonide[14, 15] and ciclesonide[16, 17], in non-hospitalized patients with Covid-19, this is the first trial that includes hospitalized patients with more severe forms of the disease. In contrast to our hypothesis, the median duration of oxygen therapy was nominally longer among patients assigned to ciclesonide vs standard care (5.5 vs 4 days; HR for termination of oxygen therapy 0.73 (95% CI 0.47 to 1.11)). As such, the 95% CI indicates that,[22] even in the best case, ciclesonide may reduce the duration of oxygen therapy with only 10% (1-1/1.11; less than 1 day in our study) while it may in the worst case result in an over 2-fold increase. Thus, the results of this trial indicate that ciclesonide is unlikely to provide a clinically meaningful beneficial effect on the duration of oxygen therapy in hospitalized Covid-19 patients receiving oxygen therapy.

To date, 2 randomized controlled trials of ciclesonide in non-hospitalized patients with Covid-19 have been presented. In the CONTAIN study,[16] which was terminated early due to slow recruitment, 215 non-hospitalized patients with a median of 3 days symptom duration were randomized to combination treatment with intranasal and inhaled ciclesonide or placebo. No statistically significant difference between the groups was observed for the primary endpoint, resolution of respiratory symptoms at day 7 after randomization, which was reached by 40% of the patients in the treatment group vs 35% in the placebo group (adjusted risk difference of 5.5% (95% CI -7.8% to 18.8%).[16] Six (6%) patients assigned to ciclesonide vs 3 (3%) in the placebo group were hospitalized within 14 days; none died. In another clinical trial of ciclesonide, including 400 non-hospitalized patients with Covid-19,[17] randomization to ciclesonid vs placebo did not result in a reduced time to alleviation of all Covid-19

For peer review only - http://bmjopen.bmj.com/site/about/guidelines.xhtml

related symptoms. However, in secondary outcome analyses, patients assigned to ciclesonide had fewer emergency department visits or hospital admissions for reasons related to COVID-19 (odds ratio, 0.18, 95% CI, 0.04 to 0.85).

In addition, 2 randomized clinical trials of the inhaled corticosteroid budesonide in non-hospitalized patients with Covid-19 have been presented. The STOIC trial was an open-label trial comparing inhaled budesonide vs standard care in 146 Covid-19 patients with mild symptoms.[14] Compared to standard care, budesonide treatment led to a statistically significant reduction in Covid-19-related emergency department assessment and hospitalization (difference in proportions 0.123 (95% CI 0.043 to 0.218).[14] Furthermore, budesonide treatment was associated with 1 day shorter time to clinical recovery. The PRINCIPLE trial was another open-label trial that included 4700 primary care patients at high risk of developing severe Covid-19 (1073 randomized to budesonide treatment; 1988 to standard care; 1639 to other treatments).[15] Compared to standard care, randomization to budesonide led to a shorter time to self-reported recovery (difference 2.94 days (95% Bayesian credible interval 1.19 to 5.12) and a reduced likelihood of hospital admission or death, although the results for the latter outcomes did not meet the superiority threshold.

Taken together, the previous studies indicate that inhaled corticosteroids might be useful for preventing deterioration of Covid-19 in non-hospitalized patients with mild symptoms. It is possible that the low likelihood of benefit associated with ciclesonide treatment observed in our study reflects the more severe pulmonary inflammation in our study population, as indicated by the need for hospitalization with oxygen therapy and a median symptom duration of 9 days: at such stages of disease progression, it

For peer review only - http://bmjopen.bmj.com/site/about/guidelines.xhtml

could be speculated that pulmonary administration of corticosteroids may not suffice to confer benefit and that systemic treatment is needed. Accordingly, in the Recovery trial of hospitalized Covid-19 patients,[23] dexamethasone treatment reduced risk of death and the time to discharge from hospital, with these benefits primarily being observed among patients receiving oxygen therapy or invasive mechanical ventilation at baseline.

Similar to other clinical trials including patients with Covid-19,[15, 23, 24] we used a pragmatic, open-label design. With this design, we intended to assess the effect of adding ciclesonide to standard care, rather than to examine the effect of ciclesonide compared to placebo. The research question that our study aimed to answer was "what is the effect of using ciclesonide as an addition to standard care as compared with standard care alone?" While this is a research question of relevance to clinical decision-making, the open-label design and the possible expectations of effect among both patients[25] and physicians might have affected the outcomes in our study, including when to terminate oxygen therapy. Another limitation of our study is that we were unable to recruit the intended number of patients due to the substantial decrease in hospitalized Covid-19 patients in Sweden during 2021. Importantly, the study could not provide much information regarding the key secondary outcome of death or invasive mechanical intervention. Further research in hospitalized Covid-19 patients is needed to determine the potential effect of ciclesonide treatment on these outcomes. Moreover, it is a possibility that effects of ciclesonide differ as compared to other inhaled corticosteroids (e.g., budesonide). Finally, results from the Recovery Trial were released 5 weeks after the initiation of our study and around half of the patients in both the ciclesonide group and the control group received systemic

For peer review only - http://bmjopen.bmj.com/site/about/guidelines.xhtml

corticosteroids after randomization. Further studies would be needed to assess the comparative effectiveness and safety of ciclesonide vs systemic corticosteroids.

Conclusions

In this open-label randomized controlled trial in patients hospitalized with Covid-19 and receiving oxygen therapy, the findings indicated that treatment with ciclesonide vs standard care is unlikely to result in a clinically meaningful reduction in the duration of oxygen therapy.

Acknowledgements

We would like to thank the following individuals that did not qualify for authorship but contributed to the study: Dr Oscar Bakhouch (Skaraborg Hospital), Dr Eva-Marie Boman and Dr Anders Lundqvist (Southern Älvsborg Hospital).

Contributorship statement

DB, PU, PT, OB and DPA conceived the study and were responsible for the methods. OB and DPA were responsible for the study conduct. DB, PU, OB and DPA were responsible for the financing. PT validated the data. PU performed the main analysis. DPA, PU and PT wrote the original draft of the manuscript. All authors wrote, reviewed, and edited the manuscript. OB and DPA supervised the study. DB, DPA, PU, PT and OB were responsible for administration of the project. DPA and OB are the guarantors. DB, PT, AK, EW, SA, SW, OE, AN, AE, JG, JEK, BJ, JL, JLI, JH, OB and DPA enrolled participants in the study. The corresponding author attests that

For peer review only - http://bmjopen.bmj.com/site/about/guidelines.xhtml

all listed authors meet authorship criteria and that no others meeting the criteria have been omitted.

Competing interests

This study received non-financial support from COVIS Pharma (study drug donation). The authors have no conflict of interest to disclose.

Funding/Support

This study was funded by the Swedish Heart and Lung Foundation (Number 20200421), The Axel and Margaret Ax:son Johnson Foundation (N/A), CIMED (N/A), and Strategic Research Program at Karolinska Institutet (Number 961507), the Stockholm County Council (Number: 954970, 963296, 962029), and the Västmanland County Council (Grant nr LTV-938409). PU was supported by grants from the Strategic Research Program in Epidemiology at Karolinska Institutet (N/A), and a Faculty Funded Career Position at Karolinska Institutet (N/A).

The funders had no role in the study design, conduct, collection, management, analysis, interpretation of data, writing or reviewing the manuscript or decision to submit the manuscript for publication. The study drug was donated by COVIS Pharma but COVIS pharma did not participate in any other part of the study.

Data sharing statement

No additional data available.

Ethics Approval

For peer review only

All participants provided written informed consent. The study was approved by the Swedish Ethical Review Authority (Ethics committee number 2020-02183) and the Swedish Medical Products Agency (Eudra-CT number 2020-001928-34) and registered at clinicaltrials.gov (NCT04381364).

References

1. Gibson PG, Qin L, Puah SH. COVID-19 acute respiratory distress syndrome (ARDS): clinical features and differences from typical pre-COVID-19 ARDS. Med J Aust **2020**; 213:54-6 e1.

2. Bos LDJ. COVID-19-related Acute Respiratory Distress Syndrome: Not So Atypical. Am J Respir Crit Care Med **2020**; 202:622-4.

3. Festic E, Carr GE, Cartin-Ceba R, et al. Randomized Clinical Trial of a Combination of an Inhaled Corticosteroid and Beta Agonist in Patients at Risk of Developing the Acute Respiratory Distress Syndrome. Crit Care Med **2017**; 45:798-805.

4. Mohamed HS, Meguid MM. Effect of nebulized budesonide on respiratory mechanics and oxygenation in acute lung injury/acute respiratory distress syndrome: Randomized controlled study. Saudi J Anaesth **2017**; 11:9-14.

5. Jansson AH, Eriksson C, Wang X. Effects of budesonide and N-acetylcysteine on acute lung hyperinflation, inflammation and injury in rats. Vascul Pharmacol **2005**; 43:101-11.

6. Forsgren PE, Modig JA, Dahlback CM, Axelsson BI. Prophylactic treatment with an aerosolized corticosteroid liposome in a porcine model of early ARDS induced by endotoxaemia. Acta Chir Scand **1990**; 156:423-31.

For peer review only - http://bmjopen.bmj.com/site/about/guidelines.xhtml

7. Wang J, Zhang L, Walther SM. Administration of aerosolized terbutaline and budesonide reduces chlorine gas-induced acute lung injury. J Trauma **2004**; 56:850-62.

8. Walther S, Jansson I, Berg S, Lennquist S. Pulmonary granulocyte accumulation is reduced by nebulized corticosteroid in septic pigs. Acta Anaesthesiol Scand **1992**; 36:651-5.

9. Yamaya M, Nishimura H, Deng X, et al. Inhibitory effects of glycopyrronium, formoterol, and budesonide on coronavirus HCoV-229E replication and cytokine production by primary cultures of human nasal and tracheal epithelial cells. Respir Investig **2020**; 58:155-68.

10. Peters MC, Sajuthi S, Deford P, et al. COVID-19-related Genes in Sputum Cells in Asthma. Relationship to Demographic Features and Corticosteroids. Am J Respir Crit Care Med **2020**; 202:83-90.

11. Milne S, Li X, Yang CX, et al. Inhaled corticosteroids downregulate SARS-CoV-2-related genes in COPD: results from a randomised controlled trial. Eur Respir J **2021**; 58.

12. Jeon S, Ko M, Lee J, et al. Identification of Antiviral Drug Candidates against SARS-CoV-2 from FDA-Approved Drugs. Antimicrob Agents Chemother **2020**; 64.

13. Kimura H, Kurusu H, Sada M, et al. Molecular pharmacology of ciclesonide against SARS-CoV-2. J Allergy Clin Immunol **2020**; 146:330-1.

14. Ramakrishnan S, Nicolau DV, Jr., Langford B, et al. Inhaled budesonide in the treatment of early COVID-19 (STOIC): a phase 2, open-label, randomised controlled trial. Lancet Respir Med **2021**; 9:763-72.

For peer review only

For peer review only - http://bmjopen.bmj.com/site/about/guidelines.xhtml

15. Yu LM, Bafadhel M, Dorward J, et al. Inhaled budesonide for COVID-19 in people at high risk of complications in the community in the UK (PRINCIPLE): a randomised, controlled, open-label, adaptive platform trial. Lancet **2021**; 398:843-55.

16. Ezer N, Belga S, Daneman N, et al. Inhaled and intranasal ciclesonide for the treatment of covid-19 in adult outpatients: CONTAIN phase II randomised controlled trial. BMJ **2021**; 375:e068060.

17. Clemency BM, Varughese R, Gonzalez-Rojas Y, et al. Efficacy of Inhaled Ciclesonide for Outpatient Treatment of Adolescents and Adults With Symptomatic COVID-19: A Randomized Clinical Trial. JAMA Intern Med **2021**.

18. Characterisation WHOWGotC, Management of C-i. A minimal common outcome measure set for COVID-19 clinical research. Lancet Infect Dis **2020**; 20:e192-e7.

19. Mahler DA, Wells CK. Evaluation of clinical methods for rating dyspnea. Chest **1988**; 93:580-6.

20. Bestall JC, Paul EA, Garrod R, Garnham R, Jones PW, Wedzicha JA. Usefulness of the Medical Research Council (MRC) dyspnoea scale as a measure of disability in patients with chronic obstructive pulmonary disease. Thorax **1999**; 54:581-6.

21. Directive 2001/20/EC of the European Parliament and of the Council of 4 April 2001 on the approximation of the laws, regulations and administrative provisions of the member states relating to the implementation of good clinical practice in the conduct of clinical trials on medicinal products for human use. Med Etika Bioet **2002**; 9:12-9.

22. Hawkins AT, Samuels LR. Use of Confidence Intervals in Interpreting Nonstatistically Significant Results. JAMA **2021**; 326:2068-9.

23. Group RC, Horby P, Lim WS, et al. Dexamethasone in Hospitalized Patients with Covid-19. N Engl J Med **2021**; 384:693-704.

For peer review only

24. Investigators R-C, Gordon AC, Mouncey PR, et al. Interleukin-6 Receptor Antagonists in Critically Ill Patients with Covid-19. N Engl J Med **2021**; 384:1491-502.

25. Wechsler ME, Kelley JM, Boyd IO, et al. Active albuterol or placebo, sham acupuncture, or no intervention in asthma. N Engl J Med **2011**; 365:119-26.

For peer review only

For peer review only - http://bmjopen.bmj.com/site/about/guidelines.xhtml

Figure 1 Flow diagram for study participants.

Figure 2 Time to termination of oxygen therapy.

For peer review only

For peer review only - http://bmjopen.bmj.com/site/about/guidelines.xhtml

Figure 1

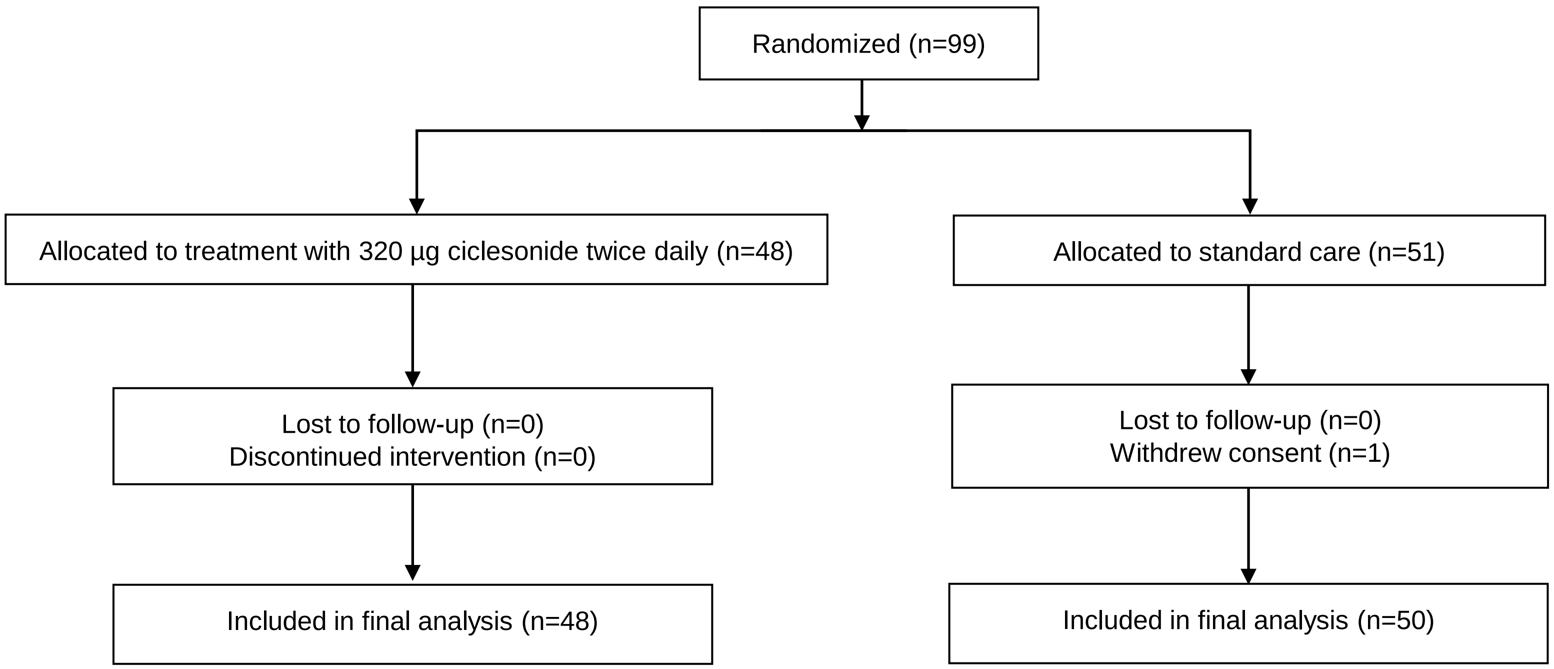

For peer review only - http://bmjopen.bmj.com/site/about/guidelines.xhtml

Figure 2 Time to termination of oxygen therapy.

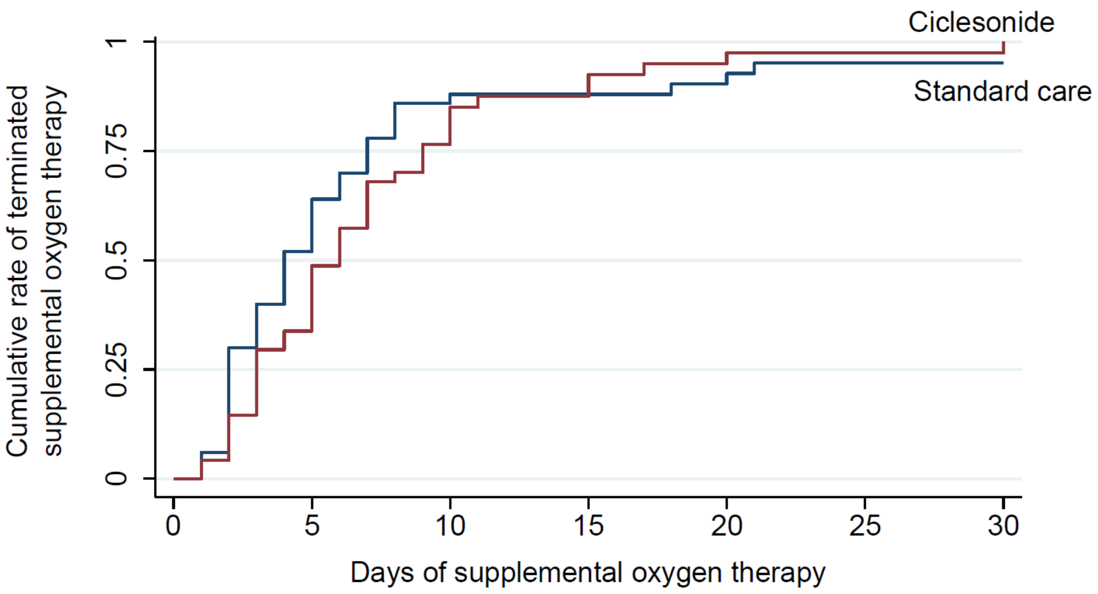

No at risk

Standard care	50	24	7	6	4	2	2
Ciclesonide	48	31	11	5	2	1	1

ew only

ONLINE APPENDIX

Brodin D, Tornhammar P, Ueda P, Krifors A, Westerlund E, Athlin S, Wojt S, Elvstam O, Neumann A, Elshani A, Giesecke J, Edvardsson J, Bunpuckdee S, Unge C, Larsson M, Johansson B, Ljungberg J, Lindell J, Hansson J, Blennow O, Andersson DP. Inhaled Ciclesonide in Adults Hospitalized with Covid-19: a Randomized Controlled Open-label Trial (HALT Covid-19).

For peer review only

Protocol changes and rationale

The trial was designed in the beginning of the covid-19 pandemic when data from randomized clinical trials of Covid-19 treatment were scarce. After trial initiation, treatments for patients with Covid-19 and hospitalization rates of such patients changed rapidly. Therefore, we made changes to the protocol and the trial was stopped early.

5 weeks after the start of patient inclusion in our study, in July 2020, the Recovery Collaborative group presented preliminary data[1] showing protective effects of dexamethasone treatment in patients hospitalized for covid-19; a subgroup analysis of this study indicated that the effect was driven by patients receiving invasive mechanical ventilation or oxygen therapy. These data, in combination with local experience from treating patients with Covid-19,[2] led to most patients receiving oxygen therapy with ≥ 4 L oxygen/min at the study hospitals being treated with systemic corticosteroids. As use of systemic corticosteroids was an exclusion criterion, the change in practice made a large proportion of the Covid-19 patients ineligible for participation.

Initially the trial was conducted at 4 hospitals. To increase the inclusion rate, 9 additional hospitals were included as study sites, although only 5 of them ended up recruiting patients to the study. We also removed the previous upper age limit of 85 years for inclusion and allowed for inclusion of patients based on a positive antigen test for SARS-CoV-2. Moreover, because some patients may start receiving oxygen therapy before hospital admission (e.g., at nursing homes before being transported

For peer review only

to the hospital) or a period after hospital admission (e.g., if the patient's condition deteriorated) and we aimed to include patients shortly after initiation of such therapy, we changed the inclusion criteria from hospitalization within 48 hours prior to enrollment to initiation of oxygen therapy no longer than 48 hours prior to enrollment.

All changes were approved by the Data Monitoring Committee, Ethical Review Authority and the Swedish Medical Products Agency and implemented from December 2020.

In June 2021, when 99 patients had been included in the study, a large and increasing proportion of the adult Swedish population had received vaccination for Covid-19. The number of patients hospitalized with Covid-19 had dropped substantially and there were none to only a few Covid-19 patients admitted to the study hospitals per week. We determined that it was unlikely that we would reach the intended sample size and asked the Data Monitoring Committee to convene for a meeting. Following the recommendation of the Data Monitoring Committee, the study was terminated early due to expected futility to meet total enrolment.

Inclusion and exclusion criteria[a]

Participants were eligible for inclusion if, at the time of study inclusion, they (1) were aged ≥18 years, (2) had a polymerase chain reaction confirmed SARS-CoV-2 infection or a positive antigen test for SARS-CoV-2, (3) were hospitalized at any of the study hospitals and (4) were receiving oxygen therapy with not more than 48 hours having passed since initiation of this treatment.

Patients were not eligible for inclusion if they (1) had a history of hypersensitivity to ciclesonide or other substances included in the treatment, (2) received ongoing treatment with inhaled or oral corticosteroids, ketokonazol, itrakonazol, ritonavir or nelfinavir, (3) received >8 L oxygen/min or >50 % oxygen with nasal high-flow therapy, (4) were receiving or under consideration for palliative care or had an expected survival of less than 72 h, (5) were expected to be admitted to an intensive care unit within 48 h, (6) had active or inactive pulmonary tuberculosis, severe liver failure (Child-Pugh C), pulmonary arterial hypertension or fibrosis, cognitive or physical impairment, (7) had insufficient language skills to understand information given about the study, (8) had been included in a clinical trial within 30 days, or (9) were women and pregnant, breastfeeding or did not agree to take highly effective contraceptive measures while receiving treatment plus an additional 7 days.

[a] The presentation of these inclusion and exclusion criteria have been modified for readability as compared with the version presented in the study protocol.

For peer review only

Appendix table 1 Number of participants included in the final study population by study center.

Study center	*n participants*
Danderyd Hospital	26
Capio S:t Göran Hospital	24
Karolinska University Hospital	21
Västmanland County Hospital	13
Örebro University Hospital[a]	6
Växsjö Central Hospital[a]	3
Halland County Hospital[a]	2
Östersund Hospital[a]	2
Visby Hospital[a]	1

[a] In the analyses adjusted for study center, these hospitals were categorized into one group.

For peer review only

References

1. RECOVERY Collaborative Group, Horby P, Lim WS, et al. Dexamethasone in Hospitalized Patients with Covid-19. *N Engl J Med*. Feb 25 2021;384(8):693-704. doi:10.1056/NEJMoa2021436
2. Kan B, Ahl M, Blennow O, et al. *Lakartidningen*. Oct 6 2020;117 Tidiga erfarenheter av steroidbehandling vid covid-19.
3. Fu Y, Cheng Y, Wu Y. Understanding SARS-CoV-2-Mediated Inflammatory Responses: From Mechanisms to Potential Therapeutic Tools. *Virol Sin*. Jun 2020;35(3):266-271. doi:10.1007/s12250-020-00207-4
4. Zhai P, Ding Y, Wu X, Long J, Zhong Y, Li Y. The epidemiology, diagnosis and treatment of COVID-19. *Int J Antimicrob Agents*. May 2020;55(5):105955. doi:10.1016/j.ijantimicag.2020.105955
5. Liu XX, Zhu XM, Miao Q, Ye HY, Zhang ZY, Li YM. Hyperglycemia induced by glucocorticoids in nondiabetic patients: a meta-analysis. *Ann Nutr Metab*. 2014;65(4):324-32. doi:10.1159/000365892
6. Patten SB, Neutel CI. Corticosteroid-induced adverse psychiatric effects: incidence, diagnosis and management. *Drug Saf*. Feb 2000;22(2):111-22. doi:10.2165/00002018-200022020-00004
7. Dubovsky AN, Arvikar S, Stern TA, Axelrod L. The neuropsychiatric complications of glucocorticoid use: steroid psychosis revisited. *Psychosomatics*. Mar-Apr 2012;53(2):103-15. doi:10.1016/j.psym.2011.12.007

For peer review only

CONSORT 2010 checklist of information to include when reporting a randomised trial*

Section/Topic	Item No	Checklist item	Reported on page No
**Title and abstract**			
	1a	Identification as a randomised trial in the title	1
	1b	Structured summary of trial design, methods, results, and conclusions (for specific guidance see CONSORT for abstracts)	3
**Introduction**			
Background and objectives	2a	Scientific background and explanation of rationale	5,6
	2b	Specific objectives or hypotheses	5-7
**Methods**			
Trial design	3a	Description of trial design (such as parallel, factorial) including allocation ratio	6-9
	3b	Important changes to methods after trial commencement (such as eligibility criteria), with reasons	6,7,9
Participants	4a	Eligibility criteria for participants	7,8
	4b	Settings and locations where the data were collected	6,7
Interventions	5	The interventions for each group with sufficient details to allow replication, including how and when they were actually administered	6-11
Outcomes	6a	Completely defined pre-specified primary and secondary outcome measures, including how and when they were assessed	9-11
	6b	Any changes to trial outcomes after the trial commenced, with reasons	6,7
Sample size	7a	How sample size was determined	6-9
	7b	When applicable, explanation of any interim analyses and stopping guidelines	6,7
Randomisation:			
Sequence generation	8a	Method used to generate the random allocation sequence	8
	8b	Type of randomisation; details of any restriction (such as blocking and block size)	8
Allocation concealment mechanism	9	Mechanism used to implement the random allocation sequence (such as sequentially numbered containers), describing any steps taken to conceal the sequence until interventions were assigned	8
Implementation	10	Who generated the random allocation sequence, who enrolled participants, and who assigned participants to interventions	8
Blinding	11a	If done, who was blinded after assignment to interventions (for example, participants, care providers, those	8,11

For peer review only

For peer review only - http://bmjopen.bmj.com/site/about/guidelines.xhtml

		assessing outcomes) and how	
	11b	If relevant, description of the similarity of interventions	-
Statistical methods	12a	Statistical methods used to compare groups for primary and secondary outcomes	10,11
	12b	Methods for additional analyses, such as subgroup analyses and adjusted analyses	10,11
**Results**			
Participant flow (a diagram is strongly recommended)	13a	For each group, the numbers of participants who were randomly assigned, received intended treatment, and were analysed for the primary outcome	11-13
	13b	For each group, losses and exclusions after randomisation, together with reasons	11-13
Recruitment	14a	Dates defining the periods of recruitment and follow-up	6,7
	14b	Why the trial ended or was stopped	7
Baseline data	15	A table showing baseline demographic and clinical characteristics for each group	Yes
Numbers analysed	16	For each group, number of participants (denominator) included in each analysis and whether the analysis was by original assigned groups	Yes
Outcomes and estimation	17a	For each primary and secondary outcome, results for each group, and the estimated effect size and its precision (such as 95% confidence interval)	Yes
	17b	For binary outcomes, presentation of both absolute and relative effect sizes is recommended	-
Ancillary analyses	18	Results of any other analyses performed, including subgroup analyses and adjusted analyses, distinguishing pre-specified from exploratory	10,11
Harms	19	All important harms or unintended effects in each group (for specific guidance see CONSORT for harms)	13
**Discussion**			
Limitations	20	Trial limitations, addressing sources of potential bias, imprecision, and, if relevant, multiplicity of analyses	13-17
Generalisability	21	Generalisability (external validity, applicability) of the trial findings	13-17
Interpretation	22	Interpretation consistent with results, balancing benefits and harms, and considering other relevant evidence	13-17
**Other information**			
Registration	23	Registration number and name of trial registry	6
Protocol	24	Where the full trial protocol can be accessed, if available	-
Funding	25	Sources of funding and other support (such as supply of drugs), role of funders	18,19

*We strongly recommend reading this statement in conjunction with the CONSORT 2010 Explanation and Elaboration for important clarifications on all the items. If relevant, we also recommend reading CONSORT extensions for cluster randomised trials, non-inferiority and equivalence trials, non-pharmacological treatments, herbal interventions, and pragmatic trials. Additional extensions are forthcoming: for those and for up to date references relevant to this checklist, see www.consort-statement.org.

For peer review only - http://bmjopen.bmj.com/site/about/guidelines.xhtml

BMJ Open

Inhaled Ciclesonide in Adults Hospitalized with Covid-19: a Randomized Controlled Open-label Trial (HALT Covid-19)

Journal:	*BMJ Open*
Manuscript ID	bmjopen-2022-064374.R1
Article Type:	Original research
Date Submitted by the Author:	08-Nov-2022
Complete List of Authors:	Brodin, Daniel; Capio Sankt Görans Sjukhus<br>Tornhammar, Per; Karolinska Institute, Functional Area of Emergency Medicine<br>Ueda, Peter; Karolinska Institutet<br>Krifors, Anders; Karolinska Institutet, Department of Physiology and Pharmacology; Västmanlands sjukhus Västerås<br>Westerlund, Eli; Danderyd University Hospital, Department of Clinical Sciences<br>Athlin, Simon; Örebro University<br>Wojt, Sandra; Danderyd University Hospital, Department of Internal Medicine<br>Elvstam, Olof; Central Hospital Växjö, Department of Infectious Diseases<br>Neumann, Anca; Capio Sankt Görans Sjukhus<br>Elshani, Arsim; Karlskoga Hospital<br>Giesecke, Julia; Karolinska Universitetssjukhuset<br>Edvardsson-Källkvist, Jens; Karolinska University Hospital<br>Bunpuckdee, Sayam; Karolinska Institutet<br>Unge, Christian; Danderyd University Hospital, Department of Internal Medicine<br>Larsson, Martin; Karolinska Institutet Department of Clinical Science and Education Sodersjukhuset<br>Johansson, Björn; Halland County<br>Ljungberg, Johan; Halland County<br>Lindell, Jonas; Visby Hospital, Department of Infectious Diseases<br>Hansson, Johan; Östersund Hospital, Department of Infectious Diseases<br>Blennow, Ola; Karolinska University Hospital, Department of Infectious Diseases<br>Andersson, Daniel Peter; Karolinska University Hospital, Department of Medicine Huddinge H7, Karolinska Institutet; Karolinska Institute, Medicine (H7)
**Primary Subject Heading**:	Infectious diseases
Secondary Subject Heading:	Respiratory medicine
Keywords:	COVID-19, Clinical trials < THERAPEUTICS, Respiratory infections < THORACIC MEDICINE

SCHOLARONE™
Manuscripts

I, the Submitting Author has the right to grant and does grant on behalf of all authors of the Work (as defined in the below author licence), an exclusive licence and/or a non-exclusive licence for contributions from authors who are: i) UK Crown employees; ii) where BMJ has agreed a CC-BY licence shall apply, and/or iii) in accordance with the terms applicable for US Federal Government officers or employees acting as part of their official duties; on a worldwide, perpetual, irrevocable, royalty-free basis to BMJ Publishing Group Ltd ("BMJ") its licensees and where the relevant Journal is co-owned by BMJ to the co-owners of the Journal, to publish the Work in this journal and any other BMJ products and to exploit all rights, as set out in our licence.

The Submitting Author accepts and understands that any supply made under these terms is made by BMJ to the Submitting Author unless you are acting as an employee on behalf of your employer or a postgraduate student of an affiliated institution which is paying any applicable article publishing charge ("APC") for Open Access articles. Where the Submitting Author wishes to make the Work available on an Open Access basis (and intends to pay the relevant APC), the terms of reuse of such Open Access shall be governed by a Creative Commons licence – details of these licences and which Creative Commons licence will apply to this Work are set out in our licence referred to above.

Other than as permitted in any relevant BMJ Author's Self Archiving Policies, I confirm this Work has not been accepted for publication elsewhere, is not being considered for publication elsewhere and does not duplicate material already published. I confirm all authors consent to publication of this Work and authorise the granting of this licence.

For peer review only

Inhaled Ciclesonide in Adults Hospitalized with Covid-19: a Randomized Controlled Open-label Trial (HALT Covid-19)

Daniel Brodin[1*], MD; Per Tornhammar[2,3*], MD; Peter Ueda[4*¤], MD, PhD; Anders Krifors[5,6] MD; Eli Westerlund[7], MD, PhD; Simon Athlin[8], MD, PhD; Sandra Wojt[9], MD; Olof Elvstam[10], MD, PhD; Anca Neumann[1], MD; Arsim Elshani[11], MD; Julia Giesecke[3] MD; Jens Edvardsson-Källkvist[3], MD; Sayam Bunpuckdee[12], MD student; Christian Unge[9], MD, PhD; Martin Larsson[13,14], MD, PhD; Björn Johansson[15], MD; Johan Ljungberg[15], MD; Jonas Lindell[16], MD; Johan Hansson[17], MD, PhD; Ola Blennow[1,3,18#¤], MD, PhD, Daniel P Andersson[3,13#] MD, PhD

*Equal contributions

#Equal contributions

¤Corresponding author

Correspondence

Ola Blennow MD, PhD Address: Department of Infectious Diseases, Karolinska University Hospital, Huddinge, 14141 Stockholm Sweden

Email: ola.blennow@regionstockholm.se

Phone: +46739813617

Affiliations:

[1] Department of Internal Medicine, Capio St Göran´s Hospital, Stockholm, Sweden

[2] Functional Area of Emergency Medicine, Karolinska University Hospital Huddinge, Stockholm, Sweden

For peer review only - http://bmjopen.bmj.com/site/about/guidelines.xhtml

[3] Department of Medicine (H7), Karolinska Institutet, Karolinska University Hospital, Huddinge, 141 86 Stockholm, Sweden

[4] Clinical Epidemiology Division, Department of Medicine Solna, Karolinska Institutet, Stockholm, Sweden

[5] Department of Physiology and Pharmacology, Karolinska Institutet, Stockholm, Sweden.

[6] Centre for Clinical Research Västmanland, Uppsala University, Västmanland County Hospital, Västerås, Sweden.

[7] Department of Clinical Sciences, Karolinska Institutet, Danderyd Hospital, Stockholm, Sweden

[8] School of Medical Sciences, Faculty of Medicine, Örebro University, Örebro, Sweden

[9] Department of Internal Medicine, Danderyd Hospital, Stockholm, Sweden

[10] Department of Infectious Diseases, Växjö Central Hospital, Växjö, Sweden

[11] Department in Geriatric Medicine at Karlskoga Hospital, Karlskoga, Sweden

[12] At the time of the study: Department of Medicine (H7), Karolinska Institutet, Stockholm, Sweden

[13] Department of Endocrinology, Karolinska University Hospital, Stockholm, Sweden

[14] Department of Molecular Medicine and Surgery (K1), Karolinska Institutet, Stockholm, Sweden

[15] Department of Infectious Diseases Halland County Hospital Halmstad, Halmstad, Sweden

[16] Department of Infectious Diseases, Visby Hospital, Visby, Sweden

[17] Department of Infectious Diseases, Östersund Hospital, Östersund, Sweden

For peer review only

For peer review only - http://bmjopen.bmj.com/site/about/guidelines.xhtml

[18] Department of Infectious Diseases, Karolinska University Hospital, Stockholm, Sweden

Word count: 3076

Abstract word count: 250

For peer review only

For peer review only - http://bmjopen.bmj.com/site/about/guidelines.xhtml

Abstract

Objective: To assess the efficacy of inhaled ciclesonide in reducing the duration of oxygen therapy (an indicator of time to clinical improvement) among adults hospitalized with Covid-19.

Design: Multicenter, randomized, controlled, open-label trial.

Setting: 9 hospitals (3 academic hospitals and 6 non-academic hospitals) in Sweden between June 1, 2020, and May 17, 2021.

Participants: Adults hospitalized with Covid-19 and receiving oxygen therapy.

Intervention: Inhaled ciclesonide 320 µg twice daily for 14 days versus standard care.

Main outcome measures: Primary outcome was duration of oxygen therapy, an indicator of time to clinical improvement. Key secondary outcome was a composite of invasive mechanical ventilation/death.

Results: Data from 98 participants were analyzed (48 receiving ciclesonide and 50 receiving standard care; median (IQR) age, 59.5 (49-67) years; 67 (68%) male). Median (IQR) duration of oxygen therapy was 5.5 (3-9) days in the ciclesonide group and 4 (2-7) days in the standard care group (hazard ratio (HR) for termination of oxygen therapy 0.73 (95% CI 0.47-1.11), with the upper 95% CI being compatible with a 10% relative reduction in oxygen therapy duration, corresponding to a <1-day absolute reduction in a post-hoc calculation). Three participants in each group died/received invasive mechanical ventilation (HR 0.90 (95% CI 0.15-5.32)). The trial was discontinued early due to slow enrollment.

For peer review only - http://bmjopen.bmj.com/site/about/guidelines.xhtml

Conclusions: In hospitalized Covid-19 patients receiving oxygen therapy, this trial ruled out, with 0.95 confidence, a treatment effect of ciclesonide corresponding to more than a one-day reduction in duration of oxygen therapy. Ciclesonide is unlikely to improve this outcome meaningfully.

Strengths and limitations of this study

- This was a multicenter, randomized, controlled, open-label trial comparing treatment with the inhaled corticosteroid ciclesonide 320 µg twice daily for 14 days versus standard care.
- Healthcare providers and participants were not blinded to treatment assignment.
- The trial was terminated early due to slow recruitment.

For peer review only

Introduction

Patients with Covid-19 can develop acute respiratory failure that may require invasive mechanical ventilation, associated with high mortality. The unregulated inflammation in the lungs, poor oxygenation and pulmonary infiltrates characterizing severe Covid-19 have been considered as a type of acute respiratory distress syndrome (ARDS).[1, 2]

Prior to the Covid-19 pandemic, studies have indicated that inhaled corticosteroids may reduce the risk of ARDS. In a randomized controlled trial including 61 patients at risk of ARDS, none of the patients assigned to aerosolized budesonide/formoterol vs 7 assigned to placebo developed ARDS,[3] and 6 (20%) and 16 (53%) of the patients, respectively, received mechanical ventilation. In another trial including 60 patients with acute lung injury or ARDS, nebulized budesonide improved oxygenation and peak and plateau airway pressures, and reduced inflammatory markers.[4] Moreover, potentially protective and preventive effects of inhaled corticosteroids for ARDS is supported by animals models of lung injury,[5-8] and *in vitro* studies,[9] and it has been speculated that local administration of the drug in the lung may maximize therapeutic benefits with fewer systemic side effects, as compared with systemic steroids.[3]

Therefore, it could be hypothesized that inhaled corticosteroids may be beneficial for patients with severe Covid-19. The hypothesis is further supported by reports that inhaled corticosteroids reduce the epithelial expression of genes linked to SARS-CoV-2 entry into host cells.[10, 11] Among the inhaled corticosteroids, ciclesonide has been identified as a particularly promising treatment as it can suppress replication of SARS-CoV-2 *in vitro*.[12, 13]

For peer review only - http://bmjopen.bmj.com/site/about/guidelines.xhtml

While previous randomized controlled trials have assessed the effects of inhaled budesonide[14, 15] or ciclesonide[16, 17] in non-hospitalized Covid-19 patients, no study has been performed in hospitalized patients with more severe Covid-19.

This open-label randomized controlled trial investigated the effects of inhaled ciclesonide, compared to standard care, in adult patients hospitalized with Covid-19 and requiring oxygen therapy.

Methods

Study design

The HALT Covid-19 (inHALation of ciclesonide for Treatment of Covid-19) trial was a multicenter, open-label randomized controlled trial to assess the efficacy and safety of inhaled ciclesonide for the treatment of hospitalized patients with Covid-19 receiving oxygen therapy. The trial was conducted at 9 hospitals (3 academic hospitals and 6 non-academic hospitals) in Sweden between June 1, 2020, and May 17, 2021.

All participants provided written informed consent. The study was approved by the Swedish Ethical Review Authority (Ethics committee number 2020-02183) and the Swedish Medical Products Agency (Eudra-CT number 2020-001928-34) and registered at clinicaltrials.gov (NCT04381364).

Protocol changes and rationale

For peer review only

The trial was designed in the beginning of the Covid-19 pandemic. After trial initiation, treatments for, and hospitalization rates of, patients with Covid-19 changed rapidly. Therefore, we made protocol changes (described in detail in the Online Appendix) and the trial was stopped early.

In brief, we increased the number of study centers, removed the upper age limit (≤85 years) for patient inclusion, changed the inclusion criteria from ≤48 hours since hospital admission to ≤48 hours from initiation of oxygen therapy and allowed for patients to be included on the basis of a positive antigen test for SARS-Cov-2. All changes were approved by the Data Monitoring Committee, Ethical Review Authority and the Swedish Medical Products Agency and implemented from December 2020.

In June 2021, 99 patients had been included in the study, a large and increasing proportion of the adult Swedish population had received Covid-19 vaccination and hospitalizations for Covid-19 had dropped substantially. We determined that it was unlikely that the intended sample size would be reached and asked the Data Monitoring Committee to convene for a meeting. Following the recommendation of the Data Monitoring Committee, the study was terminated for futility to meet the targeted enrolment.

Participants

Based on observations from Covid-19 patients treated at the study centers, we expected that 85% of the standard care group would survive and terminate oxygen

For peer review only - http://bmjopen.bmj.com/site/about/guidelines.xhtml

therapy within 30 days (median 8 days). We considered a 25% (2 days) reduction in the duration of oxygen therapy to be a clinically meaningful effect. We estimated that such an effect could be detected with α of 0.05, and 80% power if 446 participants (223 in each group) were enrolled.

Participants were eligible for inclusion if, they (1) were aged ≥18 years, (2) had a polymerase chain reaction confirmed SARS-CoV-2 infection or a positive SARS-CoV-2 antigen test from the upper respiratory tract, (3) were hospitalized at any of the study hospitals and (4) were receiving oxygen therapy, initiated within 48 hours before inclusion. Key exclusion criteria were ongoing treatment with inhaled or oral corticosteroids (previous use was accepted), oxygen therapy with >8 L oxygen/min or >50 % oxygen on nasal high-flow cannula, and ongoing or expected intensive care or palliative care (Online Appendix).

Randomization

Patients were randomized 1:1 in blocks of 8, stratified by sex and hospital to receive ciclesonide or standard care. The randomization sequence was prepared by a statistician not involved in the trial. Treatment allocation was provided through a web-based interface. The participants and the physicians treating them were unblinded to the treatment assignment.

Intervention

The treatment was 320 μg of inhaled ciclesonide (80 μg per actuation, for a total of 4 actuations, or 160 μg per actuation, for a total of 2 actuations) twice daily (total daily dose 640 μg) for 14 days. Ciclesonide was administered using a spacer (L'espace,

For peer review only - http://bmjopen.bmj.com/site/about/guidelines.xhtml

For peer review only

Nordic Infucare, Stockholm Sweden). Participants randomized to ciclesonide received written instructions, including pictures, and practical instructions on how to use the inhalator and spacer; the first dose was taken under supervision. Ciclesonide was then prescribed in the participant's electronic medical record and each given dose during the hospitalization was recorded. Participants discharged before day 14 were instructed to continue the treatment at home for a total treatment duration of 14 days. Participants randomized to standard care did not receive any intervention related to the study. Physicians treating the participants were not given any restrictions concerning treatments during the study period. Participants who had been discharged were contacted by telephone after day 30 for a follow-up interview.

Outcomes

The primary outcome was duration of oxygen therapy (time to termination of oxygen therapy in days) up to 30 days from randomization. Oxygen therapy was defined as terminated on the day after which the patient did not receive oxygen therapy during at least 48 hours, while being alive. This outcome corresponded to clinical improvement for patients receiving oxygen therapy according to the World Health Organization clinical progression scale.[18]

The key secondary outcome was a composite of invasive mechanical ventilation and death up to 30 days after randomization. Other secondary outcomes were each component of the key secondary outcome, admission to an intensive care unit, discharge from the hospital and dyspnea in daily living at 30-35 days after randomization as evaluated by the mMRC (Modified Medical Research Council)

For peer review only - http://bmjopen.bmj.com/site/about/guidelines.xhtml

dyspnea scale. The scale ranges from 0 to 4 with a higher score indicating more severe dyspnea.[19, 20]

Data on serious adverse events[21] were collected by review of electronic medical records. Information about non-serious adverse events associated with ciclesonide use (dryness of mouth, nausea and oral candidiasis) was reported using a paper-based reporting form which was filled in by the treating physician. Information about non-serious adverse events occurring after hospital discharge was collected during the follow-up interview.

Data collection

Patient characteristics at baseline (comorbidities, comedications, clinical parameters) and study outcomes were obtained from electronic medical records. Investigators contacted participants after day 30 after randomization to ask them about non-serious adverse events and dyspnea in daily living (study outcome) at day 30-35 after randomization.

Statistical analysis

According to the pre-specified analysis plan in the study protocol, the analyses were performed by an investigator who had not been involved in the enrolment of participants and was blinded to treatment assignment. An intention-to-treat population was used. In the analysis of the duration of oxygen therapy, participants were followed from randomization to termination of oxygen therapy, death, or 30 days after randomization. Kaplan Meier cumulative incidence curves were generated to

For peer review only - http://bmjopen.bmj.com/site/about/guidelines.xhtml

illustrate the cumulative incidence of termination of oxygen therapy in the ciclesonide and standard care groups. A Cox proportional hazard regression model, adjusted for study hospital (Appendix Table 1), age (continuous variable) and sex was used to estimate hazard ratios (HR) with 95% CI for time-to-event outcomes. Proportions and the absolute risk difference with 95% CI were presented for binary outcomes. Subgroup analyses were performed for the primary outcome by sex, age (<70 years and ≥70 years) and duration of Covid-19 symptoms (<10 days and ≥10 days). In a per-protocol analysis of the primary outcome, participants assigned to ciclesonide were censored at the time of discontinuing treatment. The median mMRC score was compared using the Kruskal-Wallis test. A logistic regression model adjusted for study hospital, age and sex was used to compare the likelihood of reporting a mMRC score of 0 (dyspnea only with strenuous exercise).

95% CIs of ratios not including 1 and 95% CIs for absolute risk differences not including 0 were considered statistically significant. Secondary outcome analyses and subgroup analyses were considered hypothesis-generating and no adjustment for multiple testing was made. Analyses were performed using Stata version 16.1 (StataCorp).

Patient and Public involvement

No patients were involved in setting the research question, nor in the design, conduct, or interpretation of the study.

For peer review only - http://bmjopen.bmj.com/site/about/guidelines.xhtml

Results

Of the 99 participants who underwent randomization 48 were assigned to receive ciclesonide and 51 to standard care (Figure 1). One participant in the standard care group withdrew consent and was excluded from the analysis. Ninety-eight patients (48 in the ciclesonide group and 50 in the standard care group) were included in the final analysis. All participants assigned to ciclesonide received the treatment at least once. None of the participants were lost to follow-up. The median age of participants was 59.5 (IQR 49, 67) years, 68% were men and the median duration of symptoms was 9 (IQR 8, 11) days. There were no relevant between-group differences in demographic characteristics, laboratory test results or comorbidities at enrollment (Table 1).

Table 1 Demographic and clinical characteristics of participants at study enrolment.

	**Total** (n=98)	**Ciclesonide** (n=48)	**Standard care** (n=50)
Age, median (IQR)	59.5 (49, 67)	61 (49, 67)	59 (49, 67)
Age ≥70 years, n (%)	78 (80)	37 (77)	41 (82)
Men, n (%)	67 (68)	34 (71)	33 (66)
Days since symptom onset, median (IQR)	9 (8, 11)	9 (7.5, 11.5)	10 (8, 11)
Days since symptom onset: <10 days, n (%)	51 (52)	27 (56)	24 (48)
Body mass index in kg/m^2, median (IQR)	29.7 (25.6, 34.0)	28.7 (25.4, 34.0)	30.6 (26.8, 34.3)
Oxygen flow of oxygen therapy in L/min, median (IQR)	2 (1, 3)	2 (1, 3)	2 (1, 2)
Respiratory rate per minute, median (IQR)	20 (18, 24)	20 (19, 25)	20 (18, 23)
C-reactive protein in mg/L, median (IQR)	100 (56, 142)	103 (62, 164)	91.5 (45.5, 124.5)
White cell count in x 10^9/L, median IQR	5.7 (4.5, 7.0)	5.3 (4.3, 6.9)	6.1 (4.9, 7.0)

For peer review only - http://bmjopen.bmj.com/site/about/guidelines.xhtml

eGFR in mL/min/1.73m^2, median (IQR)	83 (70.5, 90)	81.5 (70, 90)	87 (73, 90)
**Coexisting conditions, n (%)**			
Diabetes mellitus	18 (18)	8 (17)	10 (20)
Hypertension[a]	45 (46)	22 (46)	23 (46)
Hyperlipidemia[b]	27 (28)	12 (25)	15 (30)
Chronic obstructive lung disease	3 (3)	1 (2)	2 (4)
Asthma	8 (8)	6 (13)	2 (4)
Current smoker	12 (12)	6 (13)	6 (12)
Ischemic heart disease	8 (8)	2 (4)	6 (12)
Heart failure	3 (3)	2 (4)	1 (2)
Atrial fibrillation	5 (5)	3 (6)	2 (4)
Cancer	10 (10)	5 (10)	5 (10)
Chronic kidney disease	9 (9)	5 (10)	4 (8)

[a] Diagnosis of hypertension or use of antihypertensive drugs

[b] Diagnosis of hyperlipidemia or use of lipid lowering therapy

Missing values were: n=1 for days since symptom onset, n=20 for body mass index, n=1 for oxygen flow of oxygen therapy, n=1 for body temperature, n=1 for heart rate, n=3 for respiratory rate, n=3 for C-reactive protein, n=7 for white cell count and n=22 for eGFR.

eGFR: estimated glomerular filtration rate

The results of primary and secondary outcome analyses are presented in Table 2. Kaplan-Meier estimates of the median duration of oxygen therapy were 5.5 (IQR 3, 9) days in the ciclesonide group and 4 (2, 7) days in the standard care group. (Figure 2). The HR for termination of oxygen therapy during 30 days following randomization, used to compare ciclesonide vs standard care, showed that ciclesonide treatment was not statistically significantly associated with the duration of oxygen therapy (0.73 (95% CI 0.47 to 1.11)). The upper limit of the 95% CI was compatible with a maximum relative reduction[22] in duration of oxygen therapy of 10% (1-1/1.11) with

For peer review only - http://bmjopen.bmj.com/site/about/guidelines.xhtml

For peer review only

ciclesonide, which in a post-hoc calculation described in the Online Appendix, corresponded to a <1 day absolute reduction. In the per-protocol analysis, the HR for termination of oxygen therapy during 30 days following randomization was 0.79 (95% CI 0.51 to 1.23) (Table 2).

Table 2 Outcomes. All outcomes are recorded during 30 days following randomization unless otherwise indicated.

	Ciclesonide	Standard care	Difference[a]
**Primary outcome**			
Duration of oxygen therapy, median (IQR) days	5.5 (3, 9)	4 (2, 7)	0.73 (0.47 to 1.11)
**Key secondary outcome**			
Death or invasive mechanical ventilation, n (%)	3 (6)	3 (6)	0 (-9 to 10)
Time to death or invasive mechanical ventilation, median (IQR) days	2 (2, 10)	4 (2, 7)	0.90 (0.15 to 5.32)
**Secondary outcomes**			
Death, n (%)	2 (4)	1 (2)	-
Invasive mechanical ventilation, n (%)	1 (2)	3 (6)	-
Admission to an intensive care unit, n (%)	4 (8)	4 (8)	-
mMRC dyspnea scale score at day 30-35, median (IQR)[b]	3 (2, 4)	3 (2, 4)	0.97
mMRC dyspnea scale score 0 at day 30-35, n (%)[b]	4 (9)	7 (15)	0.48 (0.11 to 2.04)
**Subgroup analyses**[c]			
Sex: Men			
Duration of oxygen therapy, median (IQR) days	5.5 (3, 9)	5 (2, 7)	0.61 (0.36 to 1.05)
Sex: Women			
Duration of oxygen therapy, median (IQR) days	5.5 (2, 7)	4 (2, 8)	0.91 (0.41 to 2.01)
Age group: <70 years			
Duration of oxygen therapy, median (IQR) days	5 (3, 7)	4 (2, 7)	0.77 (0.48 to 1.23)
Age group: ≥70 years			
Duration of oxygen therapy, median (IQR) days	9 (5, 10)	6 (5, 8)	0.37 (p=0.266[d])
Days since symptom onset: <10 days			

For peer review only

For peer review only - http://bmjopen.bmj.com/site/about/guidelines.xhtml

Duration of oxygen therapy, median (IQR) days	7 (3, 10)	4 (2, 5)	0.54 (0.28 to 1.03)
Days since symptom onset: ≥10 days			
Duration of oxygen therapy, median (IQR) days	5 (3, 6)	5 (3, 8)	0.97 (0.48 to 1.94)
**Per protocol analysis**[e]			
Duration of oxygen therapy, median (IQR) days	5 (3, 9)	4 (2, 7)	0.79 (0.51 to 1.23)

[a.] Differences are expressed as hazard ratios (95% CI) estimated using a Cox proportional hazards model for time to event outcomes and as absolute risk difference (95% CI) in percent for outcomes of absolute risk. The comparison of the mMRC dyspnea score was done using the Kruskal-Wallis test and the difference is expressed as a p-value. The comparison of the likelihood of reporting a mMRC score of 0 was done using a logistic regression model and the difference is expressed as an odds ratio (95% CI). Statistical testing for differences in proportions and time-to-event analyses were not performed for the secondary outcome events, including death, invasive mechanical ventilation, and admission to an intensive care unit due to few events.

[b.] Not including 1 participant in the standard care group and 2 participants in the ciclesonide group who died within 30 days of randomization and 1 participant in the standard care group and 1 participant in the ciclesonide group with missing data on this outcome.

[c.] The subgroup analyses included n=33 in the standard care group and n=34 in the ciclesonide group for men, n=17 in the standard care group and n=14 in the ciclesonide group for women, n=41 in the standard care group and n=37 in the ciclesonide group for those aged <70 years, n=9 in the standard care group and n=11 in the ciclesonide group for those aged ≥70 years, n=24 in the standard care group and n=27 in the ciclesonide group for those with <10 days since symptom onset , and n=25 in the standard care group and n=21 in the ciclesonide group for those with ≥10 days since symptom onset. 1 participant had missing data on days since symptom onset and was not included in the subgroup analysis.

[d.] 95% CI were not calculated due to low sample size. The p-value is calculated using the Mann-Whitney U Test for duration of oxygen therapy adter exclusion 1 patients in the standard care group and 2 patient in the ciclesonide group who died or received invasive mechanical intervention during 30 days after randomization. The use of the Mann-Whitney U Test was a post-hoc decision and the analysis could not be adjusted for age, sex and study hospital.

[e.] In the per-protocol analysis for duration of oxygen therapy, patients assigned to ciclesonide were censored at the time of discontinuing treatment.

For peer review only - http://bmjopen.bmj.com/site/about/guidelines.xhtml

In total, 3 (6%) participants assigned to ciclesonide and 3 (6%) participants assigned to standard care experienced the key secondary outcome of mechanical invasive ventilation or death (absolute difference 0% (95% CI -10 to 9%; HR 0.90 (95% CI 0.15 to 5.32)). Median mMRC dyspnea score at 30-35 days after randomization was 3 (IQR 2, 4) in both groups (p-value for difference 0.97) (Table 2).

There were no statistically significant differences between those assigned to ciclesonide vs standard care in the primary outcome in any of the subgroup analyses by sex, age (<70 years and ≥70 years) and days since symptom onset (<10 days and ≥10 days) (Table 2).

There were no apparent differences between the groups in treatments that participants received after randomization (Table 3); 26 (54%) of the participants assigned to ciclesonide and 22 (44%) of the participants in the standard care group received treatment with systemic corticosteroids after randomization.

Few serious adverse clinical events occurred during the study. The most frequently reported adverse event was dry mouth (7 (15%) participants in the ciclesonide group and 11 (22%) participants in the standard care group). Two participants assigned to ciclesonide and 0 in the placebo group reported that they experienced oral candidiasis (Table 3).

Table 3 Participants' treatments and adverse clinical events through day 30 after randomization.

For peer review only - http://bmjopen.bmj.com/site/about/guidelines.xhtml

	Ciclesonide (n=48)	Standard care (n=50)
**Received treatment, n (%)**		
Systemic corticosteroids	26 (54)	22 (44)
Remdesivir	4 (8)	5 (10)
Low-molecular-weight heparin	45 (94)	45 (90)
Oral anticoagulants	32 (67)	30 (60)
Vasopressors	4 (8)	3 (6)
Non-invasive mechanical ventilation	8 (17)	7 (14)
**Serious clinical events, n (%)**		
Renal failure	2 (4)	3 (6)
Cardiac arrest	1 (2)	0 (0)
New onset atrial fibrillation	0 (0)	1 (2)
Pulmonary embolism	4 (8)	2 (4)
Other thromboembolic events	0 (0)	1 (2)
Sepsis	3 (6)	2 (4)
Other serious event	1 (2)	0 (0)
**Non-serious adverse events, n (%)**		
Nausea	6 (13)	8 (16)
Dry mouth	7 (15)	11 (22)
Oral candidiasis	2 (4)	0 (0)
Other non-serious adverse event	3 (6)	1 (2)

Some pre-specified analyses were not performed due to small sample size or low number of events. These included statistical testing of differences in proportions and time-to-event analyses for non-key secondary outcomes, including death, invasive mechanical ventilation, and admission to an intensive care unit; the secondary outcome analyses of discharge from hospital; subgroup analyses for the secondary

For peer review only - http://bmjopen.bmj.com/site/about/guidelines.xhtml

outcomes, and the primary outcome analysis after exclusion of participants who received invasive mechanical ventilation or died.

Discussion

In this randomized open-label, controlled trial, including 98 hospitalized Covid-19 patients with ongoing oxygen therapy, treatment with inhaled ciclesonide did not result in a statistically significant reduction in the duration of oxygen therapy, used as a measure of time to clinical improvement. The trial ruled out, with 0.95 confidence, treatments effects of ciclesonide corresponding to more than a one-day reduction in duration of oxygen therapy.

While previous randomized controlled trials have assessed effects of inhaled corticosteroids, including budesonide[14, 15] and ciclesonide[16, 17], in non-hospitalized patients with Covid-19, this is the first trial that includes hospitalized patients with more severe forms of the disease. In contrast to our hypothesis, the median duration of oxygen therapy was nominally longer among patients assigned to ciclesonide vs standard care (5.5 vs 4 days; HR for termination of oxygen therapy 0.73 (95% CI 0.47 to 1.11)). As such, the 95% CI indicates that,[22] even in the best case, ciclesonide may reduce the duration of oxygen therapy with only 10% (1-1/1.11; less than 1 day in our study) while it may in the worst case result in an over 2-fold increase. Thus, the results of this trial indicate that ciclesonide is unlikely to provide a clinically meaningful beneficial effect on the duration of oxygen therapy in hospitalized Covid-19 patients receiving oxygen therapy.

For peer review only - http://bmjopen.bmj.com/site/about/guidelines.xhtml

To date, 2 randomized controlled trials of ciclesonide in non-hospitalized patients with Covid-19 have been presented. In the CONTAIN study,[16] which was terminated early due to slow recruitment, 215 non-hospitalized patients with a median of 3 days symptom duration were randomized to combination treatment with intranasal and inhaled ciclesonide or placebo. No statistically significant difference between the groups was observed for the primary endpoint, resolution of respiratory symptoms at day 7 after randomization, which was reached by 40% of the patients in the treatment group vs 35% in the placebo group (adjusted risk difference of 5.5% (95% CI -7.8% to 18.8%).[16] Six (6%) patients assigned to ciclesonide vs 3 (3%) in the placebo group were hospitalized within 14 days; none died. In another clinical trial of ciclesonide, including 400 non-hospitalized patients with Covid-19,[17] randomization to ciclesonid vs placebo did not result in a reduced time to alleviation of all Covid-19 related symptoms. However, in secondary outcome analyses, patients assigned to ciclesonide had fewer emergency department visits or hospital admissions for reasons related to COVID-19 (odds ratio, 0.18, 95% CI, 0.04 to 0.85).

In addition, 2 randomized clinical trials of the inhaled corticosteroid budesonide in non-hospitalized patients with Covid-19 have been presented. The STOIC trial was an open-label trial comparing inhaled budesonide vs standard care in 146 Covid-19 patients with mild symptoms.[14] Compared to standard care, budesonide treatment led to a statistically significant reduction in Covid-19-related emergency department assessment and hospitalization (difference in proportions 0.123 (95% CI 0.043 to 0.218).[14] Furthermore, budesonide treatment was associated with 1 day shorter time to clinical recovery. The PRINCIPLE trial was another open-label trial that included 4700 primary care patients at high risk of developing severe Covid-19 (1073

For peer review only - http://bmjopen.bmj.com/site/about/guidelines.xhtml

randomized to budesonide treatment; 1988 to standard care; 1639 to other treatments).[15] Compared to standard care, randomization to budesonide led to a shorter time to self-reported recovery (difference 2.94 days (95% Bayesian credible interval 1.19 to 5.12) and a reduced likelihood of hospital admission or death, although the results for the latter outcomes did not meet the superiority threshold.

Taken together, the previous studies indicate that inhaled corticosteroids might be useful for preventing deterioration of Covid-19 in non-hospitalized patients with mild symptoms. It is possible that the low likelihood of benefit associated with ciclesonide treatment observed in our study reflects the more severe pulmonary inflammation in our study population, as indicated by the need for hospitalization with oxygen therapy and a median symptom duration of 9 days: at such stages of disease progression, it could be speculated that pulmonary administration of corticosteroids may not suffice to confer benefit and that systemic treatment is needed. Accordingly, in the Recovery trial of hospitalized Covid-19 patients,[23] dexamethasone treatment reduced risk of death and the time to discharge from hospital, with these benefits primarily being observed among patients receiving oxygen therapy or invasive mechanical ventilation at baseline.

Similar to other clinical trials including patients with Covid-19,[15, 23, 24] we used a pragmatic, open-label design. With this design, we intended to assess the effect of adding ciclesonide to standard care, rather than to examine the effect of ciclesonide compared to placebo. The research question that our study aimed to answer was “what is the effect of using ciclesonide as an addition to standard care as compared with standard care alone?” While this is a research question of relevance to clinical

For peer review only - http://bmjopen.bmj.com/site/about/guidelines.xhtml

decision-making, the open-label design and the possible expectations of effect among both patients[25] and physicians might have affected the outcomes in our study, including when to terminate oxygen therapy. Another limitation of our study is that we were unable to recruit the intended number of patients due to the substantial decrease in hospitalized Covid-19 patients in Sweden during 2021. Importantly, the study could not provide much information regarding the key secondary outcome of death or invasive mechanical intervention. Further research in hospitalized Covid-19 patients is needed to determine the potential effect of ciclesonide treatment on these outcomes. Moreover, it is a possibility that effects of ciclesonide differ as compared to other inhaled corticosteroids (e.g., budesonide). Patients were instructed to use ciclesonide without a spacer after discharge from the hospital; this may have affected drug delivery. Finally, results from the Recovery Trial were released 5 weeks after the initiation of our study and around half of the patients in both the ciclesonide group and the control group received systemic corticosteroids after randomization. Further studies would be needed to assess the comparative effectiveness and safety of ciclesonide vs systemic corticosteroids.

Conclusions

In this open-label randomized controlled trial in patients hospitalized with Covid-19 and receiving oxygen therapy, the findings indicated that treatment with ciclesonide vs standard care is unlikely to result in a clinically meaningful reduction in the duration of oxygen therapy.

Acknowledgements

For peer review only - http://bmjopen.bmj.com/site/about/guidelines.xhtml

We would like to thank the following individuals that did not qualify for authorship but contributed to the study: Dr Oscar Bakhouch (Skaraborg Hospital), Dr Eva-Marie Boman and Dr Anders Lundqvist (Southern Älvsborg Hospital).

Contributorship statement

DB, PU, PT, OB and DPA conceived the study and were responsible for the methods. OB and DPA were responsible for the study conduct. DB, PU, OB and DPA were responsible for the financing. PT validated the data. PU performed the main analysis. DPA, PU and PT wrote the original draft of the manuscript. All authors wrote, reviewed, and edited the manuscript. OB and DPA supervised the study. DB, DPA, PU, PT and OB were responsible for administration of the project. DPA and OB are the guarantors. DB, PT, AK, EW, SA, SW, OE, AN, AE, JG, JEK, BJ, JL, JLI, JH, OB and DPA enrolled participants in the study. The corresponding author attests that all listed authors meet authorship criteria and that no others meeting the criteria have been omitted.

Competing interests

This study received non-financial support from COVIS Pharma (study drug donation). The authors have no conflict of interest to disclose.

Funding/Support

This study was funded by the Swedish Heart and Lung Foundation (Number 20200421), The Axel and Margaret Ax:son Johnson Foundation (N/A), CIMED (N/A), and Strategic Research Program at Karolinska Institutet (Number 961507), the

For peer review only - http://bmjopen.bmj.com/site/about/guidelines.xhtml

Stockholm County Council (Number: 954970, 963296, 962029), and the Västmanland County Council (Grant nr LTV-938409). PU was supported by grants from the Strategic Research Program in Epidemiology at Karolinska Institutet (N/A), and a Faculty Funded Career Position at Karolinska Institutet (N/A).

The funders had no role in the study design, conduct, collection, management, analysis, interpretation of data, writing or reviewing the manuscript or decision to submit the manuscript for publication. The study drug was donated by COVIS Pharma but COVIS pharma did not participate in any other part of the study.

Data sharing statement

Data are available upon resonable request

Ethics Approval

All participants provided written informed consent. The study was approved by the Swedish Ethical Review Authority (Ethics committee number 2020-02183) and the Swedish Medical Products Agency (Eudra-CT number 2020-001928-34) and registered at clinicaltrials.gov (NCT04381364).

References

1. Gibson PG, Qin L, Puah SH. COVID-19 acute respiratory distress syndrome (ARDS): clinical features and differences from typical pre-COVID-19 ARDS. Med J Aust **2020**; 213:54-6 e1.

For peer review only

For peer review only - http://bmjopen.bmj.com/site/about/guidelines.xhtml

2. Bos LDJ. COVID-19-related Acute Respiratory Distress Syndrome: Not So Atypical. Am J Respir Crit Care Med **2020**; 202:622-4.

3. Festic E, Carr GE, Cartin-Ceba R, et al. Randomized Clinical Trial of a Combination of an Inhaled Corticosteroid and Beta Agonist in Patients at Risk of Developing the Acute Respiratory Distress Syndrome. Crit Care Med **2017**; 45:798-805.

4. Mohamed HS, Meguid MM. Effect of nebulized budesonide on respiratory mechanics and oxygenation in acute lung injury/acute respiratory distress syndrome: Randomized controlled study. Saudi J Anaesth **2017**; 11:9-14.

5. Jansson AH, Eriksson C, Wang X. Effects of budesonide and N-acetylcysteine on acute lung hyperinflation, inflammation and injury in rats. Vascul Pharmacol **2005**; 43:101-11.

6. Forsgren PE, Modig JA, Dahlback CM, Axelsson BI. Prophylactic treatment with an aerosolized corticosteroid liposome in a porcine model of early ARDS induced by endotoxaemia. Acta Chir Scand **1990**; 156:423-31.

7. Wang J, Zhang L, Walther SM. Administration of aerosolized terbutaline and budesonide reduces chlorine gas-induced acute lung injury. J Trauma **2004**; 56:850-62.

8. Walther S, Jansson I, Berg S, Lennquist S. Pulmonary granulocyte accumulation is reduced by nebulized corticosteroid in septic pigs. Acta Anaesthesiol Scand **1992**; 36:651-5.

For peer review only

9. Yamaya M, Nishimura H, Deng X, et al. Inhibitory effects of glycopyrronium, formoterol, and budesonide on coronavirus HCoV-229E replication and cytokine production by primary cultures of human nasal and tracheal epithelial cells. Respir Investig **2020**; 58:155-68.

10. Peters MC, Sajuthi S, Deford P, et al. COVID-19-related Genes in Sputum Cells in Asthma. Relationship to Demographic Features and Corticosteroids. Am J Respir Crit Care Med **2020**; 202:83-90.

11. Milne S, Li X, Yang CX, et al. Inhaled corticosteroids downregulate SARS-CoV-2-related genes in COPD: results from a randomised controlled trial. Eur Respir J **2021**; 58.

12. Jeon S, Ko M, Lee J, et al. Identification of Antiviral Drug Candidates against SARS-CoV-2 from FDA-Approved Drugs. Antimicrob Agents Chemother **2020**; 64.

13. Kimura H, Kurusu H, Sada M, et al. Molecular pharmacology of ciclesonide against SARS-CoV-2. J Allergy Clin Immunol **2020**; 146:330-1.

14. Ramakrishnan S, Nicolau DV, Jr., Langford B, et al. Inhaled budesonide in the treatment of early COVID-19 (STOIC): a phase 2, open-label, randomised controlled trial. Lancet Respir Med **2021**; 9:763-72.

15. Yu LM, Bafadhel M, Dorward J, et al. Inhaled budesonide for COVID-19 in people at high risk of complications in the

For peer review only

community in the UK (PRINCIPLE): a randomised, controlled, open-label, adaptive platform trial. Lancet **2021**; 398:843-55.

16. Ezer N, Belga S, Daneman N, et al. Inhaled and intranasal ciclesonide for the treatment of covid-19 in adult outpatients: CONTAIN phase II randomised controlled trial. BMJ **2021**; 375:e068060.

17. Clemency BM, Varughese R, Gonzalez-Rojas Y, et al. Efficacy of Inhaled Ciclesonide for Outpatient Treatment of Adolescents and Adults With Symptomatic COVID-19: A Randomized Clinical Trial. JAMA Intern Med **2021**.

18. Characterisation WHOWGotC, Management of C-i. A minimal common outcome measure set for COVID-19 clinical research. Lancet Infect Dis **2020**; 20:e192-e7.

19. Mahler DA, Wells CK. Evaluation of clinical methods for rating dyspnea. Chest **1988**; 93:580-6.

20. Bestall JC, Paul EA, Garrod R, Garnham R, Jones PW, Wedzicha JA. Usefulness of the Medical Research Council (MRC) dyspnoea scale as a measure of disability in patients with chronic obstructive pulmonary disease. Thorax **1999**; 54:581-6.

21. Directive 2001/20/EC of the European Parliament and of the Council of 4 April 2001 on the approximation of the laws, regulations and administrative provisions of the member states relating to the implementation of good clinical practice in the

For peer review only

conduct of clinical trials on medicinal products for human use. Med Etika Bioet **2002**; 9:12-9.

22. Hawkins AT, Samuels LR. Use of Confidence Intervals in Interpreting Nonstatistically Significant Results. JAMA **2021**; 326:2068-9.

23. Group RC, Horby P, Lim WS, et al. Dexamethasone in Hospitalized Patients with Covid-19. N Engl J Med **2021**; 384:693-704.

24. Investigators R-C, Gordon AC, Mouncey PR, et al. Interleukin-6 Receptor Antagonists in Critically Ill Patients with Covid-19. N Engl J Med **2021**; 384:1491-502.

25. Wechsler ME, Kelley JM, Boyd IO, et al. Active albuterol or placebo, sham acupuncture, or no intervention in asthma. N Engl J Med **2011**; 365:119-26.

For peer review only

Figure 1 Flow diagram for study participants.

Figure 2 Time to termination of oxygen therapy during 30 days after randomization.

For peer review only

For peer review only - http://bmjopen.bmj.com/site/about/guidelines.xhtml

Figure 1

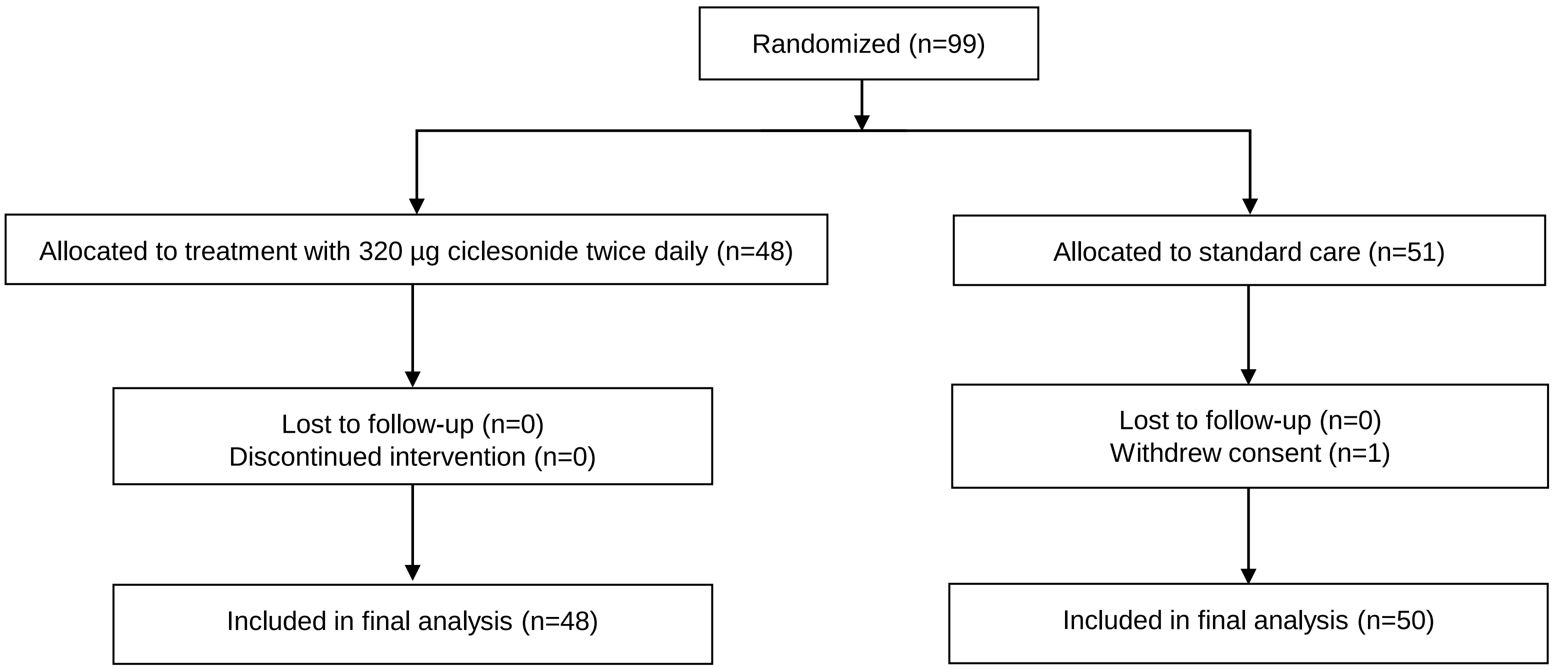
Randomized (n=99)
Allocated to treatment with 320 µg ciclesonide twice daily (n=48)
Allocated to standard care (n=51)
Lost to follow-up (n=0)
Discontinued intervention (n=0)
Lost to follow-up (n=0)
Withdrew consent (n=1)
Included in final analysis (n=48)
Included in final analysis (n=50)

Figure 2 Time to termination of oxygen therapy.

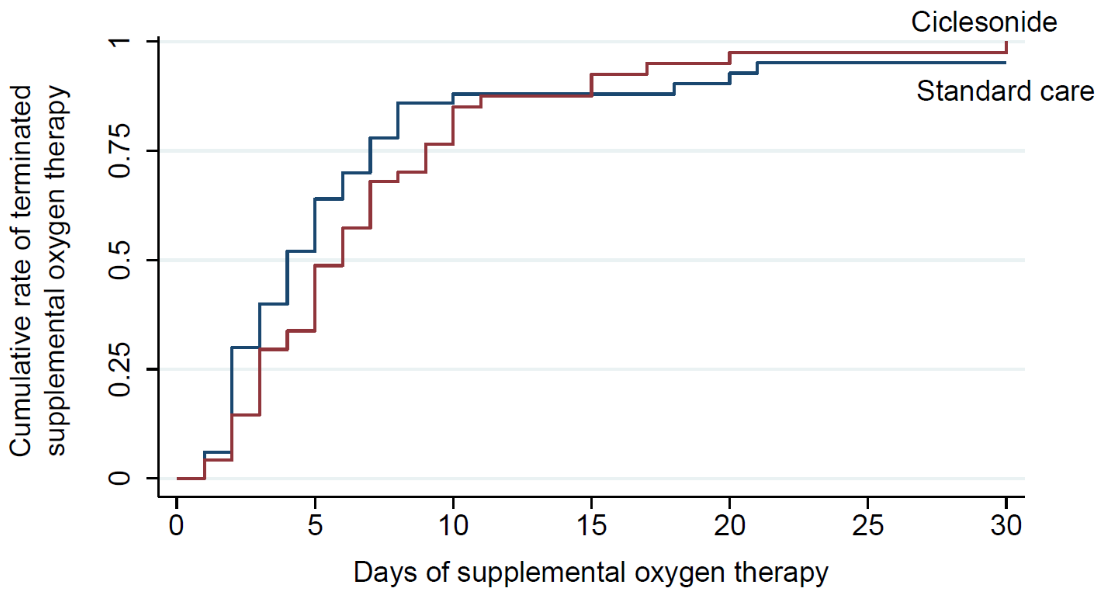

No at risk

Standard care	50	24	7	6	4	2	2
Ciclesonide	48	31	11	5	2	1	1

ew only

ONLINE APPENDIX

Brodin D, Tornhammar P, Ueda P, Krifors A, Westerlund E, Athlin S, Wojt S, Elvstam O, Neumann A, Elshani A, Giesecke J, Edvardsson J, Bunpuckdee S, Unge C, Larsson M, Johansson B, Ljungberg J, Lindell J, Hansson J, Blennow O, Andersson DP. Inhaled Ciclesonide in Adults Hospitalized with Covid-19: a Randomized Controlled Open-label Trial (HALT Covid-19).

For peer review only

Protocol changes and rationale

The trial was designed in the beginning of the covid-19 pandemic when data from randomized clinical trials of Covid-19 treatment were scarce. After trial initiation, treatments for patients with Covid-19 and hospitalization rates of such patients changed rapidly. Therefore, we made changes to the protocol and the trial was stopped early.

5 weeks after the start of patient inclusion in our study, in July 2020, the Recovery Collaborative group presented preliminary data[1] showing protective effects of dexamethasone treatment in patients hospitalized for covid-19; a subgroup analysis of this study indicated that the effect was driven by patients receiving invasive mechanical ventilation or oxygen therapy. These data, in combination with local experience from treating patients with Covid-19,[2] led to most patients receiving oxygen therapy with ≥ 4 L oxygen/min at the study hospitals being treated with systemic corticosteroids. As use of systemic corticosteroids was an exclusion criterion, the change in practice made a large proportion of the Covid-19 patients ineligible for participation.

Initially the trial was conducted at 4 hospitals. To increase the inclusion rate, 9 additional hospitals were included as study sites, although only 5 of them ended up recruiting patients to the study. We also removed the previous upper age limit of 85 years for inclusion and allowed for inclusion of patients based on a positive antigen test for SARS-CoV-2. Moreover, because some patients may start receiving oxygen therapy before hospital admission (e.g., at nursing homes before being transported

For peer review only

to the hospital) or a period after hospital admission (e.g., if the patient's condition deteriorated) and we aimed to include patients shortly after initiation of such therapy, we changed the inclusion criteria from hospitalization within 48 hours prior to enrollment to initiation of oxygen therapy no longer than 48 hours prior to enrollment.

All changes were approved by the Data Monitoring Committee, Ethical Review Authority and the Swedish Medical Products Agency and implemented from December 2020.

In June 2021, when 99 patients had been included in the study, a large and increasing proportion of the adult Swedish population had received vaccination for Covid-19. The number of patients hospitalized with Covid-19 had dropped substantially and there were none to only a few Covid-19 patients admitted to the study hospitals per week. We determined that it was unlikely that we would reach the intended sample size and asked the Data Monitoring Committee to convene for a meeting. Following the recommendation of the Data Monitoring Committee, the study was terminated early due to expected futility to meet total enrolment.

Inclusion and exclusion criteria[a]

Participants were eligible for inclusion if, at the time of study inclusion, they (1) were aged ≥18 years, (2) had a polymerase chain reaction confirmed SARS-CoV-2 infection or a positive antigen test for SARS-CoV-2, (3) were hospitalized at any of the study hospitals and (4) were receiving oxygen therapy with not more than 48 hours having passed since initiation of this treatment.

Patients were not eligible for inclusion if they (1) had a history of hypersensitivity to ciclesonide or other substances included in the treatment, (2) received ongoing treatment with inhaled or oral corticosteroids, ketokonazol, itrakonazol, ritonavir or nelfinavir, (3) received >8 L oxygen/min or >50 % oxygen with nasal high-flow therapy, (4) were receiving or under consideration for palliative care or had an expected survival of less than 72 h, (5) were expected to be admitted to an intensive care unit within 48 h, (6) had active or inactive pulmonary tuberculosis, severe liver failure (Child-Pugh C), pulmonary arterial hypertension or fibrosis, cognitive or physical impairment, (7) had insufficient language skills to understand information given about the study, (8) had been included in a clinical trial within 30 days, or (9) were women and pregnant, breastfeeding or did not agree to take highly effective contraceptive measures while receiving treatment plus an additional 7 days.

[a] The presentation of these inclusion and exclusion criteria have been modified for readability as compared with the version presented in the study protocol.

For peer review only

Post-hoc calculation for interpretation of the 95% confidence interval in the primary outcome analysis

The research question that we aimed to assess was whether inhaled ciclesonide, as compared with standard care, could reduce the time to clinical improvement (as indicated by duration of oxygen therapy). While the interpretation of statistically non-significant findings is a recurring and well-known subject of debate in the medical literature, it is generally not recommended to use a binary interpretation based on an arbitrary cut-off for statistical significance[3-6]. This is particularly important in this trial as it was terminated early and thereby underpowered to assess its primary outcome. However, it has been suggested that in trials with statistically non-significant findings, the 95% CIs should be used to rule in or rule out potential effect sizes of the intervention. In this study, we therefore assessed the largest benefit of ciclesonide that was compatible with the confidence interval. Such a benefit was represented by the upper limit of the HR for time to termination of oxygen therapy (a higher HR indicates shorter duration of oxygen therapy for the ciclesonide group), i.e., 1.11. We took the inverse of this HR (1/1.11 = 0.90) to calculate the relative reduction in duration of oxygen therapy that the HR was compatible with (i.e., 1-0.90 = 10% relative reduction). We then multiplied this 10% relative reduction with the absolute duration of oxygen therapy in the standard care group to calculate the corresponding absolute difference in duration of oxygen therapy (10% * 4 days = 0.4 days, which is <1 day). Given the pre-specified minimally clinically important difference of 2 days (which was used for the power calculation of the study), we deemed this best-case difference to be clinically irrelevant.

For peer review only

Appendix table 1 Number of participants included in the final study population by study center.

Study center	*n participants*
Danderyd Hospital	26
Capio S:t Göran Hospital	24
Karolinska University Hospital	21
Västmanland County Hospital	13
Örebro University Hospital[a]	6
Växsjö Central Hospital[a]	3
Halland County Hospital[a]	2
Östersund Hospital[a]	2
Visby Hospital[a]	1

[a] In the analyses adjusted for study center, these hospitals were categorized into one group.

For peer review only

References

1. Group RC, Horby P, Lim WS, et al. Dexamethasone in Hospitalized Patients with Covid-19. *N Engl J Med* 2021;384(8):693-704. doi: 10.1056/NEJMoa2021436 [published Online First: 2020/07/18]
2. Kan B, Ahl M, Blennow O, et al. *Lakartidningen* 2020;117 [published Online First: 2020/10/07]
3. Altman DG, Bland JM. Absence of evidence is not evidence of absence. *BMJ* 1995;311(7003):485. doi: 10.1136/bmj.311.7003.485 [published Online First: 1995/08/19]
4. Harrell F. Statistical Errors in the Medical Literature. Statistical Thinking. https://www.fharrell.com/post/errmed/index.html (accessed Oct 24, 2022). 2017.
5. Hawkins AT, Samuels LR. Use of Confidence Intervals in Interpreting Nonstatistically Significant Results. *JAMA* 2021;326(20):2068-69. doi: 10.1001/jama.2021.16172 [published Online First: 2021/11/24]
6. Greenland S, Senn SJ, Rothman KJ, et al. Statistical tests, P values, confidence intervals, and power: a guide to misinterpretations. *Eur J Epidemiol* 2016;31(4):337-50. doi: 10.1007/s10654-016-0149-3 [published Online First: 2016/05/23]

For peer review only

CONSORT 2010 checklist of information to include when reporting a randomised trial*

Section/Topic	Item No	Checklist item	Reported on page No
**Title and abstract**			
	1a	Identification as a randomised trial in the title	1
	1b	Structured summary of trial design, methods, results, and conclusions (for specific guidance see CONSORT for abstracts)	3
**Introduction**			
Background and objectives	2a	Scientific background and explanation of rationale	5,6
	2b	Specific objectives or hypotheses	5-7
**Methods**			
Trial design	3a	Description of trial design (such as parallel, factorial) including allocation ratio	6-9
	3b	Important changes to methods after trial commencement (such as eligibility criteria), with reasons	6,7,9
Participants	4a	Eligibility criteria for participants	7,8
	4b	Settings and locations where the data were collected	6,7
Interventions	5	The interventions for each group with sufficient details to allow replication, including how and when they were actually administered	6-11
Outcomes	6a	Completely defined pre-specified primary and secondary outcome measures, including how and when they were assessed	9-11
	6b	Any changes to trial outcomes after the trial commenced, with reasons	6,7
Sample size	7a	How sample size was determined	6-9
	7b	When applicable, explanation of any interim analyses and stopping guidelines	6,7
Randomisation:			
Sequence generation	8a	Method used to generate the random allocation sequence	8
	8b	Type of randomisation; details of any restriction (such as blocking and block size)	8
Allocation concealment mechanism	9	Mechanism used to implement the random allocation sequence (such as sequentially numbered containers), describing any steps taken to conceal the sequence until interventions were assigned	8
Implementation	10	Who generated the random allocation sequence, who enrolled participants, and who assigned participants to interventions	8
Blinding	11a	If done, who was blinded after assignment to interventions (for example, participants, care providers, those	8,11

For peer review only - http://bmjopen.bmj.com/site/about/guidelines.xhtml

For peer review only

		assessing outcomes) and how	
	11b	If relevant, description of the similarity of interventions	-
Statistical methods	12a	Statistical methods used to compare groups for primary and secondary outcomes	10,11
	12b	Methods for additional analyses, such as subgroup analyses and adjusted analyses	10,11
**Results**			
Participant flow (a diagram is strongly recommended)	13a	For each group, the numbers of participants who were randomly assigned, received intended treatment, and were analysed for the primary outcome	11-13
	13b	For each group, losses and exclusions after randomisation, together with reasons	11-13
Recruitment	14a	Dates defining the periods of recruitment and follow-up	6,7
	14b	Why the trial ended or was stopped	7
Baseline data	15	A table showing baseline demographic and clinical characteristics for each group	Yes
Numbers analysed	16	For each group, number of participants (denominator) included in each analysis and whether the analysis was by original assigned groups	Yes
Outcomes and estimation	17a	For each primary and secondary outcome, results for each group, and the estimated effect size and its precision (such as 95% confidence interval)	Yes
	17b	For binary outcomes, presentation of both absolute and relative effect sizes is recommended	-
Ancillary analyses	18	Results of any other analyses performed, including subgroup analyses and adjusted analyses, distinguishing pre-specified from exploratory	10,11
Harms	19	All important harms or unintended effects in each group (for specific guidance see CONSORT for harms)	13
**Discussion**			
Limitations	20	Trial limitations, addressing sources of potential bias, imprecision, and, if relevant, multiplicity of analyses	13-17
Generalisability	21	Generalisability (external validity, applicability) of the trial findings	13-17
Interpretation	22	Interpretation consistent with results, balancing benefits and harms, and considering other relevant evidence	13-17
**Other information**			
Registration	23	Registration number and name of trial registry	6
Protocol	24	Where the full trial protocol can be accessed, if available	-
Funding	25	Sources of funding and other support (such as supply of drugs), role of funders	18,19

*We strongly recommend reading this statement in conjunction with the CONSORT 2010 Explanation and Elaboration for important clarifications on all the items. If relevant, we also recommend reading CONSORT extensions for cluster randomised trials, non-inferiority and equivalence trials, non-pharmacological treatments, herbal interventions, and pragmatic trials. Additional extensions are forthcoming: for those and for up to date references relevant to this checklist, see www.consort-statement.org.

For peer review only - http://bmjopen.bmj.com/site/about/guidelines.xhtml

Inhaled Ciclesonide in Adults Hospitalized with Covid-19: a Randomized Controlled Open-label Trial (HALT Covid-19)

Journal:	*BMJ Open*
Manuscript ID	bmjopen-2022-064374.R2
Article Type:	Original research
Date Submitted by the Author:	10-Jan-2023
Complete List of Authors:	Brodin, Daniel; Capio Sankt Görans Sjukhus<br>Tornhammar, Per; Karolinska Institute, Functional Area of Emergency Medicine<br>Ueda, Peter; Karolinska Institutet<br>Krifors, Anders; Karolinska Institutet, Department of Physiology and Pharmacology; Västmanlands sjukhus Västerås<br>Westerlund, Eli; Danderyd University Hospital, Department of Clinical Sciences<br>Athlin, Simon; Örebro University<br>Wojt, Sandra; Danderyd University Hospital, Department of Internal Medicine<br>Elvstam, Olof; Central Hospital Växjö, Department of Infectious Diseases<br>Neumann, Anca; Capio Sankt Görans Sjukhus<br>Elshani, Arsim; Karlskoga Hospital<br>Giesecke, Julia; Karolinska Universitetssjukhuset<br>Edvardsson-Källkvist, Jens; Karolinska University Hospital<br>Bunpuckdee, Sayam; Karolinska Institutet<br>Unge, Christian; Danderyd University Hospital, Department of Internal Medicine<br>Larsson, Martin; Karolinska Institutet Department of Clinical Science and Education Sodersjukhuset<br>Johansson, Björn; Halland County<br>Ljungberg, Johan; Halland County<br>Lindell, Jonas; Visby Hospital, Department of Infectious Diseases<br>Hansson, Johan; Östersund Hospital, Department of Infectious Diseases<br>Blennow, Ola; Karolinska University Hospital, Department of Infectious Diseases<br>Andersson, Daniel Peter; Karolinska University Hospital, Department of Medicine Huddinge H7, Karolinska Institutet; Karolinska Institute, Medicine (H7)
**Primary Subject Heading**:	Infectious diseases
Secondary Subject Heading:	Respiratory medicine
Keywords:	COVID-19, Clinical trials < THERAPEUTICS, Respiratory infections < THORACIC MEDICINE

I, the Submitting Author has the right to grant and does grant on behalf of all authors of the Work (as defined in the below author licence), an exclusive licence and/or a non-exclusive licence for contributions from authors who are: i) UK Crown employees; ii) where BMJ has agreed a CC-BY licence shall apply, and/or iii) in accordance with the terms applicable for US Federal Government officers or employees acting as part of their official duties; on a worldwide, perpetual, irrevocable, royalty-free basis to BMJ Publishing Group Ltd ("BMJ") its licensees and where the relevant Journal is co-owned by BMJ to the co-owners of the Journal, to publish the Work in this journal and any other BMJ products and to exploit all rights, as set out in our licence.

The Submitting Author accepts and understands that any supply made under these terms is made by BMJ to the Submitting Author unless you are acting as an employee on behalf of your employer or a postgraduate student of an affiliated institution which is paying any applicable article publishing charge ("APC") for Open Access articles. Where the Submitting Author wishes to make the Work available on an Open Access basis (and intends to pay the relevant APC), the terms of reuse of such Open Access shall be governed by a Creative Commons licence – details of these licences and which Creative Commons licence will apply to this Work are set out in our licence referred to above.

Other than as permitted in any relevant BMJ Author's Self Archiving Policies, I confirm this Work has not been accepted for publication elsewhere, is not being considered for publication elsewhere and does not duplicate material already published. I confirm all authors consent to publication of this Work and authorise the granting of this licence.

For peer review only

Inhaled Ciclesonide in Adults Hospitalized with Covid-19: a Randomized Controlled Open-label Trial (HALT Covid-19)

Daniel Brodin[1*], MD; Per Tornhammar[2,3*], MD; Peter Ueda[4*¤], MD, PhD; Anders Krifors[5,6] MD; Eli Westerlund[7], MD, PhD; Simon Athlin[8], MD, PhD; Sandra Wojt[9], MD; Olof Elvstam[10], MD, PhD; Anca Neumann[1], MD; Arsim Elshani[11], MD; Julia Giesecke[3] MD; Jens Edvardsson-Källkvist[3], MD; Sayam Bunpuckdee[12], MD student; Christian Unge[9], MD, PhD; Martin Larsson[13,14], MD, PhD; Björn Johansson[15], MD; Johan Ljungberg[15], MD; Jonas Lindell[16], MD; Johan Hansson[17], MD, PhD; Ola Blennow[1,3,18#¤], MD, PhD, Daniel P Andersson[3,13#] MD, PhD

*Equal contributions

#Equal contributions

¤Corresponding author

Correspondence

Ola Blennow MD, PhD Address: Department of Infectious Diseases, Karolinska University Hospital, Huddinge, 14141 Stockholm Sweden

Email: ola.blennow@regionstockholm.se

Phone: +46739813617

Affiliations:

[1] Department of Internal Medicine, Capio St Göran´s Hospital, Stockholm, Sweden

[2] Functional Area of Emergency Medicine, Karolinska University Hospital Huddinge, Stockholm, Sweden

For peer review only - http://bmjopen.bmj.com/site/about/guidelines.xhtml

[3] Department of Medicine (H7), Karolinska Institutet, Karolinska University Hospital, Huddinge, 141 86 Stockholm, Sweden

[4] Clinical Epidemiology Division, Department of Medicine Solna, Karolinska Institutet, Stockholm, Sweden

[5] Department of Physiology and Pharmacology, Karolinska Institutet, Stockholm, Sweden.

[6] Centre for Clinical Research Västmanland, Uppsala University, Västmanland County Hospital, Västerås, Sweden.

[7] Department of Clinical Sciences, Karolinska Institutet, Danderyd Hospital, Stockholm, Sweden

[8] School of Medical Sciences, Faculty of Medicine, Örebro University, Örebro, Sweden

[9] Department of Internal Medicine, Danderyd Hospital, Stockholm, Sweden

[10] Department of Infectious Diseases, Växjö Central Hospital, Växjö, Sweden

[11] Department in Geriatric Medicine at Karlskoga Hospital, Karlskoga, Sweden

[12] At the time of the study: Department of Medicine (H7), Karolinska Institutet, Stockholm, Sweden

[13] Department of Endocrinology, Karolinska University Hospital, Stockholm, Sweden

[14] Department of Molecular Medicine and Surgery (K1), Karolinska Institutet, Stockholm, Sweden

[15] Department of Infectious Diseases Halland County Hospital Halmstad, Halmstad, Sweden

[16] Department of Infectious Diseases, Visby Hospital, Visby, Sweden

[17] Department of Infectious Diseases, Östersund Hospital, Östersund, Sweden

For peer review only

For peer review only - http://bmjopen.bmj.com/site/about/guidelines.xhtml

[18] Department of Infectious Diseases, Karolinska University Hospital, Stockholm, Sweden

Word count: 3286

Abstract word count: 250

For peer review only

For peer review only - http://bmjopen.bmj.com/site/about/guidelines.xhtml

Abstract

Objective: To assess the efficacy of inhaled ciclesonide in reducing the duration of oxygen therapy (an indicator of time to clinical improvement) among adults hospitalized with Covid-19.

Design: Multicenter, randomized, controlled, open-label trial.

Setting: 9 hospitals (3 academic hospitals and 6 non-academic hospitals) in Sweden between June 1, 2020, and May 17, 2021.

Participants: Adults hospitalized with Covid-19 and receiving oxygen therapy.

Intervention: Inhaled ciclesonide 320 µg twice daily for 14 days versus standard care.

Main outcome measures: Primary outcome was duration of oxygen therapy, an indicator of time to clinical improvement. Key secondary outcome was a composite of invasive mechanical ventilation/death.

Results: Data from 98 participants were analyzed (48 receiving ciclesonide and 50 receiving standard care; median (IQR) age, 59.5 (49-67) years; 67 (68%) male). Median (IQR) duration of oxygen therapy was 5.5 (3-9) days in the ciclesonide group and 4 (2-7) days in the standard care group (hazard ratio (HR) for termination of oxygen therapy 0.73 (95% CI 0.47-1.11), with the upper 95% CI being compatible with a 10% relative reduction in oxygen therapy duration, corresponding to a <1-day absolute reduction in a post-hoc calculation). Three participants in each group died/received invasive mechanical ventilation (HR 0.90 (95% CI 0.15-5.32)). The trial was discontinued early due to slow enrollment.

For peer review only - http://bmjopen.bmj.com/site/about/guidelines.xhtml

Conclusions: In hospitalized Covid-19 patients receiving oxygen therapy, this trial ruled out, with 0.95 confidence, a treatment effect of ciclesonide corresponding to more than a one-day reduction in duration of oxygen therapy. Ciclesonide is unlikely to improve this outcome meaningfully.

Strengths and limitations of this study

- This was a multicenter, randomized, controlled, open-label trial comparing treatment with the inhaled corticosteroid ciclesonide 320 µg twice daily for 14 days versus standard care.
- Healthcare providers and participants were not blinded to treatment assignment.
- The trial was terminated early due to slow recruitment.

For peer review only - http://bmjopen.bmj.com/site/about/guidelines.xhtml

Introduction

Patients with Covid-19 can develop acute respiratory failure that may require invasive mechanical ventilation, associated with high mortality. The unregulated inflammation in the lungs, poor oxygenation and pulmonary infiltrates characterizing severe Covid-19 have been considered as a type of acute respiratory distress syndrome (ARDS).[1 2]

Prior to the Covid-19 pandemic, studies have indicated that inhaled corticosteroids may reduce the risk of ARDS. In a randomized controlled trial including 61 patients at risk of ARDS, none of the patients assigned to aerosolized budesonide/formoterol vs 7 assigned to placebo developed ARDS,[3] and 6 (20%) and 16 (53%) of the patients, respectively, received mechanical ventilation. In another trial including 60 patients with acute lung injury or ARDS, nebulized budesonide improved oxygenation and peak and plateau airway pressures, and reduced inflammatory markers.[4] Moreover, potentially protective and preventive effects of inhaled corticosteroids for ARDS is supported by animals models of lung injury,[5-8] and *in vitro* studies,[9] and it has been speculated that local administration of the drug in the lung may maximize therapeutic benefits with fewer systemic side effects, as compared with systemic steroids.[3]

Therefore, it could be hypothesized that inhaled corticosteroids may be beneficial for patients with severe Covid-19. The hypothesis is further supported by reports that inhaled corticosteroids reduce the epithelial expression of genes linked to SARS-CoV-2 entry into host cells.[10 11] Among the inhaled corticosteroids, ciclesonide has been identified as a particularly promising treatment as it can suppress replication of SARS-CoV-2 *in vitro*.[12 13]

For peer review only - http://bmjopen.bmj.com/site/about/guidelines.xhtml

While previous randomized controlled trials have assessed the effects of inhaled budesonide[14 15] or ciclesonide[16 17] in non-hospitalized Covid-19 patients, no study has been performed in hospitalized patients with more severe Covid-19.

This open-label randomized controlled trial investigated the effects of inhaled ciclesonide, compared to standard care, in adult patients hospitalized with Covid-19 and requiring oxygen therapy.

For peer review only

Methods

Study design

The HALT Covid-19 (inHALation of ciclesonide for Treatment of Covid-19) trial was a multicenter, open-label randomized controlled trial to assess the efficacy and safety of inhaled ciclesonide for the treatment of hospitalized patients with Covid-19 receiving oxygen therapy. The trial was conducted at 9 hospitals (3 academic hospitals and 6 non-academic hospitals) in Sweden between June 1, 2020, and May 17, 2021.

All participants provided written informed consent. The study was approved by the Swedish Ethical Review Authority (Ethics committee number 2020-02183) and the Swedish Medical Products Agency (Eudra-CT number 2020-001928-34) and registered at clinicaltrials.gov (NCT04381364).

Protocol changes and rationale

For peer review only - http://bmjopen.bmj.com/site/about/guidelines.xhtml

The trial was designed in the beginning of the Covid-19 pandemic. After trial initiation, treatments for, and hospitalization rates of, patients with Covid-19 changed rapidly. Therefore, we made protocol changes (described in detail in the Online Appendix) and the trial was stopped early.

In brief, we increased the number of study centers, removed the upper age limit (≤85 years) for patient inclusion, changed the inclusion criteria from ≤48 hours since hospital admission to ≤48 hours from initiation of oxygen therapy and allowed for patients to be included on the basis of a positive antigen test for SARS-Cov-2. All changes were approved by the Data Monitoring Committee, Ethical Review Authority and the Swedish Medical Products Agency and implemented from December 2020.

In June 2021, 99 patients had been included in the study, a large and increasing proportion of the adult Swedish population had received Covid-19 vaccination and hospitalizations for Covid-19 had dropped substantially. We determined that it was unlikely that the intended sample size would be reached and asked the Data Monitoring Committee to convene for a meeting. Following the recommendation of the Data Monitoring Committee, the study was terminated for futility to meet the targeted enrolment.

Participants

Based on observations from Covid-19 patients treated at the study centers, we expected that 85% of the standard care group would survive and terminate oxygen

For peer review only - http://bmjopen.bmj.com/site/about/guidelines.xhtml

therapy within 30 days (median 8 days). We considered a 25% (2 days) reduction in the duration of oxygen therapy to be a clinically meaningful effect. We estimated that such an effect could be detected with α of 0.05, and 80% power if 446 participants (223 in each group) were enrolled.

Participants were eligible for inclusion if, they (1) were aged ≥18 years, (2) had a polymerase chain reaction confirmed SARS-CoV-2 infection or a positive SARS-CoV-2 antigen test from the upper respiratory tract, (3) were hospitalized at any of the study hospitals and (4) were receiving oxygen therapy, initiated within 48 hours before inclusion. Key exclusion criteria were ongoing treatment with inhaled or oral corticosteroids (previous use was accepted), oxygen therapy with >8 L oxygen/min or >50 % oxygen on nasal high-flow cannula, and ongoing or expected intensive care or palliative care (Online Appendix).

Randomization

Patients were randomized 1:1 in blocks of 8, stratified by sex and hospital to receive ciclesonide or standard care. The randomization sequence was prepared by a statistician not involved in the trial. Treatment allocation was provided through a web-based interface. The participants and the physicians treating them were unblinded to the treatment assignment.

Intervention

The treatment was 320 μg of inhaled ciclesonide (80 μg per actuation, for a total of 4 actuations, or 160 μg per actuation, for a total of 2 actuations) twice daily (total daily dose 640 μg) for 14 days. Ciclesonide was administered using a spacer (L'espace,

For peer review only - http://bmjopen.bmj.com/site/about/guidelines.xhtml

For peer review only

Nordic Infucare, Stockholm Sweden). Participants randomized to ciclesonide received written instructions, including pictures, and practical instructions on how to use the inhalator and spacer; the first dose was taken under supervision. Ciclesonide was then prescribed in the participant's electronic medical record and each given dose during the hospitalization was recorded. Participants discharged before day 14 were instructed to continue the treatment at home for a total treatment duration of 14 days. Participants randomized to standard care did not receive any intervention related to the study. Physicians treating the participants were not given any restrictions concerning treatments during the study period. Participants who had been discharged were contacted by telephone after day 30 for a follow-up interview.

Outcomes

The primary outcome was duration of oxygen therapy (time to termination of oxygen therapy in days) up to 30 days from randomization. Oxygen therapy was defined as terminated on the day after which the patient did not receive oxygen therapy during at least 48 hours, while being alive. This outcome corresponded to clinical improvement for patients receiving oxygen therapy according to the World Health Organization clinical progression scale.[18]

The key secondary outcome was a composite of invasive mechanical ventilation and death up to 30 days after randomization. Other secondary outcomes were each component of the key secondary outcome, admission to an intensive care unit, discharge from the hospital and dyspnea in daily living at 30-35 days after randomization as evaluated by the mMRC (Modified Medical Research Council)

For peer review only - http://bmjopen.bmj.com/site/about/guidelines.xhtml

dyspnea scale. The scale ranges from 0 to 4 with a higher score indicating more severe dyspnea.[19 20]

Data on serious adverse events[21] were collected by review of electronic medical records. Information about non-serious adverse events associated with ciclesonide use (dryness of mouth, nausea and oral candidiasis) was reported using a paper-based reporting form which was filled in by the treating physician. Information about non-serious adverse events occurring after hospital discharge was collected during the follow-up interview.

Data collection

Patient characteristics at baseline (comorbidities, comedications, clinical parameters) and study outcomes were obtained from electronic medical records. Investigators contacted participants after day 30 after randomization to ask them about non-serious adverse events and dyspnea in daily living (study outcome) at day 30-35 after randomization.

Statistical analysis

According to the pre-specified analysis plan in the study protocol, the analyses were performed by an investigator who had not been involved in the enrolment of participants and was blinded to treatment assignment. An intention-to-treat population was used. In the analysis of the duration of oxygen therapy, participants were followed from randomization to termination of oxygen therapy, death, or 30 days after randomization. Kaplan Meier cumulative incidence curves were generated to

For peer review only - http://bmjopen.bmj.com/site/about/guidelines.xhtml

illustrate the cumulative incidence of termination of oxygen therapy in the ciclesonide and standard care groups. A Cox proportional hazard regression model, adjusted for study hospital (Appendix Table 1), age (continuous variable) and sex was used to estimate hazard ratios (HR) with 95% CI for time-to-event outcomes. Proportions and the absolute risk difference with 95% CI were presented for binary outcomes. In a per-protocol analysis of the primary outcome, participants assigned to ciclesonide were censored at the time of discontinuing treatment. The median mMRC score was compared using the Kruskal-Wallis test. A logistic regression model adjusted for study hospital, age and sex was used to compare the likelihood of reporting a mMRC score of 0 (dyspnea only with strenuous exercise).

In an analysis that was not pre-specified, we additionally adjusted the primary outcome analysis for baseline variables, including days since symptom onset, c-reactive protein and white blood count (as continuous variables), and diabetes mellitus, hypertension and hyperlipidemia.

95% CIs of ratios not including 1 and 95% CIs for absolute risk differences not including 0 were considered statistically significant. Secondary outcome analyses were considered hypothesis-generating and no adjustment for multiple testing was made. Analyses were performed using Stata version 16.1 (StataCorp).

Patient and Public involvement

No patients were involved in setting the research question, nor in the design, conduct, or interpretation of the study.

For peer review only - http://bmjopen.bmj.com/site/about/guidelines.xhtml

Results

Of the 99 participants who underwent randomization 48 were assigned to receive ciclesonide and 51 to standard care (Figure 1). One participant in the standard care group withdrew consent and was excluded from the analysis. Ninety-eight patients (48 in the ciclesonide group and 50 in the standard care group) were included in the final analysis. All participants assigned to ciclesonide received the treatment at least once. None of the participants were lost to follow-up. The median age of participants was 59.5 (IQR 49, 67) years, 68% were men and the median duration of symptoms was 9 (IQR 8, 11) days. There were no relevant between-group differences in demographic characteristics, laboratory test results or comorbidities at enrollment (Table 1).

Table 1 Demographic and clinical characteristics of participants at study enrolment.

	**Total** (n=98)	**Ciclesonide** (n=48)	**Standard care** (n=50)
Age, median (IQR)	59.5 (49, 67)	61 (49, 67)	59 (49, 67)
Age ≥70 years, n (%)	78 (80)	37 (77)	41 (82)
Men, n (%)	67 (68)	34 (71)	33 (66)
Days since symptom onset, median (IQR)	9 (8, 11)	9 (7.5, 11.5)	10 (8, 11)
Days since symptom onset: <10 days, n (%)	51 (52)	27 (56)	24 (48)
Body mass index in kg/m^2, median (IQR)	29.7 (25.6, 34.0)	28.7 (25.4, 34.0)	30.6 (26.8, 34.3)
Oxygen flow of oxygen therapy in L/min, median (IQR)	2 (1, 3)	2 (1, 3)	2 (1, 2)
Respiratory rate per minute, median (IQR)	20 (18, 24)	20 (19, 25)	20 (18, 23)
C-reactive protein in mg/L, median (IQR)	100 (56, 142)	103 (62, 164)	91.5 (45.5, 124.5)

For peer review only - http://bmjopen.bmj.com/site/about/guidelines.xhtml

White cell count in x 10^9/L, median IQR	5.7 (4.5, 7.0)	5.3 (4.3, 6.9)	6.1 (4.9, 7.0)
eGFR in mL/min/1.73m^2, median (IQR)	83 (70.5, 90)	81.5 (70, 90)	87 (73, 90)
**Coexisting conditions, n (%)**			
Diabetes mellitus	18 (18)	8 (17)	10 (20)
Hypertension[a]	45 (46)	22 (46)	23 (46)
Hyperlipidemia[b]	27 (28)	12 (25)	15 (30)
Chronic obstructive lung disease	3 (3)	1 (2)	2 (4)
Asthma	8 (8)	6 (13)	2 (4)
Current smoker	12 (12)	6 (13)	6 (12)
Ischemic heart disease	8 (8)	2 (4)	6 (12)
Heart failure	3 (3)	2 (4)	1 (2)
Atrial fibrillation	5 (5)	3 (6)	2 (4)
Cancer	10 (10)	5 (10)	5 (10)
Chronic kidney disease	9 (9)	5 (10)	4 (8)

[a] Diagnosis of hypertension or use of antihypertensive drugs

[b] Diagnosis of hyperlipidemia or use of lipid lowering therapy

Missing values were: n=1 for days since symptom onset, n=20 for body mass index, n=1 for oxygen flow of oxygen therapy, n=1 for body temperature, n=1 for heart rate, n=3 for respiratory rate, n=3 for C-reactive protein, n=7 for white cell count and n=22 for eGFR.

eGFR: estimated glomerular filtration rate

The results of primary and secondary outcome analyses are presented in Table 2. Kaplan-Meier estimates of the median duration of oxygen therapy were 5.5 (IQR 3, 9) days in the ciclesonide group and 4 (2, 7) days in the standard care group. (Figure 2). The HR for termination of oxygen therapy during 30 days following randomization, used to compare ciclesonide vs standard care, showed that ciclesonide treatment was not statistically significantly associated with the duration of oxygen therapy (0.73 (95% CI 0.47 to 1.11)).

For peer review only - http://bmjopen.bmj.com/site/about/guidelines.xhtml

For peer review only

The research question that we aimed to assess was whether inhaled ciclesonide, as compared with standard care, could reduce the time to clinical improvement (as indicated by duration of oxygen therapy). While the interpretation of statistically non-significant findings is a recurring and well-known subject of debate in the medical literature, it is generally not recommended to use a binary interpretation based on an arbitrary cut-off for statistical significance[22-25]. This is particularly important in this trial as it was terminated early and thereby underpowered to assess its primary outcome. However, it has been suggested that in trials with statistically non-significant findings, the 95% CIs should be used to rule in or rule out potential effect sizes of the intervention. In this study, we therefore assessed the largest benefit of ciclesonide that was compatible with the confidence interval. Such a benefit was represented by the upper limit of the HR for time to termination of oxygen therapy (a higher HR indicates shorter duration of oxygen therapy for the ciclesonide group), i.e., 1.11. We took the inverse of this HR (1/1.11 = 0.90) to calculate the relative reduction in duration of oxygen therapy that the HR was compatible with (i.e., 1-0.90 = 10% relative reduction). We then multiplied this 10% relative reduction with the absolute duration of oxygen therapy in the standard care group to calculate the corresponding absolute difference in duration of oxygen therapy (10% * 4 days = 0.4 days, which is <1 day). Given the pre-specified minimally clinically important difference of 2 days (which was used for the power calculation of the study), we deemed this best-case difference to be clinically irrelevant.

In the per-protocol analysis, the HR for termination of oxygen therapy during 30 days following randomization was 0.79 (95% CI 0.51 to 1.23). In the additionally adjusted

For peer review only - http://bmjopen.bmj.com/site/about/guidelines.xhtml

analysis, the HR for termination of oxygen therapy was 0.68 (95% CI 0.43 to 1.09) (Table 2).

Table 2 Outcomes. All outcomes are recorded during 30 days following randomization unless otherwise indicated.

	Ciclesonide	Standard care	Difference[a]
**Primary outcome**			
Duration of oxygen therapy, median (IQR) days	5.5 (3, 9)	4 (2, 7)	0.73 (0.47 to 1.11)
**Key secondary outcome**			
Death or invasive mechanical ventilation, n (%)	3 (6)	3 (6)	0 (-9 to 10)
Time to death or invasive mechanical ventilation, median (IQR) days	2 (2, 10)	4 (2, 7)	0.90 (0.15 to 5.32)
**Secondary outcomes**			
Death, n (%)	2 (4)	1 (2)	-
Invasive mechanical ventilation, n (%)	1 (2)	3 (6)	-
Admission to an intensive care unit, n (%)	4 (8)	4 (8)	-
mMRC dyspnea scale score at day 30-35, median (IQR)[b]	3 (2, 4)	3 (2, 4)	0.97
mMRC dyspnea scale score 0 at day 30-35, n (%)[b]	4 (9)	7 (15)	0.48 (0.11 to 2.04)
**Per protocol analysis** [c.]			
Duration of oxygen therapy, median (IQR) days	5 (3, 9)	4 (2, 7)	0.79 (0.51 to 1.23)
**Additionally adjusted analysis**[d]			
Duration of oxygen therapy, median (IQR) days	5 (3, 9)	4.5 (2, 7)	0.68 (0.43 to 1.09)

[a.] Differences are expressed as hazard ratios (95% CI) estimated using a Cox proportional hazards model for time to event outcomes and as absolute risk difference (95% CI) in percent for outcomes of absolute risk. The comparison of the mMRC dyspnea score was done using the Kruskal-Wallis test and the difference is expressed as a p-value. The comparison of the likelihood of reporting a mMRC score of 0 was done using a logistic regression model and the difference is expressed as an odds ratio (95% CI). Statistical testing for differences in proportions and time-to-event analyses were not performed for the secondary outcome events, including death, invasive mechanical ventilation, and admission to an intensive care unit due to few events.

For peer review only - http://bmjopen.bmj.com/site/about/guidelines.xhtml

[b.] Not including 1 participant in the standard care group and 2 participants in the ciclesonide group who died within 30 days of randomization and 1 participant in the standard care group and 1 participant in the ciclesonide group with missing data on this outcome.

[c.] In the per-protocol analysis for duration of oxygen therapy, patients assigned to ciclesonide were censored at the time of discontinuing treatment.

[d.] In addition to age, sex and study center, this analysis of duration of oxygen therapy was adjusted for days since symptom onset, c-reactive protein, white blood count (as continuous variables) and diabetes mellitus, hypertension and hyperlipidemia (as categorical variables). The analyses included n=46 in the standard care group and n=45 in the ciclesonide group without missing data on any of the variables included in the model.

In total, 3 (6%) participants assigned to ciclesonide and 3 (6%) participants assigned to standard care experienced the key secondary outcome of mechanical invasive ventilation or death (absolute difference 0% (95% CI -10 to 9%; HR 0.90 (95% CI 0.15 to 5.32)). Median mMRC dyspnea score at 30-35 days after randomization was 3 (IQR 2, 4) in both groups (p-value for difference 0.97) (Table 2).

There were no apparent differences between the groups in treatments that participants received after randomization (Table 3); 26 (54%) of the participants assigned to ciclesonide and 22 (44%) of the participants in the standard care group received treatment with systemic corticosteroids after randomization.

Few serious adverse clinical events occurred during the study. The most frequently reported adverse event was dry mouth (7 (15%) participants in the ciclesonide group and 11 (22%) participants in the standard care group). Two participants assigned to ciclesonide and 0 in the placebo group reported that they experienced oral

For peer review only - http://bmjopen.bmj.com/site/about/guidelines.xhtml

candidiasis (Table 3).

Table 3 Participants' treatments and adverse clinical events through day 30 after randomization.

	**Ciclesonide** (n=48)	**Standard care** (n=50)
**Received treatment, n (%)**		
Systemic corticosteroids	26 (54)	22 (44)
Remdesivir	4 (8)	5 (10)
Low-molecular-weight heparin	45 (94)	45 (90)
Oral anticoagulants	32 (67)	30 (60)
Vasopressors	4 (8)	3 (6)
Non-invasive mechanical ventilation	8 (17)	7 (14)
**Serious clinical events, n (%)**		
Renal failure	2 (4)	3 (6)
Cardiac arrest	1 (2)	0 (0)
New onset atrial fibrillation	0 (0)	1 (2)
Pulmonary embolism	4 (8)	2 (4)
Other thromboembolic events	0 (0)	1 (2)
Sepsis	3 (6)	2 (4)
Other serious event	1 (2)	0 (0)
**Non-serious adverse events, n (%)**		
Nausea	6 (13)	8 (16)
Dry mouth	7 (15)	11 (22)
Oral candidiasis	2 (4)	0 (0)
Other non-serious adverse event	3 (6)	1 (2)

For peer review only - http://bmjopen.bmj.com/site/about/guidelines.xhtml

Some pre-specified analyses were not performed due to small sample size or low number of events. These included statistical testing of differences in proportions and time-to-event analyses for non-key secondary outcomes, including death, invasive mechanical ventilation, and admission to an intensive care unit; the secondary outcome analyses of discharge from hospital; subgroup analyses, and the primary outcome analysis after exclusion of participants who received invasive mechanical ventilation or died.

Discussion

In this randomized open-label, controlled trial, including 98 hospitalized Covid-19 patients with ongoing oxygen therapy, treatment with inhaled ciclesonide did not result in a statistically significant reduction in the duration of oxygen therapy, used as a measure of time to clinical improvement. The trial ruled out, with 0.95 confidence, treatments effects of ciclesonide corresponding to more than a one-day reduction in duration of oxygen therapy.

While previous randomized controlled trials have assessed effects of inhaled corticosteroids, including budesonide[14 15] and ciclesonide[16 17], in non-hospitalized patients with Covid-19, this is the first trial that includes hospitalized patients with more severe forms of the disease. In contrast to our hypothesis, the median duration of oxygen therapy was nominally longer among patients assigned to ciclesonide vs standard care (5.5 vs 4 days; HR for termination of oxygen therapy 0.73 (95% CI 0.47 to 1.11)). As such, the 95% CI indicates that,[24] even in the best case, ciclesonide may reduce the duration of oxygen therapy with only 10% (1-1/1.11; less

For peer review only - http://bmjopen.bmj.com/site/about/guidelines.xhtml

than 1 day in our study) while it may in the worst case result in an over 2-fold increase. Thus, the results of this trial indicate that ciclesonide is unlikely to provide a clinically meaningful beneficial effect on the duration of oxygen therapy in hospitalized Covid-19 patients receiving oxygen therapy.

To date, 2 randomized controlled trials of ciclesonide in non-hospitalized patients with Covid-19 have been presented. In the CONTAIN study,[16] which was terminated early due to slow recruitment, 215 non-hospitalized patients with a median of 3 days symptom duration were randomized to combination treatment with intranasal and inhaled ciclesonide or placebo. No statistically significant difference between the groups was observed for the primary endpoint, resolution of respiratory symptoms at day 7 after randomization, which was reached by 40% of the patients in the treatment group vs 35% in the placebo group (adjusted risk difference of 5.5% (95% CI -7.8% to 18.8%).[16] Six (6%) patients assigned to ciclesonide vs 3 (3%) in the placebo group were hospitalized within 14 days; none died. In another clinical trial of ciclesonide, including 400 non-hospitalized patients with Covid-19,[17] randomization to ciclesonid vs placebo did not result in a reduced time to alleviation of all Covid-19 related symptoms. However, in secondary outcome analyses, patients assigned to ciclesonide had fewer emergency department visits or hospital admissions for reasons related to COVID-19 (odds ratio, 0.18, 95% CI, 0.04 to 0.85).

In addition, 2 randomized clinical trials of the inhaled corticosteroid budesonide in non-hospitalized patients with Covid-19 have been presented. The STOIC trial was an open-label trial comparing inhaled budesonide vs standard care in 146 Covid-19 patients with mild symptoms.[14] Compared to standard care, budesonide treatment

For peer review only - http://bmjopen.bmj.com/site/about/guidelines.xhtml

led to a statistically significant reduction in Covid-19-related emergency department assessment and hospitalization (difference in proportions 0.123 (95% CI 0.043 to 0.218).[14] Furthermore, budesonide treatment was associated with 1 day shorter time to clinical recovery. The PRINCIPLE trial was another open-label trial that included 4700 primary care patients at high risk of developing severe Covid-19 (1073 randomized to budesonide treatment; 1988 to standard care; 1639 to other treatments).[15] Compared to standard care, randomization to budesonide led to a shorter time to self-reported recovery (difference 2.94 days (95% Bayesian credible interval 1.19 to 5.12) and a reduced likelihood of hospital admission or death, although the results for the latter outcomes did not meet the superiority threshold.

Taken together, the previous studies indicate that inhaled corticosteroids might be useful for preventing deterioration of Covid-19 in non-hospitalized patients with mild symptoms. It is possible that the low likelihood of benefit associated with ciclesonide treatment observed in our study reflects the more severe pulmonary inflammation in our study population, as indicated by the need for hospitalization with oxygen therapy and a median symptom duration of 9 days: at such stages of disease progression, it could be speculated that pulmonary administration of corticosteroids may not suffice to confer benefit and that systemic treatment is needed. Accordingly, in the Recovery trial of hospitalized Covid-19 patients,[26] dexamethasone treatment reduced risk of death and the time to discharge from hospital, with these benefits primarily being observed among patients receiving oxygen therapy or invasive mechanical ventilation at baseline.

Similar to other clinical trials including patients with Covid-19,[15 26 27] we used a

For peer review only - http://bmjopen.bmj.com/site/about/guidelines.xhtml

pragmatic, open-label design. With this design, we intended to assess the effect of adding ciclesonide to standard care, rather than to examine the effect of ciclesonide compared to placebo. The research question that our study aimed to answer was “what is the effect of using ciclesonide as an addition to standard care as compared with standard care alone?” While this is a research question of relevance to clinical decision-making, the open-label design and the possible expectations of effect among both patients[28] and physicians might have affected the outcomes in our study, including when to terminate oxygen therapy. Another limitation of our study is that we were unable to recruit the intended number of patients due to the substantial decrease in hospitalized Covid-19 patients in Sweden during 2021. Importantly, the study could not provide much information regarding the key secondary outcome of death or invasive mechanical intervention. Further research in hospitalized Covid-19 patients is needed to determine the potential effect of ciclesonide treatment on these outcomes. Moreover, it is a possibility that effects of ciclesonide differ as compared to other inhaled corticosteroids (e.g., budesonide). Patients were instructed to use ciclesonide without a spacer after discharge from the hospital; this may have affected drug delivery. Finally, results from the Recovery Trial were released 5 weeks after the initiation of our study and around half of the patients in both the ciclesonide group and the control group received systemic corticosteroids after randomization. Further studies would be needed to assess the comparative effectiveness and safety of ciclesonide vs systemic corticosteroids.

Conclusions

In this open-label randomized controlled trial in patients hospitalized with Covid-19 and receiving oxygen therapy, the findings indicated that treatment with ciclesonide

For peer review only - http://bmjopen.bmj.com/site/about/guidelines.xhtml

vs standard care is unlikely to result in a clinically meaningful reduction in the duration of oxygen therapy.

Acknowledgements

We would like to thank the following individuals that did not qualify for authorship but contributed to the study: Dr Oscar Bakhouch (Skaraborg Hospital), Dr Eva-Marie Boman and Dr Anders Lundqvist (Southern Älvsborg Hospital).

Contributorship statement

DB, PU, PT, OB and DPA conceived the study and were responsible for the methods. OB and DPA were responsible for the study conduct. DB, PU, OB and DPA were responsible for the financing. PT validated the data. PU performed the main analysis. DPA, PU and PT wrote the original draft of the manuscript. All authors wrote, reviewed, and edited the manuscript. OB and DPA supervised the study. DB, DPA, PU, PT and OB were responsible for administration of the project. DPA and OB are the guarantors. DB, PT, AK, EW, SA, SW, OE, AN, AE, JG, JEK, SB, CU, ML, BJ, JL, JLI, JH, OB and DPA enrolled participants in the study. The corresponding author attests that all listed authors meet authorship criteria and that no others meeting the criteria have been omitted.

Competing interests

This study received non-financial support from COVIS Pharma (study drug donation). The authors have no conflict of interest to disclose.

For peer review only

For peer review only - http://bmjopen.bmj.com/site/about/guidelines.xhtml

Funding/Support

This study was funded by the Swedish Heart and Lung Foundation (Number 20200421), The Axel and Margaret Ax:son Johnson Foundation (N/A), CIMED (N/A), and Strategic Research Program at Karolinska Institutet (Number 961507), the Stockholm County Council (Number: 954970, 963296, 962029), and the Västmanland County Council (Grant nr LTV-938409). PU was supported by grants from the Strategic Research Program in Epidemiology at Karolinska Institutet (N/A), and a Faculty Funded Career Position at Karolinska Institutet (N/A).

The funders had no role in the study design, conduct, collection, management, analysis, interpretation of data, writing or reviewing the manuscript or decision to submit the manuscript for publication. The study drug was donated by COVIS Pharma but COVIS pharma did not participate in any other part of the study.

For peer review only

Data sharing statement

Data are available upon resonable request

Ethics Approval

All participants provided written informed consent. The study was approved by the Swedish Ethical Review Authority (Ethics committee number 2020-02183) and the Swedish Medical Products Agency (Eudra-CT number 2020-001928-34) and registered at clinicaltrials.gov (NCT04381364).

References

For peer review only - http://bmjopen.bmj.com/site/about/guidelines.xhtml

1. Gibson PG, Qin L, Puah SH. COVID-19 acute respiratory distress syndrome (ARDS): clinical features and differences from typical pre-COVID-19 ARDS. *Med J Aust* 2020;213(2):54-56 e1. doi: 10.5694/mja2.50674 [published Online First: 2020/06/24]
2. Bos LDJ. COVID-19-related Acute Respiratory Distress Syndrome: Not So Atypical. *Am J Respir Crit Care Med* 2020;202(4):622-24. doi: 10.1164/rccm.202004-1423LE [published Online First: 2020/06/25]
3. Festic E, Carr GE, Cartin-Ceba R, et al. Randomized Clinical Trial of a Combination of an Inhaled Corticosteroid and Beta Agonist in Patients at Risk of Developing the Acute Respiratory Distress Syndrome. *Crit Care Med* 2017;45(5):798-805. doi: 10.1097/CCM.0000000000002284 [published Online First: 2017/02/28]
4. Mohamed HS, Meguid MM. Effect of nebulized budesonide on respiratory mechanics and oxygenation in acute lung injury/acute respiratory distress syndrome: Randomized controlled study. *Saudi J Anaesth* 2017;11(1):9-14. doi: 10.4103/1658-354X.197369 [published Online First: 2017/02/22]
5. Jansson AH, Eriksson C, Wang X. Effects of budesonide and N-acetylcysteine on acute lung hyperinflation, inflammation and injury in rats. *Vascul Pharmacol* 2005;43(2):101-11.

For peer review only - http://bmjopen.bmj.com/site/about/guidelines.xhtml

doi: 10.1016/j.vph.2005.03.006 [published Online First: 2005/06/22]

6. Forsgren PE, Modig JA, Dahlback CM, et al. Prophylactic treatment with an aerosolized corticosteroid liposome in a porcine model of early ARDS induced by endotoxaemia. *Acta Chir Scand* 1990;156(6-7):423-31. [published Online First: 1990/06/01]
7. Wang J, Zhang L, Walther SM. Administration of aerosolized terbutaline and budesonide reduces chlorine gas-induced acute lung injury. *J Trauma* 2004;56(4):850-62. doi: 10.1097/01.ta.0000078689.45384.8b [published Online First: 2004/06/10]
8. Walther S, Jansson I, Berg S, et al. Pulmonary granulocyte accumulation is reduced by nebulized corticosteroid in septic pigs. *Acta Anaesthesiol Scand* 1992;36(7):651-5. doi: 10.1111/j.1399-6576.1992.tb03537.x [published Online First: 1992/10/01]
9. Yamaya M, Nishimura H, Deng X, et al. Inhibitory effects of glycopyrronium, formoterol, and budesonide on coronavirus HCoV-229E replication and cytokine production by primary cultures of human nasal and tracheal epithelial cells. *Respir Investig* 2020;58(3):155-68. doi: 10.1016/j.resinv.2019.12.005 [published Online First: 2020/02/26]

For peer review only

For peer review only - http://bmjopen.bmj.com/site/about/guidelines.xhtml

10. Peters MC, Sajuthi S, Deford P, et al. COVID-19-related Genes in Sputum Cells in Asthma. Relationship to Demographic Features and Corticosteroids. *Am J Respir Crit Care Med* 2020;202(1):83-90. doi: 10.1164/rccm.202003-0821OC [published Online First: 2020/04/30]
11. Milne S, Li X, Yang CX, et al. Inhaled corticosteroids downregulate SARS-CoV-2-related genes in COPD: results from a randomised controlled trial. *Eur Respir J* 2021;58(1) doi: 10.1183/13993003.00130-2021 [published Online First: 2021/04/03]
12. Jeon S, Ko M, Lee J, et al. Identification of Antiviral Drug Candidates against SARS-CoV-2 from FDA-Approved Drugs. *Antimicrob Agents Chemother* 2020;64(7) doi: 10.1128/AAC.00819-20 [published Online First: 2020/05/06]
13. Kimura H, Kurusu H, Sada M, et al. Molecular pharmacology of ciclesonide against SARS-CoV-2. *J Allergy Clin Immunol* 2020;146(2):330-31. doi: 10.1016/j.jaci.2020.05.029 [published Online First: 2020/07/01]
14. Ramakrishnan S, Nicolau DV, Jr., Langford B, et al. Inhaled budesonide in the treatment of early COVID-19 (STOIC): a phase 2, open-label, randomised controlled trial. *Lancet*

For peer review only - http://bmjopen.bmj.com/site/about/guidelines.xhtml

Respir Med 2021;9(7):763-72. doi: 10.1016/S2213-2600(21)00160-0 [published Online First: 2021/04/13]

15. Yu LM, Bafadhel M, Dorward J, et al. Inhaled budesonide for COVID-19 in people at high risk of complications in the community in the UK (PRINCIPLE): a randomised, controlled, open-label, adaptive platform trial. *Lancet* 2021;398(10303):843-55. doi: 10.1016/S0140-6736(21)01744-X [published Online First: 2021/08/14]
16. Ezer N, Belga S, Daneman N, et al. Inhaled and intranasal ciclesonide for the treatment of covid-19 in adult outpatients: CONTAIN phase II randomised controlled trial. *BMJ* 2021;375:e068060. doi: 10.1136/bmj-2021-068060 [published Online First: 2021/11/04]
17. Clemency BM, Varughese R, Gonzalez-Rojas Y, et al. Efficacy of Inhaled Ciclesonide for Outpatient Treatment of Adolescents and Adults With Symptomatic COVID-19: A Randomized Clinical Trial. *JAMA Intern Med* 2021 doi: 10.1001/jamainternmed.2021.6759 [published Online First: 2021/11/23]
18. Characterisation WHOWGotC, Management of C-i. A minimal common outcome measure set for COVID-19 clinical research. *Lancet Infect Dis* 2020;20(8):e192-e97. doi: 10.1016/S1473-3099(20)30483-7 [published Online First: 2020/06/17]

For peer review only

19. Mahler DA, Wells CK. Evaluation of clinical methods for rating dyspnea. *Chest* 1988;93(3):580-6. doi: 10.1378/chest.93.3.580 [published Online First: 1988/03/01]

20. Bestall JC, Paul EA, Garrod R, et al. Usefulness of the Medical Research Council (MRC) dyspnoea scale as a measure of disability in patients with chronic obstructive pulmonary disease. *Thorax* 1999;54(7):581-6. doi: 10.1136/thx.54.7.581 [published Online First: 1999/06/22]

21. Directive 2001/20/EC of the European Parliament and of the Council of 4 April 2001 on the approximation of the laws, regulations and administrative provisions of the member states relating to the implementation of good clinical practice in the conduct of clinical trials on medicinal products for human use. *Med Etika Bioet* 2002;9(1-2):12-9. [published Online First: 2005/11/10]

22. Altman DG, Bland JM. Absence of evidence is not evidence of absence. *BMJ* 1995;311(7003):485. doi: 10.1136/bmj.311.7003.485 [published Online First: 1995/08/19]

23. Harrell F. Statistical Errors in the Medical Literature. Statistical Thinking. https://www.fharrell.com/post/errmed/index.html (accessed Oct 24, 2022). ed, 2017.

For peer review only - http://bmjopen.bmj.com/site/about/guidelines.xhtml

24. Hawkins AT, Samuels LR. Use of Confidence Intervals in Interpreting Nonstatistically Significant Results. *JAMA* 2021;326(20):2068-69. doi: 10.1001/jama.2021.16172 [published Online First: 2021/11/24]

25. Greenland S, Senn SJ, Rothman KJ, et al. Statistical tests, P values, confidence intervals, and power: a guide to misinterpretations. *Eur J Epidemiol* 2016;31(4):337-50. doi: 10.1007/s10654-016-0149-3 [published Online First: 2016/05/23]

26. Group RC, Horby P, Lim WS, et al. Dexamethasone in Hospitalized Patients with Covid-19. *N Engl J Med* 2021;384(8):693-704. doi: 10.1056/NEJMoa2021436 [published Online First: 2020/07/18]

27. Investigators R-C, Gordon AC, Mouncey PR, et al. Interleukin-6 Receptor Antagonists in Critically Ill Patients with Covid-19. *N Engl J Med* 2021;384(16):1491-502. doi: 10.1056/NEJMoa2100433 [published Online First: 2021/02/26]

28. Wechsler ME, Kelley JM, Boyd IO, et al. Active albuterol or placebo, sham acupuncture, or no intervention in asthma. *N Engl J Med* 2011;365(2):119-26. doi: 10.1056/NEJMoa1103319 [published Online First: 2011/07/15]

For peer review only

For peer review only

For peer review only - http://bmjopen.bmj.com/site/about/guidelines.xhtml

Figure 1 Flow diagram for study participants.

Figure 2 Time to termination of oxygen therapy during 30 days after randomization.

For peer review only

For peer review only - http://bmjopen.bmj.com/site/about/guidelines.xhtml

Figure 1

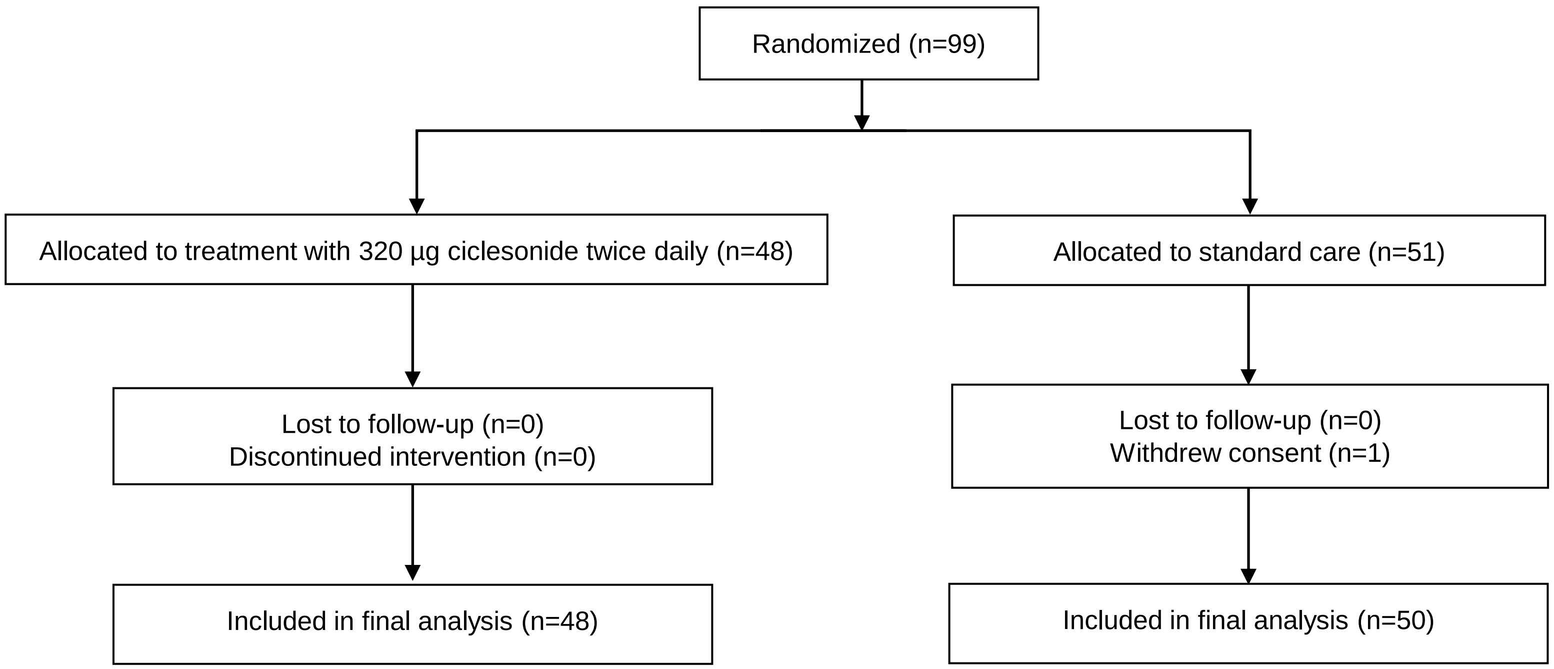
Randomized (n=99)
Allocated to treatment with 320 µg ciclesonide twice daily (n=48)
Allocated to standard care (n=51)
Lost to follow-up (n=0)
Discontinued intervention (n=0)
Lost to follow-up (n=0)
Withdrew consent (n=1)
Included in final analysis (n=48)
Included in final analysis (n=50)

Figure 2 Time to termination of oxygen therapy.

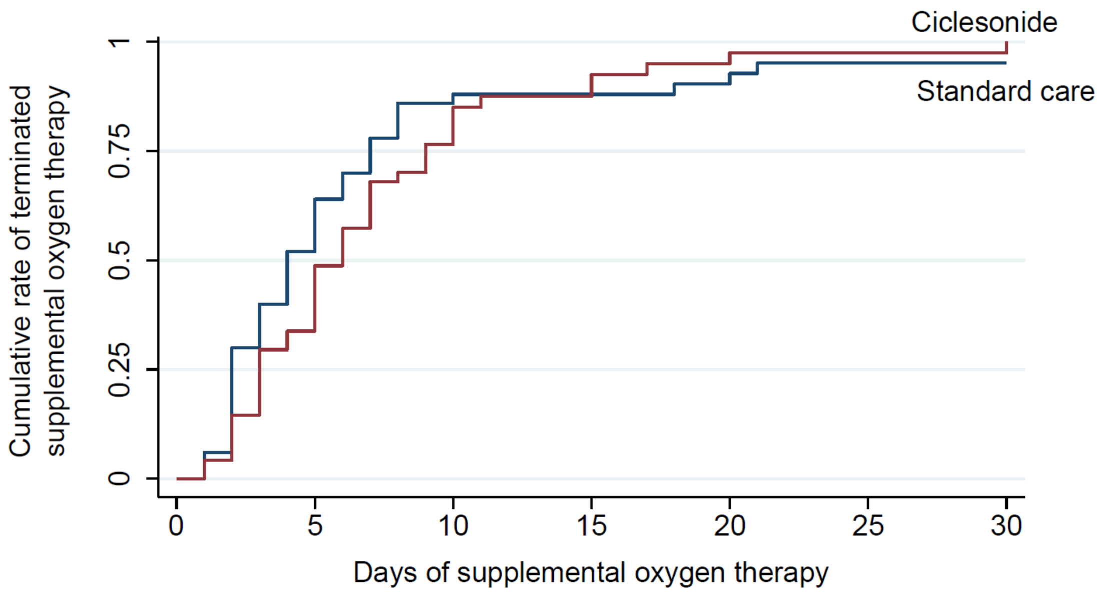

No at risk

Standard care	50	24	7	6	4	2	2
Ciclesonide	48	31	11	5	2	1	1

ew only

ONLINE APPENDIX

Brodin D, Tornhammar P, Ueda P, Krifors A, Westerlund E, Athlin S, Wojt S, Elvstam O, Neumann A, Elshani A, Giesecke J, Edvardsson J, Bunpuckdee S, Unge C, Larsson M, Johansson B, Ljungberg J, Lindell J, Hansson J, Blennow O, Andersson DP. Inhaled Ciclesonide in Adults Hospitalized with Covid-19: a Randomized Controlled Open-label Trial (HALT Covid-19).

For peer review only

Protocol changes and rationale

The trial was designed in the beginning of the covid-19 pandemic when data from randomized clinical trials of Covid-19 treatment were scarce. After trial initiation, treatments for patients with Covid-19 and hospitalization rates of such patients changed rapidly. Therefore, we made changes to the protocol and the trial was stopped early.

5 weeks after the start of patient inclusion in our study, in July 2020, the Recovery Collaborative group presented preliminary data[1] showing protective effects of dexamethasone treatment in patients hospitalized for covid-19; a subgroup analysis of this study indicated that the effect was driven by patients receiving invasive mechanical ventilation or oxygen therapy. These data, in combination with local experience from treating patients with Covid-19,[2] led to most patients receiving oxygen therapy with ≥ 4 L oxygen/min at the study hospitals being treated with systemic corticosteroids. As use of systemic corticosteroids was an exclusion criterion, the change in practice made a large proportion of the Covid-19 patients ineligible for participation.

Initially the trial was conducted at 4 hospitals. To increase the inclusion rate, 9 additional hospitals were included as study sites, although only 5 of them ended up recruiting patients to the study. We also removed the previous upper age limit of 85 years for inclusion and allowed for inclusion of patients based on a positive antigen test for SARS-CoV-2. Moreover, because some patients may start receiving oxygen therapy before hospital admission (e.g., at nursing homes before being transported

For peer review only

to the hospital) or a period after hospital admission (e.g., if the patient's condition deteriorated) and we aimed to include patients shortly after initiation of such therapy, we changed the inclusion criteria from hospitalization within 48 hours prior to enrollment to initiation of oxygen therapy no longer than 48 hours prior to enrollment.

All changes were approved by the Data Monitoring Committee, Ethical Review Authority and the Swedish Medical Products Agency and implemented from December 2020.

In June 2021, when 99 patients had been included in the study, a large and increasing proportion of the adult Swedish population had received vaccination for Covid-19. The number of patients hospitalized with Covid-19 had dropped substantially and there were none to only a few Covid-19 patients admitted to the study hospitals per week. We determined that it was unlikely that we would reach the intended sample size and asked the Data Monitoring Committee to convene for a meeting. Following the recommendation of the Data Monitoring Committee, the study was terminated early due to expected futility to meet total enrolment.

Inclusion and exclusion criteria[a]

Participants were eligible for inclusion if, at the time of study inclusion, they (1) were aged ≥18 years, (2) had a polymerase chain reaction confirmed SARS-CoV-2 infection or a positive antigen test for SARS-CoV-2, (3) were hospitalized at any of the study hospitals and (4) were receiving oxygen therapy with not more than 48 hours having passed since initiation of this treatment.

Patients were not eligible for inclusion if they (1) had a history of hypersensitivity to ciclesonide or other substances included in the treatment, (2) received ongoing treatment with inhaled or oral corticosteroids, ketokonazol, itrakonazol, ritonavir or nelfinavir, (3) received >8 L oxygen/min or >50 % oxygen with nasal high-flow therapy, (4) were receiving or under consideration for palliative care or had an expected survival of less than 72 h, (5) were expected to be admitted to an intensive care unit within 48 h, (6) had active or inactive pulmonary tuberculosis, severe liver failure (Child-Pugh C), pulmonary arterial hypertension or fibrosis, cognitive or physical impairment, (7) had insufficient language skills to understand information given about the study, (8) had been included in a clinical trial within 30 days, or (9) were women and pregnant, breastfeeding or did not agree to take highly effective contraceptive measures while receiving treatment plus an additional 7 days.

[a] The presentation of these inclusion and exclusion criteria have been modified for readability as compared with the version presented in the study protocol.

For peer review only

Appendix table 1 Number of participants included in the final study population by study center.

Study center	*n participants*
Danderyd Hospital	26
Capio S:t Göran Hospital	24
Karolinska University Hospital	21
Västmanland County Hospital	13
Örebro University Hospital[a]	6
Växsjö Central Hospital[a]	3
Halland County Hospital[a]	2
Östersund Hospital[a]	2
Visby Hospital[a]	1

[a] In the analyses adjusted for study center, these hospitals were categorized into one group.

For peer review only

References

1. Group RC, Horby P, Lim WS, et al. Dexamethasone in Hospitalized Patients with Covid-19. *N Engl J Med* 2021;384(8):693-704. doi: 10.1056/NEJMoa2021436 [published Online First: 2020/07/18]

2. Kan B, Ahl M, Blennow O, et al. *Lakartidningen* 2020;117 [published Online First: 2020/10/07]

For peer review only

CONSORT 2010 checklist of information to include when reporting a randomised trial*

Section/Topic	Item No	Checklist item	Reported on page No
**Title and abstract**			
	1a	Identification as a randomised trial in the title	1
	1b	Structured summary of trial design, methods, results, and conclusions (for specific guidance see CONSORT for abstracts)	3
**Introduction**			
Background and objectives	2a	Scientific background and explanation of rationale	5,6
	2b	Specific objectives or hypotheses	5-7
**Methods**			
Trial design	3a	Description of trial design (such as parallel, factorial) including allocation ratio	6-9
	3b	Important changes to methods after trial commencement (such as eligibility criteria), with reasons	6,7,9
Participants	4a	Eligibility criteria for participants	7,8
	4b	Settings and locations where the data were collected	6,7
Interventions	5	The interventions for each group with sufficient details to allow replication, including how and when they were actually administered	6-11
Outcomes	6a	Completely defined pre-specified primary and secondary outcome measures, including how and when they were assessed	9-11
	6b	Any changes to trial outcomes after the trial commenced, with reasons	6,7
Sample size	7a	How sample size was determined	6-9
	7b	When applicable, explanation of any interim analyses and stopping guidelines	6,7
Randomisation:			
Sequence generation	8a	Method used to generate the random allocation sequence	8
	8b	Type of randomisation; details of any restriction (such as blocking and block size)	8
Allocation concealment mechanism	9	Mechanism used to implement the random allocation sequence (such as sequentially numbered containers), describing any steps taken to conceal the sequence until interventions were assigned	8
Implementation	10	Who generated the random allocation sequence, who enrolled participants, and who assigned participants to interventions	8
Blinding	11a	If done, who was blinded after assignment to interventions (for example, participants, care providers, those	8,11

For peer review only

		assessing outcomes) and how	
	11b	If relevant, description of the similarity of interventions	-
Statistical methods	12a	Statistical methods used to compare groups for primary and secondary outcomes	10,11
	12b	Methods for additional analyses, such as subgroup analyses and adjusted analyses	10,11
**Results**			
Participant flow (a diagram is strongly recommended)	13a	For each group, the numbers of participants who were randomly assigned, received intended treatment, and were analysed for the primary outcome	11-13
	13b	For each group, losses and exclusions after randomisation, together with reasons	11-13
Recruitment	14a	Dates defining the periods of recruitment and follow-up	6,7
	14b	Why the trial ended or was stopped	7
Baseline data	15	A table showing baseline demographic and clinical characteristics for each group	Yes
Numbers analysed	16	For each group, number of participants (denominator) included in each analysis and whether the analysis was by original assigned groups	Yes
Outcomes and estimation	17a	For each primary and secondary outcome, results for each group, and the estimated effect size and its precision (such as 95% confidence interval)	Yes
	17b	For binary outcomes, presentation of both absolute and relative effect sizes is recommended	-
Ancillary analyses	18	Results of any other analyses performed, including subgroup analyses and adjusted analyses, distinguishing pre-specified from exploratory	10,11
Harms	19	All important harms or unintended effects in each group (for specific guidance see CONSORT for harms)	13
**Discussion**			
Limitations	20	Trial limitations, addressing sources of potential bias, imprecision, and, if relevant, multiplicity of analyses	13-17
Generalisability	21	Generalisability (external validity, applicability) of the trial findings	13-17
Interpretation	22	Interpretation consistent with results, balancing benefits and harms, and considering other relevant evidence	13-17
**Other information**			
Registration	23	Registration number and name of trial registry	6
Protocol	24	Where the full trial protocol can be accessed, if available	-
Funding	25	Sources of funding and other support (such as supply of drugs), role of funders	18,19

*We strongly recommend reading this statement in conjunction with the CONSORT 2010 Explanation and Elaboration for important clarifications on all the items. If relevant, we also recommend reading CONSORT extensions for cluster randomised trials, non-inferiority and equivalence trials, non-pharmacological treatments, herbal interventions, and pragmatic trials. Additional extensions are forthcoming: for those and for up to date references relevant to this checklist, see www.consort-statement.org.

For peer review only - http://bmjopen.bmj.com/site/about/guidelines.xhtml

BMJ Open

Inhaled Ciclesonide in Adults Hospitalized with Covid-19: a Randomized Controlled Open-label Trial (HALT Covid-19)

Journal:	*BMJ Open*
Manuscript ID	bmjopen-2022-064374.R3
Article Type:	Original research
Date Submitted by the Author:	02-Feb-2023
Complete List of Authors:	Brodin, Daniel; Capio Sankt Görans Sjukhus<br>Tornhammar, Per; Karolinska Institute, Functional Area of Emergency Medicine<br>Ueda, Peter; Karolinska Institutet<br>Krifors, Anders; Karolinska Institutet, Department of Physiology and Pharmacology; Västmanlands sjukhus Västerås<br>Westerlund, Eli; Danderyd University Hospital, Department of Clinical Sciences<br>Athlin, Simon; Örebro University<br>Wojt, Sandra; Danderyd University Hospital, Department of Internal Medicine<br>Elvstam, Olof; Central Hospital Växjö, Department of Infectious Diseases<br>Neumann, Anca; Capio Sankt Görans Sjukhus<br>Elshani, Arsim; Karlskoga Hospital<br>Giesecke, Julia; Karolinska Universitetssjukhuset<br>Edvardsson-Källkvist, Jens; Karolinska University Hospital<br>Bunpuckdee, Sayam; Karolinska Institutet<br>Unge, Christian; Danderyd University Hospital, Department of Internal Medicine<br>Larsson, Martin; Karolinska Institutet Department of Clinical Science and Education Sodersjukhuset<br>Johansson, Björn; Halland County<br>Ljungberg, Johan; Halland County<br>Lindell, Jonas; Visby Hospital, Department of Infectious Diseases<br>Hansson, Johan; Östersund Hospital, Department of Infectious Diseases<br>Blennow, Ola; Karolinska University Hospital, Department of Infectious Diseases<br>Andersson, Daniel Peter; Karolinska University Hospital, Department of Medicine Huddinge H7, Karolinska Institutet; Karolinska Institute, Medicine (H7)
**Primary Subject Heading**:	Infectious diseases
Secondary Subject Heading:	Respiratory medicine
Keywords:	COVID-19, Clinical trials < THERAPEUTICS, Respiratory infections < THORACIC MEDICINE

I, the Submitting Author has the right to grant and does grant on behalf of all authors of the Work (as defined in the below author licence), an exclusive licence and/or a non-exclusive licence for contributions from authors who are: i) UK Crown employees; ii) where BMJ has agreed a CC-BY licence shall apply, and/or iii) in accordance with the terms applicable for US Federal Government officers or employees acting as part of their official duties; on a worldwide, perpetual, irrevocable, royalty-free basis to BMJ Publishing Group Ltd ("BMJ") its licensees and where the relevant Journal is co-owned by BMJ to the co-owners of the Journal, to publish the Work in this journal and any other BMJ products and to exploit all rights, as set out in our licence.

The Submitting Author accepts and understands that any supply made under these terms is made by BMJ to the Submitting Author unless you are acting as an employee on behalf of your employer or a postgraduate student of an affiliated institution which is paying any applicable article publishing charge ("APC") for Open Access articles. Where the Submitting Author wishes to make the Work available on an Open Access basis (and intends to pay the relevant APC), the terms of reuse of such Open Access shall be governed by a Creative Commons licence – details of these licences and which Creative Commons licence will apply to this Work are set out in our licence referred to above.

Other than as permitted in any relevant BMJ Author's Self Archiving Policies, I confirm this Work has not been accepted for publication elsewhere, is not being considered for publication elsewhere and does not duplicate material already published. I confirm all authors consent to publication of this Work and authorise the granting of this licence.

For peer review only

Inhaled Ciclesonide in Adults Hospitalized with Covid-19: a Randomized Controlled Open-label Trial (HALT Covid-19)

Daniel Brodin[1*], MD; Per Tornhammar[2,3*], MD; Peter Ueda[4*¤], MD, PhD; Anders Krifors[5,6] MD; Eli Westerlund[7], MD, PhD; Simon Athlin[8], MD, PhD; Sandra Wojt[9], MD; Olof Elvstam[10], MD, PhD; Anca Neumann[1], MD; Arsim Elshani[11], MD; Julia Giesecke[3] MD; Jens Edvardsson-Källkvist[3], MD; Sayam Bunpuckdee[12], MD student; Christian Unge[9], MD, PhD; Martin Larsson[13,14], MD, PhD; Björn Johansson[15], MD; Johan Ljungberg[15], MD; Jonas Lindell[16], MD; Johan Hansson[17], MD, PhD; Ola Blennow[1,3,18#¤], MD, PhD, Daniel P Andersson[3,13#] MD, PhD

*Equal contributions

#Equal contributions

¤Corresponding author

Correspondence

Ola Blennow MD, PhD Address: Department of Infectious Diseases, Karolinska University Hospital, Huddinge, 14141 Stockholm Sweden

Email: ola.blennow@regionstockholm.se

Phone: +46739813617

Affiliations:

[1] Department of Internal Medicine, Capio St Göran´s Hospital, Stockholm, Sweden

[2] Functional Area of Emergency Medicine, Karolinska University Hospital Huddinge, Stockholm, Sweden

For peer review only - http://bmjopen.bmj.com/site/about/guidelines.xhtml

[3] Department of Medicine (H7), Karolinska Institutet, Karolinska University Hospital, Huddinge, 141 86 Stockholm, Sweden

[4] Clinical Epidemiology Division, Department of Medicine Solna, Karolinska Institutet, Stockholm, Sweden

[5] Department of Physiology and Pharmacology, Karolinska Institutet, Stockholm, Sweden.

[6] Centre for Clinical Research Västmanland, Uppsala University, Västmanland County Hospital, Västerås, Sweden.

[7] Department of Clinical Sciences, Karolinska Institutet, Danderyd Hospital, Stockholm, Sweden

[8] School of Medical Sciences, Faculty of Medicine, Örebro University, Örebro, Sweden

[9] Department of Internal Medicine, Danderyd Hospital, Stockholm, Sweden

[10] Department of Infectious Diseases, Växjö Central Hospital, Växjö, Sweden

[11] Department in Geriatric Medicine at Karlskoga Hospital, Karlskoga, Sweden

[12] At the time of the study: Department of Medicine (H7), Karolinska Institutet, Stockholm, Sweden

[13] Department of Endocrinology, Karolinska University Hospital, Stockholm, Sweden

[14] Department of Molecular Medicine and Surgery (K1), Karolinska Institutet, Stockholm, Sweden

[15] Department of Infectious Diseases Halland County Hospital Halmstad, Halmstad, Sweden

[16] Department of Infectious Diseases, Visby Hospital, Visby, Sweden

[17] Department of Infectious Diseases, Östersund Hospital, Östersund, Sweden

For peer review only

[18] Department of Infectious Diseases, Karolinska University Hospital, Stockholm, Sweden

Word count: 3288

Abstract word count: 250

For peer review only

For peer review only - http://bmjopen.bmj.com/site/about/guidelines.xhtml

Abstract

Objective: To assess the efficacy of inhaled ciclesonide in reducing the duration of oxygen therapy (an indicator of time to clinical improvement) among adults hospitalized with Covid-19.

Design: Multicenter, randomized, controlled, open-label trial.

Setting: 9 hospitals (3 academic hospitals and 6 non-academic hospitals) in Sweden between June 1, 2020, and May 17, 2021.

Participants: Adults hospitalized with Covid-19 and receiving oxygen therapy.

Intervention: Inhaled ciclesonide 320 µg twice daily for 14 days versus standard care.

Main outcome measures: Primary outcome was duration of oxygen therapy, an indicator of time to clinical improvement. Key secondary outcome was a composite of invasive mechanical ventilation/death.

Results: Data from 98 participants were analyzed (48 receiving ciclesonide and 50 receiving standard care; median (IQR) age, 59.5 (49-67) years; 67 (68%) male). Median (IQR) duration of oxygen therapy was 5.5 (3-9) days in the ciclesonide group and 4 (2-7) days in the standard care group (hazard ratio (HR) for termination of oxygen therapy 0.73 (95% CI 0.47-1.11), with the upper 95% CI being compatible with a 10% relative reduction in oxygen therapy duration, corresponding to a <1-day absolute reduction in a post-hoc calculation). Three participants in each group died/received invasive mechanical ventilation (HR 0.90 (95% CI 0.15-5.32)). The trial was discontinued early due to slow enrollment.

For peer review only - http://bmjopen.bmj.com/site/about/guidelines.xhtml

Conclusions: In hospitalized Covid-19 patients receiving oxygen therapy, this trial ruled out, with 0.95 confidence, a treatment effect of ciclesonide corresponding to more than a one-day reduction in duration of oxygen therapy. Ciclesonide is unlikely to improve this outcome meaningfully.

Strengths and limitations of this study

- This was a multicenter, randomized, controlled, open-label trial comparing treatment with the inhaled corticosteroid ciclesonide 320 μg twice daily for 14 days versus standard care.
- Healthcare providers and participants were not blinded to treatment assignment.
- The trial was terminated early due to slow recruitment.

For peer review only

Introduction

Patients with Covid-19 can develop acute respiratory failure that may require invasive mechanical ventilation, associated with high mortality. The unregulated inflammation in the lungs, poor oxygenation and pulmonary infiltrates characterizing severe Covid-19 have been considered as a type of acute respiratory distress syndrome (ARDS).[1 2]

Prior to the Covid-19 pandemic, studies have indicated that inhaled corticosteroids may reduce the risk of ARDS. In a randomized controlled trial including 61 patients at risk of ARDS, none of the patients assigned to aerosolized budesonide/formoterol vs 7 assigned to placebo developed ARDS,[3] and 6 (20%) and 16 (53%) of the patients, respectively, received mechanical ventilation. In another trial including 60 patients with acute lung injury or ARDS, nebulized budesonide improved oxygenation and peak and plateau airway pressures, and reduced inflammatory markers.[4] Moreover, potentially protective and preventive effects of inhaled corticosteroids for ARDS is supported by animals models of lung injury,[5-8] and *in vitro* studies,[9] and it has been speculated that local administration of the drug in the lung may maximize therapeutic benefits with fewer systemic side effects, as compared with systemic steroids.[3]

Therefore, it could be hypothesized that inhaled corticosteroids may be beneficial for patients with severe Covid-19. The hypothesis is further supported by reports that inhaled corticosteroids reduce the epithelial expression of genes linked to SARS-CoV-2 entry into host cells.[10 11] Among the inhaled corticosteroids, ciclesonide has been identified as a particularly promising treatment as it can suppress replication of SARS-CoV-2 *in vitro*.[12 13]

For peer review only - http://bmjopen.bmj.com/site/about/guidelines.xhtml

While previous randomized controlled trials have assessed the effects of inhaled budesonide[14 15] or ciclesonide[16 17] in non-hospitalized Covid-19 patients, no study has been performed in hospitalized patients with more severe Covid-19.

This open-label randomized controlled trial investigated the effects of inhaled ciclesonide, compared to standard care, in adult patients hospitalized with Covid-19 and requiring oxygen therapy.

For peer review only

Methods

Study design

The HALT Covid-19 (inHALation of ciclesonide for Treatment of Covid-19) trial was a multicenter, open-label randomized controlled trial to assess the efficacy and safety of inhaled ciclesonide for the treatment of hospitalized patients with Covid-19 receiving oxygen therapy. The trial was conducted at 9 hospitals (3 academic hospitals and 6 non-academic hospitals) in Sweden between June 1, 2020, and May 17, 2021.

All participants provided written informed consent. The study was approved by the Swedish Ethical Review Authority (Ethics committee number 2020-02183) and the Swedish Medical Products Agency (Eudra-CT number 2020-001928-34) and registered at clinicaltrials.gov (NCT04381364).

Protocol changes and rationale

For peer review only - http://bmjopen.bmj.com/site/about/guidelines.xhtml

The trial was designed in the beginning of the Covid-19 pandemic. After trial initiation, treatments for, and hospitalization rates of, patients with Covid-19 changed rapidly. Therefore, we made protocol changes (described in detail in the Online Appendix) and the trial was stopped early.

In brief, we increased the number of study centers, removed the upper age limit (≤85 years) for patient inclusion, changed the inclusion criteria from ≤48 hours since hospital admission to ≤48 hours from initiation of oxygen therapy and allowed for patients to be included on the basis of a positive antigen test for SARS-Cov-2. All changes were approved by the Data Monitoring Committee, Ethical Review Authority and the Swedish Medical Products Agency and implemented from December 2020.

In June 2021, 99 patients had been included in the study, a large and increasing proportion of the adult Swedish population had received Covid-19 vaccination and hospitalizations for Covid-19 had dropped substantially. We determined that it was unlikely that the intended sample size would be reached and asked the Data Monitoring Committee to convene for a meeting. Following the recommendation of the Data Monitoring Committee, the study was terminated for futility to meet the targeted enrolment.

Participants

Based on observations from Covid-19 patients treated at the study centers, we expected that 85% of the standard care group would survive and terminate oxygen

For peer review only - http://bmjopen.bmj.com/site/about/guidelines.xhtml

therapy within 30 days (median 8 days). We considered a 25% (2 days) reduction in the duration of oxygen therapy to be a clinically meaningful effect. We estimated that such an effect could be detected with α of 0.05, and 80% power if 446 participants (223 in each group) were enrolled.

Participants were eligible for inclusion if, they (1) were aged ≥18 years, (2) had a polymerase chain reaction confirmed SARS-CoV-2 infection or a positive SARS-CoV-2 antigen test from the upper respiratory tract, (3) were hospitalized at any of the study hospitals and (4) were receiving oxygen therapy, initiated within 48 hours before inclusion. Key exclusion criteria were ongoing treatment with inhaled or oral corticosteroids (previous use was accepted), oxygen therapy with >8 L oxygen/min or >50 % oxygen on nasal high-flow cannula, and ongoing or expected intensive care or palliative care (Online Appendix).

Randomization

Patients were randomized 1:1 in blocks of 8, stratified by sex and hospital to receive ciclesonide or standard care. The randomization sequence was prepared by a statistician not involved in the trial. Treatment allocation was provided through a web-based interface. The participants and the physicians treating them were unblinded to the treatment assignment.

Intervention

The treatment was 320 μg of inhaled ciclesonide (80 μg per actuation, for a total of 4 actuations, or 160 μg per actuation, for a total of 2 actuations) twice daily (total daily dose 640 μg) for 14 days. Ciclesonide was administered using a spacer (L'espace,

For peer review only - http://bmjopen.bmj.com/site/about/guidelines.xhtml

Nordic Infucare, Stockholm Sweden). Participants randomized to ciclesonide received written instructions, including pictures, and practical instructions on how to use the inhalator and spacer; the first dose was taken under supervision. Ciclesonide was then prescribed in the participant's electronic medical record and each given dose during the hospitalization was recorded. Participants discharged before day 14 were instructed to continue the treatment at home for a total treatment duration of 14 days. Participants randomized to standard care did not receive any intervention related to the study. Physicians treating the participants were not given any restrictions concerning treatments during the study period. Participants who had been discharged were contacted by telephone after day 30 for a follow-up interview.

Outcomes

The primary outcome was duration of oxygen therapy (time to termination of oxygen therapy in days) up to 30 days from randomization. Oxygen therapy was defined as terminated on the day after which the patient did not receive oxygen therapy during at least 48 hours, while being alive. This outcome corresponded to clinical improvement for patients receiving oxygen therapy according to the World Health Organization clinical progression scale.[18]

The key secondary outcome was a composite of invasive mechanical ventilation and death up to 30 days after randomization. Other secondary outcomes were each component of the key secondary outcome, admission to an intensive care unit, discharge from the hospital and dyspnea in daily living at 30-35 days after randomization as evaluated by the mMRC (Modified Medical Research Council)

For peer review only - http://bmjopen.bmj.com/site/about/guidelines.xhtml

dyspnea scale. The scale ranges from 0 to 4 with a higher score indicating more severe dyspnea.[19 20]

Data on serious adverse events[21] were collected by review of electronic medical records. Information about non-serious adverse events associated with ciclesonide use (dryness of mouth, nausea and oral candidiasis) was reported using a paper-based reporting form which was filled in by the treating physician. Information about non-serious adverse events occurring after hospital discharge was collected during the follow-up interview.

Data collection

Patient characteristics at baseline (comorbidities, comedications, clinical parameters) and study outcomes were obtained from electronic medical records. Investigators contacted participants after day 30 after randomization to ask them about non-serious adverse events and dyspnea in daily living (study outcome) at day 30-35 after randomization.

Statistical analysis

According to the pre-specified analysis plan in the study protocol, the analyses were performed by an investigator who had not been involved in the enrolment of participants and was blinded to treatment assignment. An intention-to-treat population was used. In the analysis of the duration of oxygen therapy, participants were followed from randomization to termination of oxygen therapy, death, or 30 days after randomization. Kaplan Meier cumulative incidence curves were generated to

For peer review only - http://bmjopen.bmj.com/site/about/guidelines.xhtml

illustrate the cumulative incidence of termination of oxygen therapy in the ciclesonide and standard care groups. A Cox proportional hazard regression model, adjusted for study hospital (Appendix Table 1), age (continuous variable) and sex was used to estimate hazard ratios (HR) with 95% CI for time-to-event outcomes. Proportions and the absolute risk difference with 95% CI were presented for binary outcomes. In a per-protocol analysis of the primary outcome, participants assigned to ciclesonide were censored at the time of discontinuing treatment. The median mMRC score was compared using the Kruskal-Wallis test. A logistic regression model adjusted for study hospital, age and sex was used to compare the likelihood of reporting a mMRC score of 0 (dyspnea only with strenuous exercise).

In an analysis that was not pre-specified, we additionally adjusted the primary outcome analysis for baseline variables, including days since symptom onset, c-reactive protein and white blood count (as continuous variables), and diabetes mellitus, hypertension and hyperlipidemia.

95% CIs of ratios not including 1 and 95% CIs for absolute risk differences not including 0 were considered statistically significant. Secondary outcome analyses were considered hypothesis-generating and no adjustment for multiple testing was made. Analyses were performed using Stata version 16.1 (StataCorp).

Patient and Public involvement

No patients were involved in setting the research question, nor in the design, conduct, or interpretation of the study.

For peer review only - http://bmjopen.bmj.com/site/about/guidelines.xhtml

Results

Of the 99 participants who underwent randomization 48 were assigned to receive ciclesonide and 51 to standard care (Figure 1). One participant in the standard care group withdrew consent and was excluded from the analysis. Ninety-eight patients (48 in the ciclesonide group and 50 in the standard care group) were included in the final analysis. All participants assigned to ciclesonide received the treatment at least once. None of the participants were lost to follow-up. The median age of participants was 59.5 (IQR 49, 67) years, 68% were men and the median duration of symptoms was 9 (IQR 8, 11) days. There were no relevant between-group differences in demographic characteristics, laboratory test results or comorbidities at enrollment (Table 1).

Table 1 Demographic and clinical characteristics of participants at study enrolment.

	**Total** (n=98)	**Ciclesonide** (n=48)	**Standard care** (n=50)
Age, median (IQR)	59.5 (49, 67)	61 (49, 67)	59 (49, 67)
Age ≥70 years, n (%)	78 (80)	37 (77)	41 (82)
Men, n (%)	67 (68)	34 (71)	33 (66)
Days since symptom onset, median (IQR)	9 (8, 11)	9 (7.5, 11.5)	10 (8, 11)
Days since symptom onset: <10 days, n (%)	51 (52)	27 (56)	24 (48)
Body mass index in kg/m^2, median (IQR)	29.7 (25.6, 34.0)	28.7 (25.4, 34.0)	30.6 (26.8, 34.3)
Oxygen flow of oxygen therapy in L/min, median (IQR)	2 (1, 3)	2 (1, 3)	2 (1, 2)
Respiratory rate per minute, median (IQR)	20 (18, 24)	20 (19, 25)	20 (18, 23)
C-reactive protein in mg/L, median (IQR)	100 (56, 142)	103 (62, 164)	91.5 (45.5, 124.5)

For peer review only - http://bmjopen.bmj.com/site/about/guidelines.xhtml

White cell count in x 10^9/L, median IQR	5.7 (4.5, 7.0)	5.3 (4.3, 6.9)	6.1 (4.9, 7.0)
eGFR in mL/min/1.73m^2, median (IQR)	83 (70.5, 90)	81.5 (70, 90)	87 (73, 90)
**Coexisting conditions, n (%)**			
Diabetes mellitus	18 (18)	8 (17)	10 (20)
Hypertension[a]	45 (46)	22 (46)	23 (46)
Hyperlipidemia[b]	27 (28)	12 (25)	15 (30)
Chronic obstructive lung disease	3 (3)	1 (2)	2 (4)
Asthma	8 (8)	6 (13)	2 (4)
Current smoker	12 (12)	6 (13)	6 (12)
Ischemic heart disease	8 (8)	2 (4)	6 (12)
Heart failure	3 (3)	2 (4)	1 (2)
Atrial fibrillation	5 (5)	3 (6)	2 (4)
Cancer	10 (10)	5 (10)	5 (10)
Chronic kidney disease	9 (9)	5 (10)	4 (8)

[a] Diagnosis of hypertension or use of antihypertensive drugs

[b] Diagnosis of hyperlipidemia or use of lipid lowering therapy

Missing values were: n=1 for days since symptom onset, n=20 for body mass index, n=1 for oxygen flow of oxygen therapy, n=1 for body temperature, n=1 for heart rate, n=3 for respiratory rate, n=3 for C-reactive protein, n=7 for white cell count and n=22 for eGFR.

eGFR: estimated glomerular filtration rate

The results of primary and secondary outcome analyses are presented in Table 2. Kaplan-Meier estimates of the median duration of oxygen therapy were 5.5 (IQR 3, 9) days in the ciclesonide group and 4 (2, 7) days in the standard care group. (Figure 2). The HR for termination of oxygen therapy during 30 days following randomization, used to compare ciclesonide vs standard care, showed that ciclesonide treatment was not statistically significantly associated with the duration of oxygen therapy (0.73 (95% CI 0.47 to 1.11)).

For peer review only

The research question that we aimed to assess was whether inhaled ciclesonide, as compared with standard care, could reduce the time to clinical improvement (as indicated by duration of oxygen therapy). While the interpretation of statistically non-significant findings is a recurring and well-known subject of debate in the medical literature, it is generally not recommended to use a binary interpretation based on an arbitrary cut-off for statistical significance[22-25]. This is particularly important in this trial as it was terminated early and thereby underpowered to assess its primary outcome. However, it has been suggested that in trials with statistically non-significant findings, the 95% CIs should be used to rule in or rule out potential effect sizes of the intervention. In this study, we therefore assessed the largest benefit of ciclesonide that was compatible with the confidence interval. Such a benefit was represented by the upper limit of the HR for time to termination of oxygen therapy (a higher HR indicates shorter duration of oxygen therapy for the ciclesonide group), i.e., 1.11. We took the inverse of this HR (1/1.11 = 0.90) to calculate the relative reduction in duration of oxygen therapy that the HR was compatible with (i.e., 1-0.90 = 10% relative reduction). We then multiplied this 10% relative reduction with the absolute duration of oxygen therapy in the standard care group to calculate the corresponding absolute difference in duration of oxygen therapy (10% * 4 days = 0.4 days, which is <1 day). Given the pre-specified minimally clinically important difference of 2 days (which was used for the power calculation of the study), we deemed this best-case difference to be clinically irrelevant.

In the per-protocol analysis, the HR for termination of oxygen therapy during 30 days following randomization was 0.79 (95% CI 0.51 to 1.23). In the additionally adjusted

For peer review only - http://bmjopen.bmj.com/site/about/guidelines.xhtml

analysis (Appendix table 2) the HR for termination of oxygen therapy was 0.68 (95% CI 0.43 to 1.09) (Table 2).

Table 2 Outcomes. All outcomes are recorded during 30 days following randomization unless otherwise indicated.

	Ciclesonide	Standard care	Difference[a]
**Primary outcome**			
Duration of oxygen therapy, median (IQR) days	5.5 (3, 9)	4 (2, 7)	0.73 (0.47 to 1.11)
**Key secondary outcome**			
Death or invasive mechanical ventilation, n (%)	3 (6)	3 (6)	0 (-9 to 10)
Time to death or invasive mechanical ventilation, median (IQR) days	2 (2, 10)	4 (2, 7)	0.90 (0.15 to 5.32)
**Secondary outcomes**			
Death, n (%)	2 (4)	1 (2)	-
Invasive mechanical ventilation, n (%)	1 (2)	3 (6)	-
Admission to an intensive care unit, n (%)	4 (8)	4 (8)	-
mMRC dyspnea scale score at day 30-35, median (IQR)[b]	3 (2, 4)	3 (2, 4)	0.97
mMRC dyspnea scale score 0 at day 30-35, n (%)[b]	4 (9)	7 (15)	0.48 (0.11 to 2.04)
**Per protocol analysis** [c.]			
Duration of oxygen therapy, median (IQR) days	5 (3, 9)	4 (2, 7)	0.79 (0.51 to 1.23)
**Additionally adjusted analysis**[d]			
Duration of oxygen therapy, median (IQR) days	5 (3, 9)	4.5 (2, 7)	0.68 (0.43 to 1.09)

[a.] Differences are expressed as hazard ratios (95% CI) estimated using a Cox proportional hazards model for time to event outcomes and as absolute risk difference (95% CI) in percent for outcomes of absolute risk. The comparison of the mMRC dyspnea score was done using the Kruskal-Wallis test and the difference is expressed as a p-value. The comparison of the likelihood of reporting a mMRC score of 0 was done using a logistic regression model and the difference is expressed as an odds ratio (95% CI). Statistical testing for differences in proportions and time-to-event analyses were not performed for the secondary outcome events, including death, invasive mechanical ventilation, and admission to an intensive care unit due to few events.

For peer review only - http://bmjopen.bmj.com/site/about/guidelines.xhtml

[b.] Not including 1 participant in the standard care group and 2 participants in the ciclesonide group who died within 30 days of randomization and 1 participant in the standard care group and 1 participant in the ciclesonide group with missing data on this outcome.

[c.] In the per-protocol analysis for duration of oxygen therapy, patients assigned to ciclesonide were censored at the time of discontinuing treatment.

[d.] In addition to age, sex and study center, this analysis of duration of oxygen therapy was adjusted for days since symptom onset, c-reactive protein, white blood count (as continuous variables) and diabetes mellitus, hypertension and hyperlipidemia (as categorical variables). The analyses included n=46 in the standard care group and n=45 in the ciclesonide group without missing data on any of the variables included in the model.

In total, 3 (6%) participants assigned to ciclesonide and 3 (6%) participants assigned to standard care experienced the key secondary outcome of mechanical invasive ventilation or death (absolute difference 0% (95% CI -10 to 9%; HR 0.90 (95% CI 0.15 to 5.32)). Median mMRC dyspnea score at 30-35 days after randomization was 3 (IQR 2, 4) in both groups (p-value for difference 0.97) (Table 2).

There were no apparent differences between the groups in treatments that participants received after randomization (Table 3); 26 (54%) of the participants assigned to ciclesonide and 22 (44%) of the participants in the standard care group received treatment with systemic corticosteroids after randomization.

Few serious adverse clinical events occurred during the study. The most frequently reported adverse event was dry mouth (7 (15%) participants in the ciclesonide group and 11 (22%) participants in the standard care group). Two participants assigned to ciclesonide and 0 in the placebo group reported that they experienced oral

For peer review only - http://bmjopen.bmj.com/site/about/guidelines.xhtml

candidiasis (Table 3).

Table 3 Participants' treatments and adverse clinical events through day 30 after randomization.

	**Ciclesonide** (n=48)	**Standard care** (n=50)
**Received treatment, n (%)**		
Systemic corticosteroids	26 (54)	22 (44)
Remdesivir	4 (8)	5 (10)
Low-molecular-weight heparin	45 (94)	45 (90)
Oral anticoagulants	32 (67)	30 (60)
Vasopressors	4 (8)	3 (6)
Non-invasive mechanical ventilation	8 (17)	7 (14)
**Serious clinical events, n (%)**		
Renal failure	2 (4)	3 (6)
Cardiac arrest	1 (2)	0 (0)
New onset atrial fibrillation	0 (0)	1 (2)
Pulmonary embolism	4 (8)	2 (4)
Other thromboembolic events	0 (0)	1 (2)
Sepsis	3 (6)	2 (4)
Other serious event	1 (2)	0 (0)
**Non-serious adverse events, n (%)**		
Nausea	6 (13)	8 (16)
Dry mouth	7 (15)	11 (22)
Oral candidiasis	2 (4)	0 (0)
Other non-serious adverse event	3 (6)	1 (2)

For peer review only - http://bmjopen.bmj.com/site/about/guidelines.xhtml

Some pre-specified analyses were not performed due to small sample size or low number of events. These included statistical testing of differences in proportions and time-to-event analyses for non-key secondary outcomes, including death, invasive mechanical ventilation, and admission to an intensive care unit; the secondary outcome analyses of discharge from hospital; subgroup analyses, and the primary outcome analysis after exclusion of participants who received invasive mechanical ventilation or died.

For peer review only

Discussion

In this randomized open-label, controlled trial, including 98 hospitalized Covid-19 patients with ongoing oxygen therapy, treatment with inhaled ciclesonide did not result in a statistically significant reduction in the duration of oxygen therapy, used as a measure of time to clinical improvement. The trial ruled out, with 0.95 confidence, treatments effects of ciclesonide corresponding to more than a one-day reduction in duration of oxygen therapy.

While previous randomized controlled trials have assessed effects of inhaled corticosteroids, including budesonide[14 15] and ciclesonide[16 17], in non-hospitalized patients with Covid-19, this is the first trial that includes hospitalized patients with more severe forms of the disease. In contrast to our hypothesis, the median duration of oxygen therapy was nominally longer among patients assigned to ciclesonide vs standard care (5.5 vs 4 days; HR for termination of oxygen therapy 0.73 (95% CI 0.47 to 1.11)). As such, the 95% CI indicates that,[24] even in the best case, ciclesonide may reduce the duration of oxygen therapy with only 10% (1-1/1.11; less

For peer review only - http://bmjopen.bmj.com/site/about/guidelines.xhtml

than 1 day in our study) while it may in the worst case result in an over 2-fold increase. Thus, the results of this trial indicate that ciclesonide is unlikely to provide a clinically meaningful beneficial effect on the duration of oxygen therapy in hospitalized Covid-19 patients receiving oxygen therapy.

To date, 2 randomized controlled trials of ciclesonide in non-hospitalized patients with Covid-19 have been presented. In the CONTAIN study,[16] which was terminated early due to slow recruitment, 215 non-hospitalized patients with a median of 3 days symptom duration were randomized to combination treatment with intranasal and inhaled ciclesonide or placebo. No statistically significant difference between the groups was observed for the primary endpoint, resolution of respiratory symptoms at day 7 after randomization, which was reached by 40% of the patients in the treatment group vs 35% in the placebo group (adjusted risk difference of 5.5% (95% CI -7.8% to 18.8%).[16] Six (6%) patients assigned to ciclesonide vs 3 (3%) in the placebo group were hospitalized within 14 days; none died. In another clinical trial of ciclesonide, including 400 non-hospitalized patients with Covid-19,[17] randomization to ciclesonid vs placebo did not result in a reduced time to alleviation of all Covid-19 related symptoms. However, in secondary outcome analyses, patients assigned to ciclesonide had fewer emergency department visits or hospital admissions for reasons related to COVID-19 (odds ratio, 0.18, 95% CI, 0.04 to 0.85).

In addition, 2 randomized clinical trials of the inhaled corticosteroid budesonide in non-hospitalized patients with Covid-19 have been presented. The STOIC trial was an open-label trial comparing inhaled budesonide vs standard care in 146 Covid-19 patients with mild symptoms.[14] Compared to standard care, budesonide treatment

For peer review only - http://bmjopen.bmj.com/site/about/guidelines.xhtml

led to a statistically significant reduction in Covid-19-related emergency department assessment and hospitalization (difference in proportions 0.123 (95% CI 0.043 to 0.218).[14] Furthermore, budesonide treatment was associated with 1 day shorter time to clinical recovery. The PRINCIPLE trial was another open-label trial that included 4700 primary care patients at high risk of developing severe Covid-19 (1073 randomized to budesonide treatment; 1988 to standard care; 1639 to other treatments).[15] Compared to standard care, randomization to budesonide led to a shorter time to self-reported recovery (difference 2.94 days (95% Bayesian credible interval 1.19 to 5.12) and a reduced likelihood of hospital admission or death, although the results for the latter outcomes did not meet the superiority threshold.

Taken together, the previous studies indicate that inhaled corticosteroids might be useful for preventing deterioration of Covid-19 in non-hospitalized patients with mild symptoms. It is possible that the low likelihood of benefit associated with ciclesonide treatment observed in our study reflects the more severe pulmonary inflammation in our study population, as indicated by the need for hospitalization with oxygen therapy and a median symptom duration of 9 days: at such stages of disease progression, it could be speculated that pulmonary administration of corticosteroids may not suffice to confer benefit and that systemic treatment is needed. Accordingly, in the Recovery trial of hospitalized Covid-19 patients,[26] dexamethasone treatment reduced risk of death and the time to discharge from hospital, with these benefits primarily being observed among patients receiving oxygen therapy or invasive mechanical ventilation at baseline.

Similar to other clinical trials including patients with Covid-19,[15 26 27] we used a

For peer review only - http://bmjopen.bmj.com/site/about/guidelines.xhtml

pragmatic, open-label design. With this design, we intended to assess the effect of adding ciclesonide to standard care, rather than to examine the effect of ciclesonide compared to placebo. The research question that our study aimed to answer was "what is the effect of using ciclesonide as an addition to standard care as compared with standard care alone?" While this is a research question of relevance to clinical decision-making, the open-label design and the possible expectations of effect among both patients[28] and physicians might have affected the outcomes in our study, including when to terminate oxygen therapy. Another limitation of our study is that we were unable to recruit the intended number of patients due to the substantial decrease in hospitalized Covid-19 patients in Sweden during 2021. Importantly, the study could not provide much information regarding the key secondary outcome of death or invasive mechanical intervention. Further research in hospitalized Covid-19 patients is needed to determine the potential effect of ciclesonide treatment on these outcomes. Moreover, it is a possibility that effects of ciclesonide differ as compared to other inhaled corticosteroids (e.g., budesonide). Patients were instructed to use ciclesonide without a spacer after discharge from the hospital; this may have affected drug delivery. Finally, results from the Recovery Trial were released 5 weeks after the initiation of our study and around half of the patients in both the ciclesonide group and the control group received systemic corticosteroids after randomization. Further studies would be needed to assess the comparative effectiveness and safety of ciclesonide vs systemic corticosteroids.

Conclusions

In this open-label randomized controlled trial in patients hospitalized with Covid-19 and receiving oxygen therapy, the findings indicated that treatment with ciclesonide

For peer review only - http://bmjopen.bmj.com/site/about/guidelines.xhtml

vs standard care is unlikely to result in a clinically meaningful reduction in the duration of oxygen therapy.

Acknowledgements

We would like to thank the following individuals that did not qualify for authorship but contributed to the study: Dr Oscar Bakhouch (Skaraborg Hospital), Dr Eva-Marie Boman and Dr Anders Lundqvist (Southern Älvsborg Hospital).

Contributorship statement

DB, PU, PT, OB and DPA conceived the study and were responsible for the methods. OB and DPA were responsible for the study conduct. DB, PU, OB and DPA were responsible for the financing. PT validated the data. PU performed the main analysis. DPA, PU and PT wrote the original draft of the manuscript. All authors wrote, reviewed, and edited the manuscript. OB and DPA supervised the study. DB, DPA, PU, PT and OB were responsible for administration of the project. DPA and OB are the guarantors. DB, PT, AK, EW, SA, SW, OE, AN, AE, JG, JEK, SB, CU, ML, BJ, JL, JLI, JH, OB and DPA enrolled participants in the study. The corresponding author attests that all listed authors meet authorship criteria and that no others meeting the criteria have been omitted.

Competing interests

This study received non-financial support from COVIS Pharma (study drug donation). The authors have no conflict of interest to disclose.

For peer review only - http://bmjopen.bmj.com/site/about/guidelines.xhtml

For peer review only

Funding/Support

This study was funded by the Swedish Heart and Lung Foundation (Number 20200421), The Axel and Margaret Ax:son Johnson Foundation (N/A), CIMED (N/A), and Strategic Research Program at Karolinska Institutet (Number 961507), the Stockholm County Council (Number: 954970, 963296, 962029), and the Västmanland County Council (Grant nr LTV-938409). PU was supported by grants from the Strategic Research Program in Epidemiology at Karolinska Institutet (N/A), and a Faculty Funded Career Position at Karolinska Institutet (N/A).

The funders had no role in the study design, conduct, collection, management, analysis, interpretation of data, writing or reviewing the manuscript or decision to submit the manuscript for publication. The study drug was donated by COVIS Pharma but COVIS pharma did not participate in any other part of the study.

Data sharing statement

Data are available upon resonable request

Ethics Approval

All participants provided written informed consent. The study was approved by the Swedish Ethical Review Authority (Ethics committee number 2020-02183) and the Swedish Medical Products Agency (Eudra-CT number 2020-001928-34) and registered at clinicaltrials.gov (NCT04381364).

References

For peer review only - http://bmjopen.bmj.com/site/about/guidelines.xhtml

1. Gibson PG, Qin L, Puah SH. COVID-19 acute respiratory distress syndrome (ARDS): clinical features and differences from typical pre-COVID-19 ARDS. *Med J Aust* 2020;213(2):54-56 e1. doi: 10.5694/mja2.50674 [published Online First: 2020/06/24]
2. Bos LDJ. COVID-19-related Acute Respiratory Distress Syndrome: Not So Atypical. *Am J Respir Crit Care Med* 2020;202(4):622-24. doi: 10.1164/rccm.202004-1423LE [published Online First: 2020/06/25]
3. Festic E, Carr GE, Cartin-Ceba R, et al. Randomized Clinical Trial of a Combination of an Inhaled Corticosteroid and Beta Agonist in Patients at Risk of Developing the Acute Respiratory Distress Syndrome. *Crit Care Med* 2017;45(5):798-805. doi: 10.1097/CCM.0000000000002284 [published Online First: 2017/02/28]
4. Mohamed HS, Meguid MM. Effect of nebulized budesonide on respiratory mechanics and oxygenation in acute lung injury/acute respiratory distress syndrome: Randomized controlled study. *Saudi J Anaesth* 2017;11(1):9-14. doi: 10.4103/1658-354X.197369 [published Online First: 2017/02/22]
5. Jansson AH, Eriksson C, Wang X. Effects of budesonide and N-acetylcysteine on acute lung hyperinflation, inflammation and injury in rats. *Vascul Pharmacol* 2005;43(2):101-11.

For peer review only - http://bmjopen.bmj.com/site/about/guidelines.xhtml

doi: 10.1016/j.vph.2005.03.006 [published Online First: 2005/06/22]

6. Forsgren PE, Modig JA, Dahlback CM, et al. Prophylactic treatment with an aerosolized corticosteroid liposome in a porcine model of early ARDS induced by endotoxaemia. *Acta Chir Scand* 1990;156(6-7):423-31. [published Online First: 1990/06/01]
7. Wang J, Zhang L, Walther SM. Administration of aerosolized terbutaline and budesonide reduces chlorine gas-induced acute lung injury. *J Trauma* 2004;56(4):850-62. doi: 10.1097/01.ta.0000078689.45384.8b [published Online First: 2004/06/10]
8. Walther S, Jansson I, Berg S, et al. Pulmonary granulocyte accumulation is reduced by nebulized corticosteroid in septic pigs. *Acta Anaesthesiol Scand* 1992;36(7):651-5. doi: 10.1111/j.1399-6576.1992.tb03537.x [published Online First: 1992/10/01]
9. Yamaya M, Nishimura H, Deng X, et al. Inhibitory effects of glycopyrronium, formoterol, and budesonide on coronavirus HCoV-229E replication and cytokine production by primary cultures of human nasal and tracheal epithelial cells. *Respir Investig* 2020;58(3):155-68. doi: 10.1016/j.resinv.2019.12.005 [published Online First: 2020/02/26]

For peer review only - http://bmjopen.bmj.com/site/about/guidelines.xhtml

10. Peters MC, Sajuthi S, Deford P, et al. COVID-19-related Genes in Sputum Cells in Asthma. Relationship to Demographic Features and Corticosteroids. *Am J Respir Crit Care Med* 2020;202(1):83-90. doi: 10.1164/rccm.202003-0821OC [published Online First: 2020/04/30]

11. Milne S, Li X, Yang CX, et al. Inhaled corticosteroids downregulate SARS-CoV-2-related genes in COPD: results from a randomised controlled trial. *Eur Respir J* 2021;58(1) doi: 10.1183/13993003.00130-2021 [published Online First: 2021/04/03]

12. Jeon S, Ko M, Lee J, et al. Identification of Antiviral Drug Candidates against SARS-CoV-2 from FDA-Approved Drugs. *Antimicrob Agents Chemother* 2020;64(7) doi: 10.1128/AAC.00819-20 [published Online First: 2020/05/06]

13. Kimura H, Kurusu H, Sada M, et al. Molecular pharmacology of ciclesonide against SARS-CoV-2. *J Allergy Clin Immunol* 2020;146(2):330-31. doi: 10.1016/j.jaci.2020.05.029 [published Online First: 2020/07/01]

14. Ramakrishnan S, Nicolau DV, Jr., Langford B, et al. Inhaled budesonide in the treatment of early COVID-19 (STOIC): a phase 2, open-label, randomised controlled trial. *Lancet*

For peer review only

For peer review only - http://bmjopen.bmj.com/site/about/guidelines.xhtml

Respir Med 2021;9(7):763-72. doi: 10.1016/S2213-2600(21)00160-0 [published Online First: 2021/04/13]

15. Yu LM, Bafadhel M, Dorward J, et al. Inhaled budesonide for COVID-19 in people at high risk of complications in the community in the UK (PRINCIPLE): a randomised, controlled, open-label, adaptive platform trial. *Lancet* 2021;398(10303):843-55. doi: 10.1016/S0140-6736(21)01744-X [published Online First: 2021/08/14]
16. Ezer N, Belga S, Daneman N, et al. Inhaled and intranasal ciclesonide for the treatment of covid-19 in adult outpatients: CONTAIN phase II randomised controlled trial. *BMJ* 2021;375:e068060. doi: 10.1136/bmj-2021-068060 [published Online First: 2021/11/04]
17. Clemency BM, Varughese R, Gonzalez-Rojas Y, et al. Efficacy of Inhaled Ciclesonide for Outpatient Treatment of Adolescents and Adults With Symptomatic COVID-19: A Randomized Clinical Trial. *JAMA Intern Med* 2021 doi: 10.1001/jamainternmed.2021.6759 [published Online First: 2021/11/23]
18. Characterisation WHOWGotC, Management of C-i. A minimal common outcome measure set for COVID-19 clinical research. *Lancet Infect Dis* 2020;20(8):e192-e97. doi: 10.1016/S1473-3099(20)30483-7 [published Online First: 2020/06/17]

For peer review only - http://bmjopen.bmj.com/site/about/guidelines.xhtml

19. Mahler DA, Wells CK. Evaluation of clinical methods for rating dyspnea. *Chest* 1988;93(3):580-6. doi: 10.1378/chest.93.3.580 [published Online First: 1988/03/01]
20. Bestall JC, Paul EA, Garrod R, et al. Usefulness of the Medical Research Council (MRC) dyspnoea scale as a measure of disability in patients with chronic obstructive pulmonary disease. *Thorax* 1999;54(7):581-6. doi: 10.1136/thx.54.7.581 [published Online First: 1999/06/22]
21. Directive 2001/20/EC of the European Parliament and of the Council of 4 April 2001 on the approximation of the laws, regulations and administrative provisions of the member states relating to the implementation of good clinical practice in the conduct of clinical trials on medicinal products for human use. *Med Etika Bioet* 2002;9(1-2):12-9. [published Online First: 2005/11/10]
22. Altman DG, Bland JM. Absence of evidence is not evidence of absence. *BMJ* 1995;311(7003):485. doi: 10.1136/bmj.311.7003.485 [published Online First: 1995/08/19]
23. Harrell F. Statistical Errors in the Medical Literature. Statistical Thinking. https://www.fharrell.com/post/errmed/index.html (accessed Oct 24, 2022). ed, 2017.

For peer review only

24. Hawkins AT, Samuels LR. Use of Confidence Intervals in Interpreting Nonstatistically Significant Results. *JAMA* 2021;326(20):2068-69. doi: 10.1001/jama.2021.16172 [published Online First: 2021/11/24]

25. Greenland S, Senn SJ, Rothman KJ, et al. Statistical tests, P values, confidence intervals, and power: a guide to misinterpretations. *Eur J Epidemiol* 2016;31(4):337-50. doi: 10.1007/s10654-016-0149-3 [published Online First: 2016/05/23]

26. Group RC, Horby P, Lim WS, et al. Dexamethasone in Hospitalized Patients with Covid-19. *N Engl J Med* 2021;384(8):693-704. doi: 10.1056/NEJMoa2021436 [published Online First: 2020/07/18]

27. Investigators R-C, Gordon AC, Mouncey PR, et al. Interleukin-6 Receptor Antagonists in Critically Ill Patients with Covid-19. *N Engl J Med* 2021;384(16):1491-502. doi: 10.1056/NEJMoa2100433 [published Online First: 2021/02/26]

28. Wechsler ME, Kelley JM, Boyd IO, et al. Active albuterol or placebo, sham acupuncture, or no intervention in asthma. *N Engl J Med* 2011;365(2):119-26. doi: 10.1056/NEJMoa1103319 [published Online First: 2011/07/15]

For peer review only

For peer review only

For peer review only - http://bmjopen.bmj.com/site/about/guidelines.xhtml

Figure 1 Flow diagram for study participants.

Figure 2 Time to termination of oxygen therapy during 30 days after randomization.

For peer review only

For peer review only - http://bmjopen.bmj.com/site/about/guidelines.xhtml

Figure 1

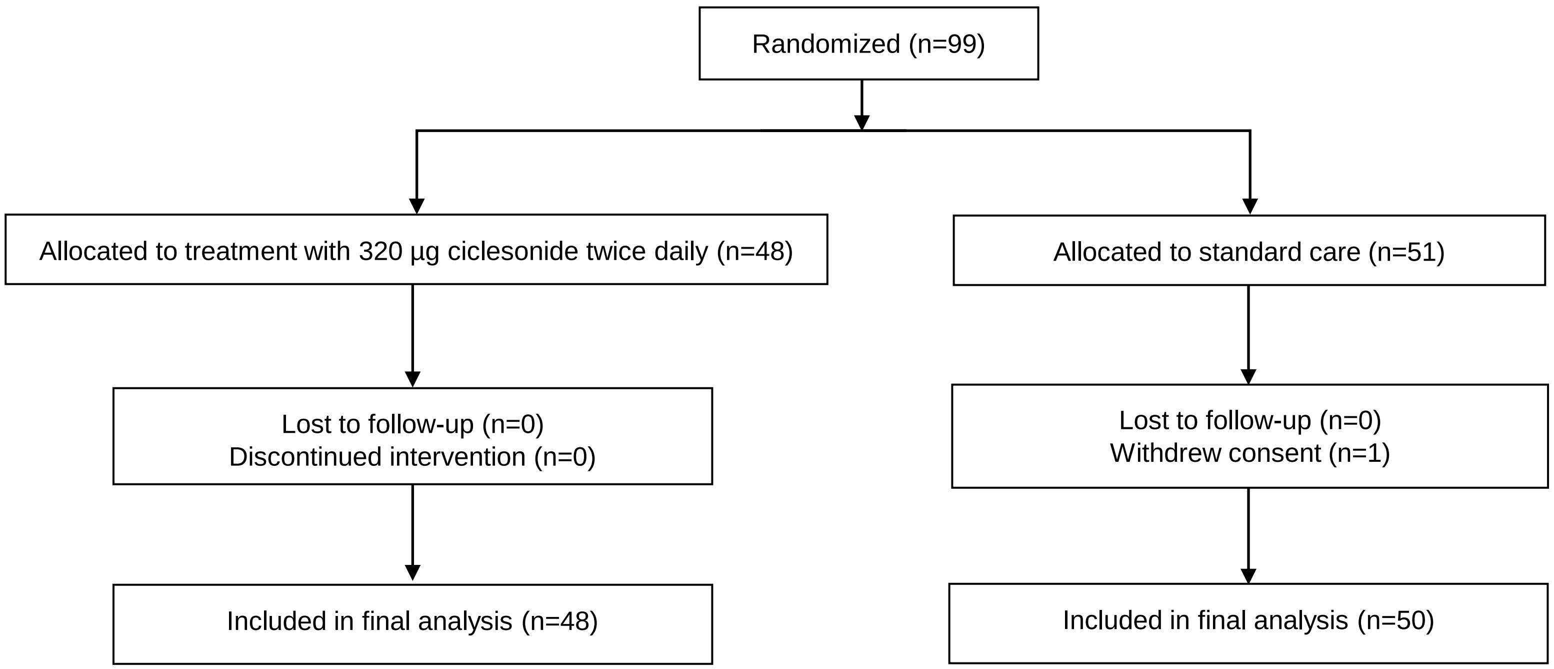
Randomized (n=99)
Allocated to treatment with 320 µg ciclesonide twice daily (n=48)
Allocated to standard care (n=51)
Lost to follow-up (n=0)
Discontinued intervention (n=0)
Lost to follow-up (n=0)
Withdrew consent (n=1)
Included in final analysis (n=48)
Included in final analysis (n=50)

Figure 2 Time to termination of oxygen therapy.

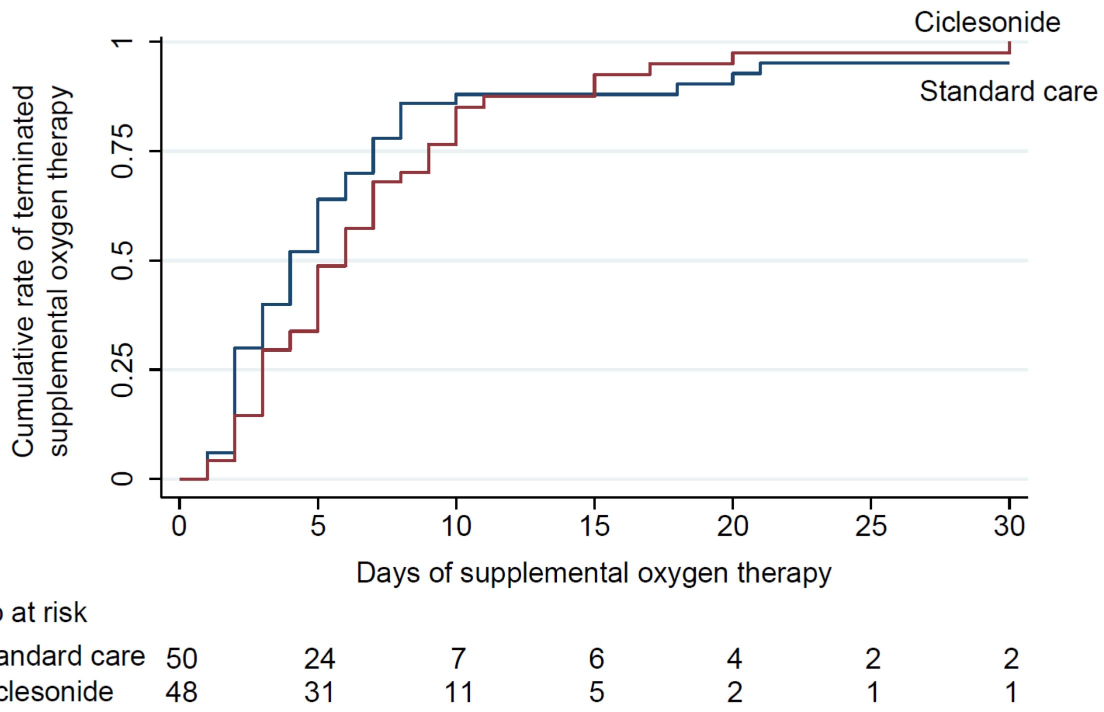

ew only

ONLINE APPENDIX

Brodin D, Tornhammar P, Ueda P, Krifors A, Westerlund E, Athlin S, Wojt S, Elvstam O, Neumann A, Elshani A, Giesecke J, Edvardsson J, Bunpuckdee S, Unge C, Larsson M, Johansson B, Ljungberg J, Lindell J, Hansson J, Blennow O, Andersson DP. Inhaled Ciclesonide in Adults Hospitalized with Covid-19: a Randomized Controlled Open-label Trial (HALT Covid-19).

For peer review only

Protocol changes and rationale

The trial was designed in the beginning of the covid-19 pandemic when data from randomized clinical trials of Covid-19 treatment were scarce. After trial initiation, treatments for patients with Covid-19 and hospitalization rates of such patients changed rapidly. Therefore, we made changes to the protocol and the trial was stopped early.

5 weeks after the start of patient inclusion in our study, in July 2020, the Recovery Collaborative group presented preliminary data[1] showing protective effects of dexamethasone treatment in patients hospitalized for covid-19; a subgroup analysis of this study indicated that the effect was driven by patients receiving invasive mechanical ventilation or oxygen therapy. These data, in combination with local experience from treating patients with Covid-19,[2] led to most patients receiving oxygen therapy with ≥ 4 L oxygen/min at the study hospitals being treated with systemic corticosteroids. As use of systemic corticosteroids was an exclusion criterion, the change in practice made a large proportion of the Covid-19 patients ineligible for participation.

Initially the trial was conducted at 4 hospitals. To increase the inclusion rate, 9 additional hospitals were included as study sites, although only 5 of them ended up recruiting patients to the study. We also removed the previous upper age limit of 85 years for inclusion and allowed for inclusion of patients based on a positive antigen test for SARS-CoV-2. Moreover, because some patients may start receiving oxygen therapy before hospital admission (e.g., at nursing homes before being transported

For peer review only

to the hospital) or a period after hospital admission (e.g., if the patient's condition deteriorated) and we aimed to include patients shortly after initiation of such therapy, we changed the inclusion criteria from hospitalization within 48 hours prior to enrollment to initiation of oxygen therapy no longer than 48 hours prior to enrollment.

All changes were approved by the Data Monitoring Committee, Ethical Review Authority and the Swedish Medical Products Agency and implemented from December 2020.

In June 2021, when 99 patients had been included in the study, a large and increasing proportion of the adult Swedish population had received vaccination for Covid-19. The number of patients hospitalized with Covid-19 had dropped substantially and there were none to only a few Covid-19 patients admitted to the study hospitals per week. We determined that it was unlikely that we would reach the intended sample size and asked the Data Monitoring Committee to convene for a meeting. Following the recommendation of the Data Monitoring Committee, the study was terminated early due to expected futility to meet total enrolment.

Inclusion and exclusion criteria[a]

Participants were eligible for inclusion if, at the time of study inclusion, they (1) were aged ≥18 years, (2) had a polymerase chain reaction confirmed SARS-CoV-2 infection or a positive antigen test for SARS-CoV-2, (3) were hospitalized at any of the study hospitals and (4) were receiving oxygen therapy with not more than 48 hours having passed since initiation of this treatment.

Patients were not eligible for inclusion if they (1) had a history of hypersensitivity to ciclesonide or other substances included in the treatment, (2) received ongoing treatment with inhaled or oral corticosteroids, ketokonazol, itrakonazol, ritonavir or nelfinavir, (3) received >8 L oxygen/min or >50 % oxygen with nasal high-flow therapy, (4) were receiving or under consideration for palliative care or had an expected survival of less than 72 h, (5) were expected to be admitted to an intensive care unit within 48 h, (6) had active or inactive pulmonary tuberculosis, severe liver failure (Child-Pugh C), pulmonary arterial hypertension or fibrosis, cognitive or physical impairment, (7) had insufficient language skills to understand information given about the study, (8) had been included in a clinical trial within 30 days, or (9) were women and pregnant, breastfeeding or did not agree to take highly effective contraceptive measures while receiving treatment plus an additional 7 days.

[a] The presentation of these inclusion and exclusion criteria have been modified for readability as compared with the version presented in the study protocol.

For peer review only

Appendix table 1 Number of participants included in the final study population by study center.

Study center	*n participants*
Danderyd Hospital	26
Capio S:t Göran Hospital	24
Karolinska University Hospital	21
Västmanland County Hospital	13
Örebro University Hospital[a]	6
Växsjö Central Hospital[a]	3
Halland County Hospital[a]	2
Östersund Hospital[a]	2
Visby Hospital[a]	1

[a] In the analyses adjusted for study center, these hospitals were categorized into one group.

For peer review only

Appendix table 2 Additionally adjusted model.

Variable	Hazard ratio (95% CI) for termination of oxygen therapy
Ciclesonide (vs standard care)	0.68 (0.43 to 1.09)
Age (per year increase)	0.97 (0.95 to 0.99)
Female (vs male)	0.81 (0.46 to 1.40)
Days since symptom onset (per day increase)	0.99 (0.93 to 1.06)
C-reactive protein (per mg/L increase)	1.00 (1.00 to 1.00)
White cell count (per 10^9/L increase)	1.07 (0.95 to 1.19)
Diabetes	0.84 (0.44 to 1.58)
Hypertension	1.42 (0.79 to 2.56)
Hyperlipidemia	0.86 (0.45 to 1.64)

The model was also adjusted for study center.

For peer review only

References

1. Group RC, Horby P, Lim WS, et al. Dexamethasone in Hospitalized Patients with Covid-19. *N Engl J Med* 2021;384(8):693-704. doi: 10.1056/NEJMoa2021436 [published Online First: 2020/07/18]

2. Kan B, Ahl M, Blennow O, et al. *Lakartidningen* 2020;117 [published Online First: 2020/10/07]

For peer review only

CONSORT 2010 checklist of information to include when reporting a randomised trial*

Section/Topic	Item No	Checklist item	Reported on page No
**Title and abstract**			
	1a	Identification as a randomised trial in the title	1
	1b	Structured summary of trial design, methods, results, and conclusions (for specific guidance see CONSORT for abstracts)	3
**Introduction**			
Background and objectives	2a	Scientific background and explanation of rationale	5,6
	2b	Specific objectives or hypotheses	5-7
**Methods**			
Trial design	3a	Description of trial design (such as parallel, factorial) including allocation ratio	6-9
	3b	Important changes to methods after trial commencement (such as eligibility criteria), with reasons	6,7,9
Participants	4a	Eligibility criteria for participants	7,8
	4b	Settings and locations where the data were collected	6,7
Interventions	5	The interventions for each group with sufficient details to allow replication, including how and when they were actually administered	6-11
Outcomes	6a	Completely defined pre-specified primary and secondary outcome measures, including how and when they were assessed	9-11
	6b	Any changes to trial outcomes after the trial commenced, with reasons	6,7
Sample size	7a	How sample size was determined	6-9
	7b	When applicable, explanation of any interim analyses and stopping guidelines	6,7
Randomisation:			
Sequence generation	8a	Method used to generate the random allocation sequence	8
	8b	Type of randomisation; details of any restriction (such as blocking and block size)	8
Allocation concealment mechanism	9	Mechanism used to implement the random allocation sequence (such as sequentially numbered containers), describing any steps taken to conceal the sequence until interventions were assigned	8
Implementation	10	Who generated the random allocation sequence, who enrolled participants, and who assigned participants to interventions	8
Blinding	11a	If done, who was blinded after assignment to interventions (for example, participants, care providers, those	8,11

For peer review only

		assessing outcomes) and how	
	11b	If relevant, description of the similarity of interventions	-
Statistical methods	12a	Statistical methods used to compare groups for primary and secondary outcomes	10,11
	12b	Methods for additional analyses, such as subgroup analyses and adjusted analyses	10,11
**Results**			
Participant flow (a diagram is strongly recommended)	13a	For each group, the numbers of participants who were randomly assigned, received intended treatment, and were analysed for the primary outcome	11-13
	13b	For each group, losses and exclusions after randomisation, together with reasons	11-13
Recruitment	14a	Dates defining the periods of recruitment and follow-up	6,7
	14b	Why the trial ended or was stopped	7
Baseline data	15	A table showing baseline demographic and clinical characteristics for each group	Yes
Numbers analysed	16	For each group, number of participants (denominator) included in each analysis and whether the analysis was by original assigned groups	Yes
Outcomes and estimation	17a	For each primary and secondary outcome, results for each group, and the estimated effect size and its precision (such as 95% confidence interval)	Yes
	17b	For binary outcomes, presentation of both absolute and relative effect sizes is recommended	-
Ancillary analyses	18	Results of any other analyses performed, including subgroup analyses and adjusted analyses, distinguishing pre-specified from exploratory	10,11
Harms	19	All important harms or unintended effects in each group (for specific guidance see CONSORT for harms)	13
**Discussion**			
Limitations	20	Trial limitations, addressing sources of potential bias, imprecision, and, if relevant, multiplicity of analyses	13-17
Generalisability	21	Generalisability (external validity, applicability) of the trial findings	13-17
Interpretation	22	Interpretation consistent with results, balancing benefits and harms, and considering other relevant evidence	13-17
**Other information**			
Registration	23	Registration number and name of trial registry	6
Protocol	24	Where the full trial protocol can be accessed, if available	-
Funding	25	Sources of funding and other support (such as supply of drugs), role of funders	18,19

*We strongly recommend reading this statement in conjunction with the CONSORT 2010 Explanation and Elaboration for important clarifications on all the items. If relevant, we also recommend reading CONSORT extensions for cluster randomised trials, non-inferiority and equivalence trials, non-pharmacological treatments, herbal interventions, and pragmatic trials. Additional extensions are forthcoming: for those and for up to date references relevant to this checklist, see www.consort-statement.org.

For peer review only - http://bmjopen.bmj.com/site/about/guidelines.xhtml
